# DIM5/KMT1 controls fungal insect pathogenicity and genome stability by methylation of histone H3K4, H3K9 and H3K36

**DOI:** 10.1080/21505594.2021.1923232

**Published:** 2021-05-06

**Authors:** Kang Ren, Ya-Ni Mou, Sen-Miao Tong, Sheng-Hua Ying, Ming-Guang Feng

**Affiliations:** a MOE Laboratory of Biosystems Homeostasis & Protection, Institute of Microbiology, College of Life Sciences, Zhejiang University, Hangzhou, China; b College of Agricultural and Food Science, Zhejiang A & F University, Lin’an, Zhejiang, China

**Keywords:** Entomopathogenic fungi, histone h3k9-specific methyltransferase, multisite h3 methylation, gene regulation, pathgogenicity and virulence, stress response, asexual cycle

## Abstract

Mono-, di- and tri-methylation of histone H3 Lys 9, Lys 4, and Lys 36 (H3K_me1/me2/me3) required for mediation of DNA-based cellular events in eukaryotes usually rely upon the activities of histone lysine methyltransferases (KMTs) classified to the KMT1, KMT2, and KMT3 families, respectively. Here, an H3K9-specific DIM5/KMT1 orthologue, which lacks a C-terminal post-SET domain and localizes mainly in nucleus, is reported to have both conserved and noncanonical roles in methylating the H3 core lysines in *Beauveria bassiana*, an insect-pathogenic fungus serving as a main source of wide-spectrum fungal insecticides. Disruption of *dim5* led to abolishment of H3K9me3 and marked attenuation of H3K4me1/me2, H3K9me1/me2 and H3K36me2. Consequently, the Δ*dim5* mutant lost the whole insect pathogenicity through normal cuticle infection, and was compromised severely in virulence through cuticle-bypassing infection (hemocoel injection) and also in a series of cellular events critical for the fungal virulence and lifecycle *in vivo* and *in vitro*, including reduced hyphal growth, blocked conidiation, impeded proliferation *in vivo*, altered carbohydrate epitopes, disturbed cell cycle, reduced biosynthesis and secretion of cuticle-degrading enzymes, and increased sensitivities to various stresses. Among 1,201 dysregulated genes (up/down ratio: 712:489) associated with those phenotypic changes, 92 (up/down ratio: 59:33) encode transcription factors and proteins or enzymes involved in posttranslational modifications, implying that the DIM5-methylated H3 core lysines could act as preferential marks of those transcription-active genes crucial for global gene regulation. These findings uncover a novel scenario of DIM5 and its indispensability for insect-pathogenic lifestyle and genome stability of *B. bassiana*.

## Introduction

DNA-based cellular processes take place in the nucleosomes wrapped by octamers of core histones, such as histone H3 [1]. Histone methylation is one of postranslational modifications (PTMs) that serve as regulatory mechanisms of DNA replication, repair, recombination, transcription and RNA processing, and is implemented by adding one, two or three methyl groups to specific lysines, such as H3 lysines 4, 9, and 36 (H3K4, H3K9, and H3K36), for mono- (me1), di- (me2) and tri-methylation (me3) under the actions of histone lysine methyltransferases (KMTs) [2–4]. The methylation of H3K9, H3K4, and H3K36 (H3K_me) relies upon the activities of KMTs classified to the KMT1, KMT2, and KMT3 families, respectively [5]. H3K9me is catalyzed by the Suv39 family KMTs, which are featured by a SET [Su(var)3–9, Enhancer-of-zeste and Trithorax] domain, known as Su(var)3–9 proteins in *Drosophila melanogaster*, SUV39H1 and SUV39H2 in mammals and cryptic loci regulator 4 (Clr4) in *Schizosaccharomyces pombe* [6–8], and associated with the silent regions of both euchromatin and heterochromatin [8–11] responsible for transcriptional repression [12–14]. Such KMT1/SUV39 proteins also possess a cysteine-rich pre-SET domain required for a specificity to H3K9 [8]. In *S. pombe*, deposition of H3K9me for heterochromatin establishment relies upon Clr4, which has chromodomain and disordered region to bind the nucleosome core [15]. Unlike H3K9me, H3K4me is related exclusively to actively transcribed genes [2,16], and catalyzed by the H3K4 methylase COMPASS (COMplex of Proteins Associated with SET1), which comprises SET1/KMT2 and other components in *Saccharomyces cerevisiae* [17]. SET1/KMT2-catalyzed H3K4me is often associated with the early transcribed regions of active genes [18–21], and regulated by CxxC zinc finger protein 1 (Cfp1) binding the COMPASS [22]. H3K4me depends on the activity of COMPASS binding mono-ubiquitinated histone H2B and free nucleosome with its catalytic module [23], and can define the transcriptional status of a genomic region and protect the genome from the replication stress and instability induced by transcription-replication conflicts in *S. cerevisiae* [24]. H3K36me is catalyzed by SET2/KMT3 and also associated with gene activity [3,4], but involved mechanisms were elusive until recently. SET2 interacts with H3 αN, H3 tail and H2A C-terminal tail for stabilization of DNA in the unwrapped conformation capable of positioning the enzyme to specifically methylate H3K36 and also with ubiquitin to aid the positioning [25,26]. An autoinhibitory domain of SET2 is revealed by isolating several highly clustered, dominant SET2 mutations to suppress H3K36me defect in the mutant of the transcription elongation factor Spt6 and considered as a conserved mechanism underlying the multiple SET2 interactions to ensure an effect of H3K36me on actively transcribed chromatin [27]. Transcription-active genes contain the nucleosomes marked by H3K4me3, H3K9/14 acetyl and H3K36me3 [28]. In *S. cerevisiae*, KMTs essential for H3K4me and H3K36me regulate alternative polyadenylation at the sites where precursor mRNA is cleaved and polyadenylated [29]. However, only a subset of genes encoding development-regulated proteins carry the mark of H3K4me3 instead of H3K36me3 [28,30], and their expression is presumably regulated by RNA polymerase II (Pol II) general elongation factors [30]. These studies unveil that the KMTs of each family play a conserved role in the key lysine-specific H3me across eukaryotes.

In filamentous fungi, gene silencing and transposon control rely on DNA methylation [31]. Approximately 2% of genomic cytosines can be methylated in *Neurospora crassa* [32]. Fungal DNA methylation is controlled by the DNA methyltransferase DIM2, the H3K9 methyltransferase DIM5/KMT1, heterochromatin protein 1 (HP1), and additional subunits as components of the DIM5 complex DCDC (DIM5/7/9, CUL4/DDB1 Complex), as reviewed recently [33]. Formation of heterochromatin at the transposon relics of repeat-induced point mutations requires recruitment of DCDC components by DIM5 that regulates H3K9me3 [34,35], followed by the binding of HP1 to H3K9me3 via its chromodomain to recruit DIM2 [36]. In *N. crassa*, DNA methylation at the locus of the frequency clock gene *frq* depends on not only DIM2 [37] but also DIM5 and HP1 [38]. Despite very low or barely detectable levels, fungal DNA methylation has been associated with the silencing of transposable elements and gene expression [39–41]. Altered DNA methylation is associated with important functions of filamentous fungi [33]. For example, DNA methylation inhibits transcription elongation in *N. crassa* and *Magnaporthe oryzae* [42,43]. In *Botrytis cinerea*, loss-of-function mutation of DIM5 (BcDIM5) resulted in nearly abolished H3K9me3 and reduced plant pathogenicity as well as marked defects in hyphal growth, conidiation and sclerotia formation although such defects were not present in the absence of *hp1* or *dim2* [44]. In *Fusarium verticillioides*, H3K9me3 was largely attenuated by *dim5* disruption, leading to defects in conidiation and perithecium production, attenuated virulence, increased osmotolerance and hyper-phosphorylated Hog1 [45]. These studies demonstrate a vital role of DIM5-dependent H3K9me3 in fungal development, virulence and gene expression.

The H3K_me-dependent gene expression and phenotypes are of special merits for the pest control potential of fungal insect pathogens since the potential relies upon fungal virulence, stress tolerance and developmental traits favoring massive production of qualified conidia as active ingredients of fungal insecticides [46–48]. In *Metarhizium robertsii*, abolishment of H3K4me by loss-of-function mutation of SET1/KMT2 resulted in blocked appressorial formation, compromised insect pathogenicity, and differential expression of 1,498 genes considered to be KMT2 dependent [49]. However, pleiotropic effect of H3K_me on the fungal potential against arthropod pests remains understood insufficiently. Aside from KMT2, more KMTs required for H3K9me and H3K36me and their biological effects have not been explored yet in insect-pathogenic fungi. This study sought to elucidate roles of DIM5/KMT1 in *Beauveria bassiana* as a main source of wide-spectrum fungal insecticides by phenotypic and transcriptomic analyses of *dim5* mutants. Unexpectedly, we found not only a conserved activity of DIM5 to H3K9me3, as seen in yeast and filamentous fungi [15,44,45], but also its noncanonical activities to H3K4me and H3K36me usually mediated by SET1/KMT2 and SET2/KMT3, respectively [3–5]. The DIM5-dependent multisite H3me proved essential for the fungal asexual cycle *in vivo* and *in vitro* and genomic stability, as presented below.

## Results

### Sequence features and phylogenetic relationships of fungal DIM5 orthologues

Orthologous DIM5 (NCBI accession EJP62838) identified from the *B. bassiana* genome [50] with the query sequence of *N. crassa* DIM5 (XP_957479) or *S. pombe* Clr4 (NP_595186) consists of 412 amino acids (46.78 kDa) and is encoded by a nucleotide sequence of 3,401 bp (locus tag BBA_08225) containing five introns (2,162 bp in total). Revealed by SMART domain analysis at http://smart.embl-heidelberg.de/, this enzyme shares both pre-SET (residues 23–142) and SET (residues 150–291) domains with the queries and the orthologues in other ascomycetes (Fig. S1a). However, its long C-terminal tail lacks a 16-aa post-SET domain residing in the C-termini of those orthologues in all other examined fungi except two *Cordyceps* species, which fall into Cordycipitaceae as does *B. bassiana*. Further domain analysis at https://www.ncbi.nlm.nih.gov/Structure/ revealed the presence of multiple post-SET clues in a SET domain-containing region (residues 148–306) of *B. bassiana* DIM5 (Table S1). It shares higher sequence identity with the orthologues (64–87%) in Hypocreales, including entomopathogenic *Cordyceps* and *Metarhzium* (Clavicipitaceae) and nonentomopathogenic *Fusarium* and *Trichoderma*, than those (29–63%) in other ascomycetes (Fig. S1b). The absence of a C-terminal post-SET domain implies that DIM5 could have some special activity and function in Cordycipitaceae. This speculation was clarified in *B. bassiana* as follows.

## *Transcription, localization and catalytic activity of DIM5 in* B. bassiana

**Figure 1.**
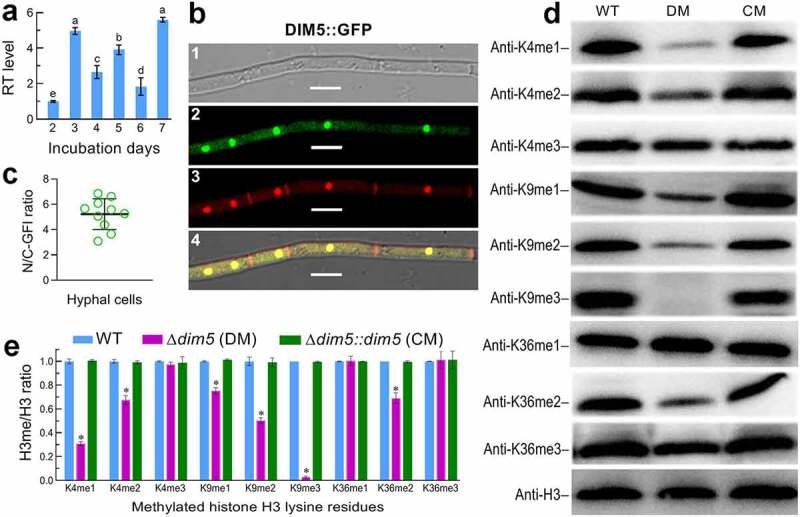
Transcript profile, subcellular localization and catalytic activity of DIM5 in *B. bassiana*. (a) Relative transcript (RT) levels of *dim5* in the SDAY cultures of a wild-type strain (WT) during a 7-day incubation at an optimal regime of 25°C and L:D 12:12 with respect to the standard at the end of a 2-day incubation. (b) Microscopic images (scales: 5 μm) for subcellular localization of GFP-tagged DIM5 fusion protein (DIM5:GFP) expressed in WT. Hyphae were collected from a 3-day-old SDBY culture stained with a nuclear dye (shown in red). Panels 1, 2, 3 and 4 denote bright, expressed (green), stained and merged views of the same field. (c) Ratios of nuclear versus cytoplasmic green fluorescence intensities (N/C-GFI) of the fusion protein assessed from the cells of 10 hyphae. (d) Western blots for mono-, di and tri-methylated signals of H3K4, H3K9 and H3K36 in the nuclear protein extracts isolated from the 3-day-old SDBY cultures of the WT, Δ*dim5* (DM) and Δ*dim5::dim5* (CM) strains. Aliquots of 40 μg protein extracts were probed with appropriate antibodies (detailed in Table S3). (e) Ratios of methylated lysines versus nuclear H3 signal intensities (H3me/H3 ratio) quantified from the blots of three proteins samples per strain. Significant differences from Tukey’s HSD tests are marked with different lowercase letters (*P*< 0.05 in (**a**) or asterisk (*P*< 0.001 in **(e)**). Error bars: SDs of the means from three independent replicates

The transcription of *dim5* in the wild-type strain *B. bassiana* ARSEF 2860 (designated WT) grown at the optimal regime of 25°C in a light/dark (L:D) cycle of 12:12 h was consistently up-regulated with respect to the standard level on day 2 during a 7-day incubation on SDAY (Sabouraud dextrose agar plus yeast extract) plates, and reached differential peaks on days 3, 5, and 7 (Figure 1(a)). Green fluorescence-tagged DIM5 fusion protein expressed in the WT strain accumulated much more heavily in the nuclei than in the cytoplasm of hyphae stained with a nuclear dye after collection from a 3-day-old SDBY (i.e., agar-free SDAY) culture, as revealed by laser scanning confocal microscopy (LSCM) (Figure 1(b)). The mean ratio of nuclear versus cytoplasmic green fluorescence intensities (N/C-GFI) measured from the cells of 10 hyphae was up to 5.23 (Figure 1(c)), indicating a main localization of DIM5 in the nuclei.

To explore DIM5 activity to H3me, *dim5* was disrupted in the WT strain via homologous recombination of its 5′ and 3′ coding/flanking fragments separated by *bar* marker and complemented into an identified Δ*dim5* mutant by integrating ectopically a cassette comprising its full-length DNA sequence with flank regions (4,491 bp in total) and *sur* marker, as described in Methods. The expected recombinant events in the mutants were verified through PCR and Southern blot analyses (Fig. S2) with paired primers and amplified probe (Table S2). As a consequence of *dim5* disruption, H3K9me1, H3K9me2 and H3K9me3 were increasingly attenuated to a hardly detectable level in the nuclear protein extracts isolated from hyphal cultures and probed with appropriate (anti-mono-, di- and tri-methyl-histone H3 Lys 9) antibodies (Table S3) through Western blot analyses although all tested strains showed similar H3 levels in the nuclei (Figure 1(d)). Unexpectedly, H3K4me1, H3K4me2 and H3K36me2 in the Δ*dim5* extracts were also attenuated in comparison to those in the extracts of control (WT and complemented) strains. The ratios of lysine-specific signals over those of nuclear H3 were lowered significantly (Tukey’s HSD, *P*< 0.01) by 25% for H3K9me1, 50% for H3K9me2, 97% for H3K9me3, 69% for H3K4me1, 33% for H3K4me2, and 31% for H3K36me2 in the Δ*dim5* mutant, and the lowered ratios were well restored by targeted *dim5* complementation into the mutant (Figure 1(e)). These data highlighted not only a conserved activity of DIM5 to H3K9me in *B. bassiana*, as seen in plant-pathogenic fungi [44,45], but its noncanonical activities to H3K4me1/me2 and H3K36me2. The DIM5-dependent multisite H3me suggested an unusual role of DIM5 in sustaining the fungal genome stability and lifecycle.

## *Indispensability of* dim5 *for infection cycle*

**Figure 2.**
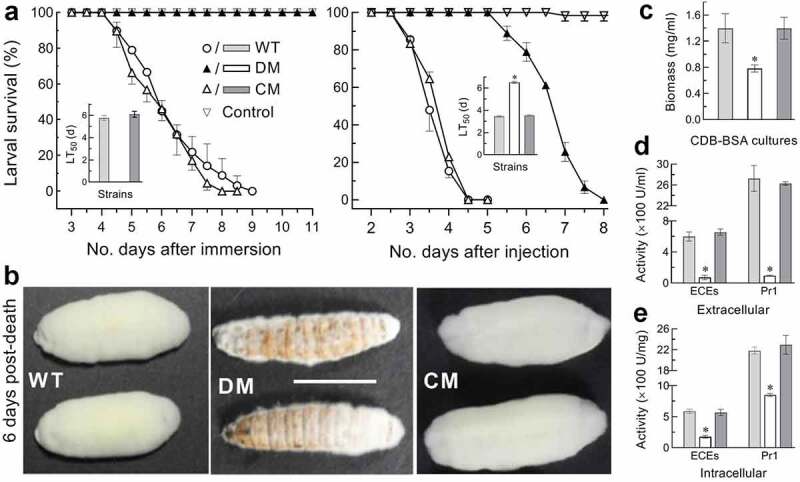
Indispensability of *dim5* for pathogenicity and virulence. (a) Time-survival percentages of *G. mellonella* larvae after topical application (immersion) of a 10^7^ conidia/ml suspension for normal cuticle infection (NCI) or intrahaemocoel injection of ~500 conidia per larva for cuticle-bypassing infection (CBI) and LT_50_ estimates made from the resultant time-mortality trends. Note that the Δ*dim5* mutant (DM) caused no mortality at all via NCI and showed a great delay of lethal action via CBI in comparison to two control (WT and CM) strains. (b) Images (scale: 10 mm) of fungal outgrowths on cadavers 6 days after their death from CBI. (c–e) Biomass levels of 3-day-old CDB-BSA cultures grown at 25°C and total activities of extracellular enzymes (ECEs) and Pr1 proteases quantified from the supernatants (extracellular or secreted) and protein extracts (intracellular or synthesized) of the cultures, respectively. *P< 0.01 in Tukey’s HSD tests. Error bars: SDs of the means from three independent replicates

In standardized bioassays, the control strains caused 100% mortality of *Galleria mellonella* larvae (instar III) within 9 days after topical application (immersion) of a 10^7^ conidia/ml suspension for normal cuticle infection (NCI) and within 5 days after intrahaemocoel injection of ~500 conidia (5 μl of a 10^5^ conidial/ml suspension) per larva for cuticle-bypassing infection (CBI) (Figure 2(a)). In contrast, the Δ*dim5* mutant caused no mortality at all via NCI, and its lethal action was greatly prolonged via CBI, which resulted in median lethal time (LT_50_) estimates of 6.5 and 3.5 days for the Δ*dim5* and WT strains against the model insect respectively. The Δ*dim5* virulence abolished via NCI and greatly compromised via CBI indicated an indispensability of DIM5 for both insect pathogenicity and certain virulence-related cellular events after entry into the host hemocoel.

Aerial conidiation on the surfaces of cadavers from the larvae died from mycosis is critical for fungal infection cycle and also for fungal dispersal/survival in host habitats. Thus, we observed fungal outgrowths on the cadavers maintained at the optimal regime after death from CBI. The control strains produced a heavy layer of outgrowths completely covering the cadavers 6 days post-death, contrasting to very sparse Δ*dim5* outgrowths not covering most of cadaver surfaces (Figure 2(b)). Revealed by microscopic examination, conidia were abundant in the outgrowths of each control strain, yielding an estimate of ~5 × 10^8^ conidia per cadaver 8 days post-death, but hardly found in the Δ*dim5* outgrowths (data not shown). The sparse outgrowths and incapable conidiation of Δ*dim5* on the cadavers reinforced an essential role for DIM5 in the fungal infection cycle and dispersal/survival in host habitats, and also suggested an impaired ability for hyphae to penetrate through the insect cuticle for outgrowth after host death and also to enter host hemocoel during early infection.

Either hyphal invasion into host body for successful infection or outgrowth for conidiation on insect cadavers relies upon cuticular penetration under the actions of extracellular (proteolytic, chitinolytic and lipolytic) enzymes [51], including subtilisin-like Pr1 family proteases collectively required for cuticle degradation [52]. Thus, total activities of extracellular enzymes (ECEs) and Pr1 proteases were quantified from the supernatants and protein extracts of hyphal cultures, which were generated by shaking 50 ml aliquots of a 10^6^ conidia/ml suspension for 3 days in Czapek-Dox broth (CDB) containing sole nitrogen source of 0.3% bovine serum albumin (BSA) as an enzyme inducer. Compared to the WT strain, the Δ*dim5* mutant showed a biomass level decreased by 44% (Figure 2(c)) but total ECEs and Pr1 activities reduced by 88% and 97% in the supernatants (Figure 2(d)) and 70% and 61% in the protein extracts, respectively (Figure 2(e)). Deducting the effect of *dim5* disruption on the biomass level, the ECE and Pr1 activities were reduced by 44% and 53% at extracellular (secreted) level, and the reduced secretion was largely due to blocked biosynthesis of those enzymes in fungal cells. These data implicated an important role for DIM5 in biosynthesis and secretion of cuticle-degrading enzymes essential for cuticular penetration in the courses of initial infection and post-death outgrowth for conidiation on cadavers.

**Figure 3.**
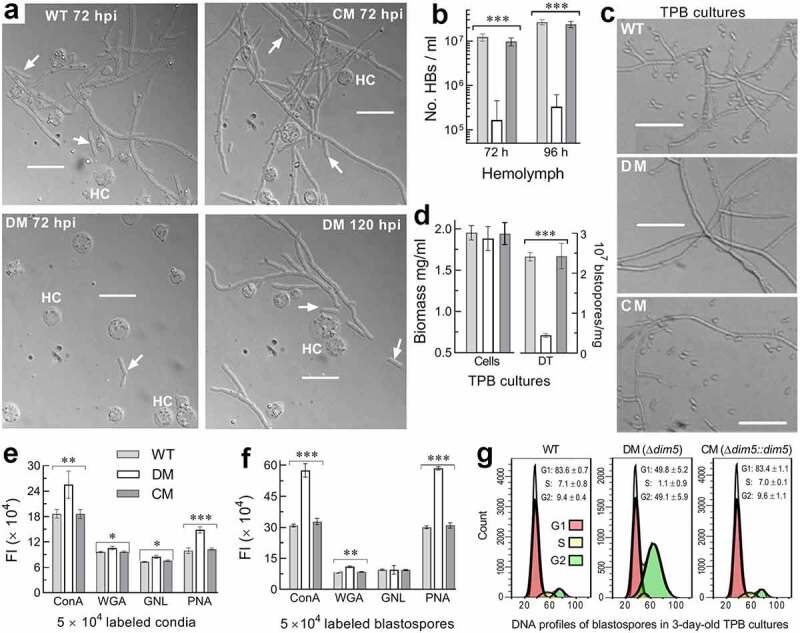
Essential roles of *dim5* in post-infection cellular events crucial for virulence. (a) Microscopic images (scale: 20 μm) of hyphal bodies (arrowed) and host hemocytes (HC) in the hemolymph samples taken from the *G. mellonella* larvae surviving 72 and 144 h post-injection (hpi). (b) Concentrations of hyphal bodies (HBs) measured from the samples taken 72 and 96 hpi. (c) Microscopic images (scale: 20 μm) of hyphae and blastospores formed in the submerged cultures generated by 3-day shaking incubation of a 10^6^ conidia/ml suspension in TPB mimicking insect hemolymph. (d) Biomass levels and dimorphic transition (DT) rates assessed from the 3-day-old TPB cultures. (e, f) Fluorescence intensities (FL) indicating the contents of carbohydrate epitopes on the surfaces of conidia (used for culture initiation) and blastospores (collected from the 3-day-old cultures) labeled with the fluorescent lectins ConA, WGA, GNL and PNA respectively. (g) Distribution of cell cycle phases (G1, G2 and S) revealed by fluorescence-activated cell sorter analysis of 5 × 10^4^ DNA-stained blastospores. *P*< 0.05*, 0.01** or 0.001*** in Tukey’s HSD tests (**b, d–f**). Error bars: SDs of the means from three independent replicates

Upon entry into insect hemocoel, hyphae turn into unicellular thin-wall hyphal bodies (blastospores) to accelerate intrahaemocoel proliferation by yeast-like budding until host death from mycosis development [53–55]. For insight into the attenuated Δ*dim5* virulence via CBI, hemolymph samples taken from surviving larvae 72 h post-injection were examined under a microscope. The control strains produced abundant hyphal bodies in the hemolymph samples whereas hyphal bodies formed by the mutant were rarely observed until 120 h post-injection (Figure 3(a)). Consequently, mean concentration of hyphal bodies formed by the mutant in the samples decreased by 98.6% and 98.8% at 72 and 96 h post-injection, respectively, compared to the corresponding estimates of 1.2 × 10^7^ and 2.7 × 10^7^ cells/ml hemolymph formed by the WT strain (Figure 3(b)). Next, submerged cultures of each strain were initiated by shaking incubation of a 10^6^ conidia/ml suspension in a trehalose-peptone broth (TPB) mimicking insect hemolymph [55,56]. Microscopic examination of samples from 3-day-old TPB cultures revealed that the control strains produced many more blastospores than the mutant (Figure 3(c)). The mean yield of blastospores was 82% lower in the cultures of Δ*dim5* than of the WT strain despite a similarity of their biomass levels (Figure 3(d)).

Both the delayed development of hyphal bodies *in vivo* and the reduced production of blastospores *in vitro* in the absence of *dim5* demonstrated a severe blockage of dimorphic transition, namely hypha-blastospore transition required for hemocoel colonization and *vice versa* for fungal outgrowth after host death. For further insight into these changes, fluorescent lectin binding assays were carried out to reveal possible differences in conidial and blastospore surfaces between the mutant and control strains since spore surfaces are featured by carbohydrate epitopes comprising pathogen-associated molecule patterns (PAMPs) to be perceived by host PAMP-recognition receptors and hence associated with fungal response to host immune defense [57]. Based on the fluorescence intensity changes of labeled Δ*dim5* versus WT conidia, the contents of α-glucose and α-N-acetylglucosamine (GlcNAc) labeled by concanavalin A (ConA), β-GlcNAc and sialic acid residues labeled by wheat germ agglutinin (WGA), mannose residues labeled by *Galanthus nivalis* lectin (GNL) and β-galactose residues labeled by peanut agglutinin (PNA) were elevated by 36%, 10%, 17% and 49% on conidial surfaces of Δ*dim5* respectively (Figure 3(e)). The ConA-, PNA- and WGA-labeled epitopes on blastospore surfaces of the mutant also increased by 86%, 95% and 26%, respectively, despite an unaffected content of its GNL-labeled mannose residues (Figure 3(f)). In addition, the cell cycle of blastospores in the TPB cultures of Δ*dim5* relative to WT showed a markedly shortened G1 phase, a hardly detectable S phase and a greatly prolonged G2 phase (Figure 3(g)), implicating that the cell cycle was disturbed severely in the absence of *dim5*.

All of phenotypic changes in the absence of *dim5* were well restored by targeted *dim5* complementation, indicating an essential role for DIM5 in maintenance of cellular processes and events required for the insect-pathogenic lifecycle of *B. bassiana*. Particularly, both the altered epitopes and the disturbed cell cycle hint at greater pressure of host immune response on colonization of host hemocoel by the Δ*dim5* mutant than the control strains.

## *Essential role of* dim5 *in asexual cycle* in vitro

**Figure 4.**
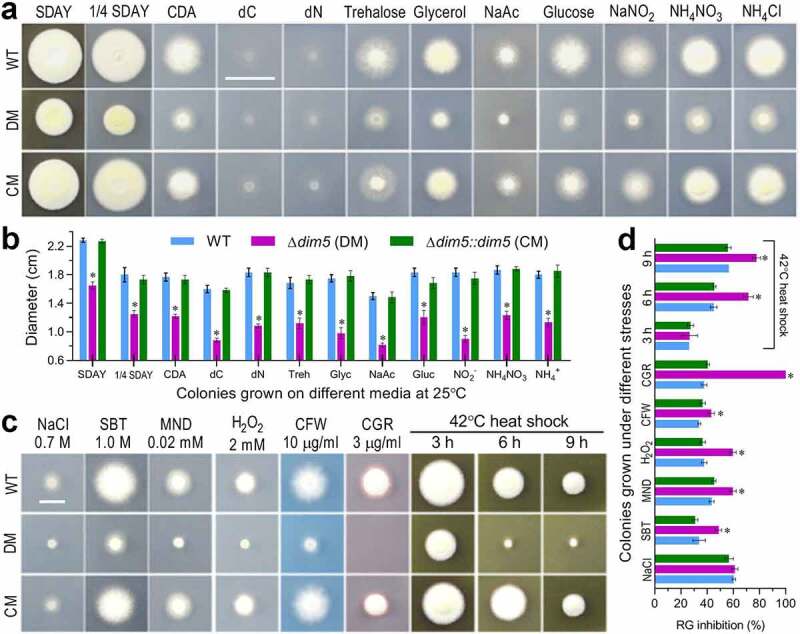
Impact of *dim5* disruption on radial growth under normal and stressful conditions. (a, b) Images (scale: 2 cm) and diameters of fungal colonies incubated at the optimal regime of 25°C and L:D 12:12 for 8 days on rich (SDAY and 1/4 SDAY) and minimal [CDA and CDAs amended with different carbon or nitrogen sources and removal of carbon (dC) or nitrogen (dN) source] media. (c, d) Images (scale: 1 cm) and relative growth (RG) inhibition (%) of fungal colonies incubated at the optimal regime for 8 days on CDA plates containing the indicated concentrations of NaCl, sorbitol (SBT), menadione (MND), H_2_O_2_, calcofluor white (CFW) and Congo red (CGR) respectively, or incubated for 6 days of growth recovery at the optimal regime after exposing 2-day-old SDAY colony to a 42°C heat shock for 3, 6 or 9 h. Each colony was initiated by spotting 1 μl of a 10^6^ conidia/ml suspension. *P< 0.001 (b) or 0.05 (d) in Tukey’s HSD tests. Error bars: SDs of the means from three independent replicates

For a fungal insect pathogen, hyphal invasion into host body relies upon a capability of hyphal growth on oligotrophic insect body surfaces after the germination of conidia attached to insect cuticle and also of cell tolerance to stress cues generated from host immune responses during cuticular penetration and hemocoel colonization [46,47]. Thus, each strain was grown on the plates of rich SDAY, 1/4 SDAY (one-fourth strength of each SDAY nutrient), minimal CDA (i.e., CDB plus agar) and CDAs amended with different carbon or nitrogen sources by spotting 1 μl aliquots of a 10^6^ conidia/ml suspension for colony initiation. After an 8-day incubation at the optimal regime, the Δ*dim5* mutant was compromised in radial growth on all tested media (Figure 4(a)). Diameter measurements revealed that the mutant colonies diminished by 28% on SDAY, 31% on 1/4 SDAY and 31–50% on CDAs amended with the tested carbon or nitrogen sources or by the deletion of carbon or nitrogen source for starvation (Figure 4(b)). The mutant growth was also more suppressed than those of the control strains on the plates of CDA supplemented with different chemical stressors or exposed to a 42°C heat shock for 6 or 9 h during an 8-day incubation at the optimal regime (Figure 4(c)). Based on percent changes of relative growth inhibition (Figure 4(d)), the mutant became hypersensitive to cell wall perturbing stress of Congo red with its sensitivity increased by 62% in comparison to the WT estimate. The mutant also showed moderate, but significant, elevation (9–22%) in sensitivity to the cell wall antagonist calcofluor white, the oxidants menadione and H_2_O_2_, and the noncation osmotic agent sorbitol despite its null response to the osmotic salt NaCl. Also shown in Figure 4d, exposing 2-day-old SDAY colonies to the heat shock for 6 and 9 h resulted in 26% and 21% more suppressed growth recovery of the mutant than of the WT strain at 25°C.

**Figure 5.**
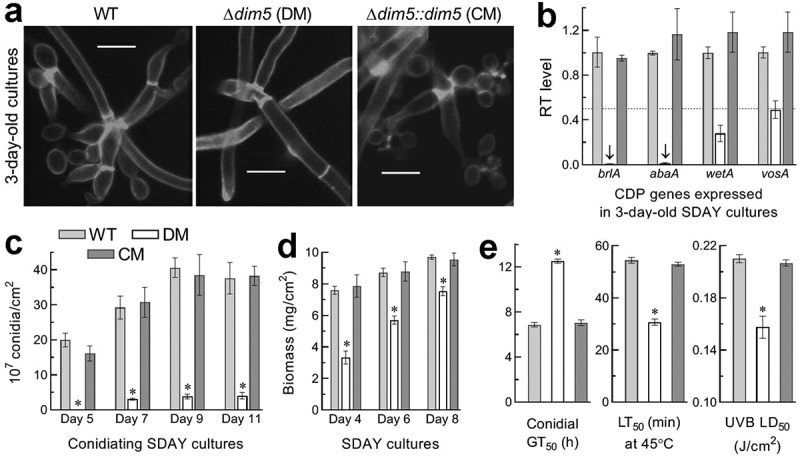
Impact of *dim5* disruption on conidial production and quality. (a) Microscopic images (scale: 5 μm) for conidiation status of samples (stained with calcofluor white) taken from the 3-day-old SDAY cultures, which were initiated by spreading 100 μl of a 10^7^ conidia/ml suspension per plate and incubated at the optimal regime of 25°C and L:D 12:12. Note the rachises (conidiophores) and conidia formed by the control (WT and CM) strains but not by the Δ*dim5* mutant, the hyphae of which remained undifferentiated yet. (b) Relative transcript (RT) levels of three CDP genes and downstream *vosA* in the 3-day-old SDAY cultures of *dim5* mutants with respect to the WT standard. The dashed line denotes a significant level of one-fold (50%) down-regulation. (c, d) Conidial yields and biomass levels measured from the SDAY cultures during a 11-day incubation at the optimal regime. (e) The parameters of conidial quality indicated by GT_50_ (h) for the time of 50% germination at 25°C (viability), LT_50_ (min) for tolerance to a wet-heat stress at 45°C and LD_50_ (J/cm^2^) for resistance to UVB irradiation (weighted wavelength: 312 nm) respectively. *P< 0.01 (d) or 0.001 (c, e) in Tukey’s HSD tests. Error bars: SDs of the means from three independent replicates

Aerial conidiation and conidial maturation mediated by central developmental pathway (CDP) in *B. bassiana* [58,59] determine conidial yield and quality affecting germination and growth rates. For insight into conidiation status *in vitro*, each strain was incubated at the optimal regime for up to 11 days on the SDAY plates spread with 100 μl aliquots of a 10^7^ conidia/ml suspension for culture initiation. At the early developmental stage of a 3-day incubation, zigzag rachises (conidiophores) and conidia formed in the cultures of control stains but not in the Δ*dim5* cultures, in which hyphae were not differentiated yet (Figure 5(a)). At the same stage, the key CDP genes *brlA* and *abaA* required for aerial conidiation and submerged blastospore production [58] were hardly detectable in the Δ*dim5* cultures, accompanied by 72% and 51% repression of the downstream genes *wetA* and *vosA* essential for both conidiation and conidial maturation [59] (Figure 5(b)). As a consequence, the mean of conidial yields in the WT cultures reached 20 × 10^7^ conidia/cm^2^ on day 5, increased to 29 × 10^7^ conidia/cm^2^ on day 7, and stabilized to ~40 × 10^7^ conidia/cm^2^ afterward, contrasting to the Δ*dim5* yield not measurable on day 5 and reduced consistently by ~90% on days 7–11 (Figure 5(c)). Biomass levels measured from the cultures of Δ*dim5* versus WT during the period were averagely reduced by 56% on day 4, and the reduction diminished to 35% on day 6 and to 22% on day 8 (Figure 5(d)). In Δ*dim5*, apparently, severe conidation defect was attributed much more to impeded CDP activation than to reduced biomass level. Next, features indicative of conidial quality were examined. As an index of viability, median time (GT_50_) for 50% germination at optimal 25°C was prolonged by 5.6 h in Δ*dim5* compared to 6.9 h for the WT strain (left panel in Figure 5(e)). In the mutant, median lethal time (LT_50_) for conidial tolerance to a wet-heat stress at 45°C and medial lethal dose (J/cm^2^) for conidial resistance to UVB irradiation were decreased by 44% and 25% (middle and right panels in Figure 5(e)), respectively.

All of the above phenotypic changes were restored by targeted *dim5* complementation into the Δ*dim5* mutant. The results indicated an essential role for DIM5 in sustaining phenotypes crucial for the fungal asexual cycle *in vitro*, including conidiation capacity, conidial quality, and hyphal growth under normal and stressful conditions. In particular, impeded CDP activation and severely compromised conidiation in the mutant cultures suggested a main cause of conidiation level hardly measured in the sparse outgrowths of the mutant on insect cadavers.

## *Genome-wide regulatory role of* dim5

**Figure 6.**
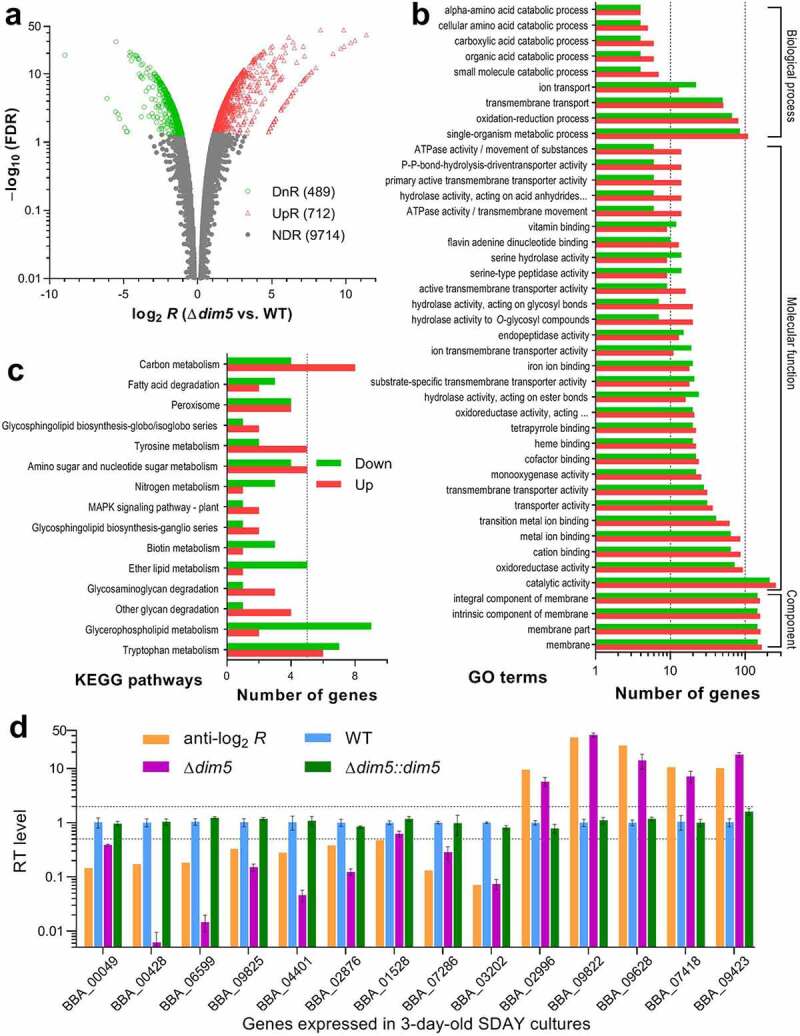
Genome-wide regulatory role of DIM5 in *B. bassiana*. (a) Distributions of log_2_ *R*(Δ*dim5*/WT) and FDR values for all genes significantly up-regulated (UpR; log_2_ *R*≥ 1, FDR < 0.05), down-regulated (DnR; log_2_ *R*≤ –1, FDR < 0.05) or not differentially regulated (NDR; log_2_ *R*< 1 or > –1, FDR ≥ 0.05 if log_2_ *R*> 1 or < –1) in the transcriptomes generated from three 3-day-old SDAY cultures (replicates) of Δ*dim5* and WT grown at the optimal regime of 25°C and L:D 12:12. (b, c) Counts of differentially expressed genes significantly enriched to GO function classes (only main GO terms shown) and KEGG pathways, respectively. (d) Comparison of anti-log_2_ *R*values of 14 differentially expressed genes with relative transcript (RT) levels of them in the 3-day-old SDAY cultures of *dim5* mutants with respective to the WT standard. The upper and lower dashed lines denote the significant levels of log_2_ *R*= 1 and log_2_ *R =* −1, respectively. Error bars: SDs of the means from three cDNA samples analyzed via qPCR with paired primers (Table S8)

To gain in-depth insight into essential role of *dim5* in the fungal lifecycle *in vivo* and *in vitro*, three 3-day-old SDAY cultures (replicates) of Δ*dim5* and WT grown at the optimal regime were used in transcriptomic analysis. The resultant dataset contained 10,915 genes mapped to the *B. bassiana* genome or unable to be mapped. Among those, 1,201 differentially expressed genes (DEGs) were identified at the significant levels of both log_2_ *R*≥ 1.06 or ≤ –1.06 and false discovery rate (FDR) < 0.05, including 489 down- and 712 up-regulated (Figure 6(a), Table S4). The identified DEGs comprised 11.59% of the whole genome, suggesting a reliance of their transcription on DIM5.

Revealed by Gene Ontology (GO) analysis, the identified DEGs were enriched to three function classes containing 108 GO terms at the significance of *p*< 0.05 (Table S5). Illustrated with main GO terms in Figure 6(b), cellular component class comprised 1,241 DEGs (up/down ratio: 652:589), including 1,227 enriched to four membrane-related terms and only 14 others enriched to cell wall and external encapsulating structure. Enriched to molecular function class were 65 GO terms containing 2,032 DEGs (up/down ratio: 1,118:914), including mainly catalytic activity, oxidoreductase activity, cation binding, metal ion binding, transition metal ion binding, transporter activity, transmembrane transporter activity, monooxygenase activity, cofactor binding, heme binding, tetrapyrrole binding, hydrolase activity acting on ester bonds, substrate-specific transmembrane transporter activity, and iron ion binding. The enriched class of biological process comprised 641 DEGs (up/down ratio: 360:280) sorted to 36 GO terms, top 10 of which were single-organism metabolic process, oxidation-reduction process, transmembrane transport, ion transport, small molecule catabolic process, organic acid catabolic process, carboxylic acid catabolic process, cellular amino acid catabolic process, alpha-amino acid catabolic process, and aminoglycan catabolic process. Notably, most of the identified DEGs were simultaneously enriched to different function classes. In contrast, Kyoto Encyclopedia of Genes and Genomes (KEGG) analysis resulted in enrichments of only 97 DEGs (up/down ratio: 48:49) to 15 pathways (Figure 6(c), Table S6). The enriched KEGG pathways comprised 3–13 DEGs involved mainly in carbon/nitrogen/amino acid metabolisms, glycosphingolipid biosynthesis, peroxisome, glycan/glycosaminoglycan/fatty acid degradation, and MAPK signaling.

The validity of the transcriptomic data was clarified by transcript levels of 14 DEGs in the 3-day-old SDAY cultures of *dim5* mutants relative to the WT strain. As illustrated in Figure 6(d), transcript changes of those examined genes in the Δ*dim5* mutant coincided well with their anti-log_2_ *R*values and well restored by targeted *dim5* complementation into the mutant. Of those, four were involved in NCI (BBA_00049, BBA_00428, BBA_06599 and BBA_09825), and three involved in asexual development (BBA_04401, BBA_02876 and BBA_01528), in cell wall composition (BBA_07286, BBA_02996 and BBA_09822) or in transporting, transcriptional and translational activities (BBA_03202, BBA_09423 and BBA_07418).

The dysregulation of 1,201 genes in the absence of *dim5* highlights a genome-wide regulatory role of DIM5. The high up/down ratio of these genes suggests a link of their regulation to the conserved and noncanonical activities of DIM5 to multisite H3me due to an association of H3K9me with transcriptional repression and of H3K4me and H3K36me with actively transcribed genes in eukaryotes [2,3].

## *Transcriptomic insight into profound effect of* dim5 *on fungal lifecycle*

In the Δ*dim5* mutant, many dysregulated genes correlated with main phenotypic changes. The abolished pathogenicity via NCI was accompanied by malfunction of 37 genes encoding a variety of cuticle-degrading enzymes (Table S7). Markedly repressed genes included two adhesin-coding genes (BBA_02800 and BBA_06599) putatively involved in fungal attachment to insect cuticle and a novel hemolysin III family gene (MSTRG.10594) associated with microbial virulence [60]. Also listed in Table S7, dozens of dysregulated genes correlated with reduced tolerance to stress cues, such as those involved in responses to oxidative, cell wall perturbing, thermal and UV irradiating stresses. Particularly, up-regulated genes (partially shown in Figure 6(d)) coding for ConA-like lectin/glucanase (BBA_09822), cell wall glycoprotein (BBA_03412) and antigenic cell wall galactomannoprotein (BBA_02996) correlated well with increased carbohydrate epitopes on the lectin-labeled spore surfaces. Interestingly, none of the CDP genes required for conidiation appeared in the list of DEGs. Instead, three other development-mediated genes (Figure 6(d)) were significantly repressed, including both the clock gene *frq1* (BBA_01528) and the blue-light receptor gene *vvd* (BBA_02876) essential for the CDP activation and the asexual cycle *in vivo* and *in vitro* of *B. bassiana* [61–63] and another sporulation-specific gene (BBA_04401) functionally unknown yet. Moreover, 100 dysregulated genes (up/down ratio: 50:50) were putatively involved in transmembrane transport, cellular homeostasis and drug resistance, implicating their comprehensive effects on a wide array of cellular processes and biological aspects.

Notably, there were 92 dysregulated genes encoding transcription factors (TFs; up/down ratio: 35:15) and PTMs-required proteins/enzymes (up/down ratio: 24:18). All of them were involved in direct gene regulation. The up/down ratio (59:33) of such transcription-active genes approached to the up/down ratio (712:489) of all identified DEGs. These ratios hint again at that the genome-wide regulatory role of DIM5 could rely upon its conserved and noncanonical activities to H3K9me, H3K4me and H3K36me required for transcriptional repression and activation of those gene regulators in diverse pathways.

## Discussion

The KMT1, KMT2 and KMT3 families play conserved roles in histone H3K9me, H3K4me and H3K36me in eukaryotes respectively [5]. In *B. bassiana*, our study clarified not only a conserved role of DIM5/KMT1 in H3K9me, as reported previously in *S. pombe* [15] and some filamentous fungi [38,44,45], but also its noncanonical activities to H3K4me and H3K36me, which are usually mediated by SET1/KMT2 [3,20,49] and SET2/KMT3 [25–27] respectively. The three DIM5-methylated H3 lysines had profound effect on the fungal lifecycle *in vivo* and *in vitro*, as discussed below.

A nuclear localization of DIM5 makes it more robust to probe its activity to Lys-specific H3me in nuclear protein extracts than total protein extracts of hyphal cultures by western blotting. In our Δ*dim5* mutant, abolished H3K9me3 signal highlights a conserved activity of DIM5 to H3K9me3 in *B. bassiana* as well documented in eukaryotes [2–5]. Differentially attenuated signals of H3K4me1/me2 and H3K36me2 uncover its noncanonical roles in catalysis of H3K4me and H3K36me. Although involved mechanisms remain unclear, we speculate that the unusual activities of DIM5 could be associated with the absence of its C-terminal post-SET domain, which is critical for the activities of some enzymes targeting H3K4me, H3K9me and H3K36me [64–66]. However, this speculation remains to be clarified further by characterizing more DIM5 orthologues that also lack the C-terminal post-SET domain in Cordycipitaceae.

The mono-, di- and/or tri-methylation of the H3 core lysines attenuated or abolished in the absence of *dim5* implicate a pivotal role of DIM5 in *B. bassiana* that usually undergoes asexual cycle. This implication was clarified by its indispensable role in the fungal lifecycle *in vivo* and *in vitro*. Previously, plant pathogenicity was reduced, but not abolished, in the Δ*dim5* mutant of *B. cinerea* [44] or *F. verticillioides* [45]. In this study, the Δ*dim5* mutant lost the whole insect pathogenicity via NCI, and its virulence was also attenuated greatly via CBI. These indicate more profound effect of *dim5* disruption on the infectivity, virulence and associated cellular events of *B. bassiana* than of the plant pathogens and implicate a requirement of *dim5* for insect-pathogenic lifecycle. This implication was in further evidence with blocked biosynthesis and secretion of cuticle-degrading enzymes crucial for NCI [51,52], severe defects in stress tolerance required for collapse of host immune responses during NCI and hemocoel colonization [46,47], and a blockage of dimorphic transition to accelerate proliferation *in vivo*, host death, and hyphal outgrowth required for aerial conidiation on insect cadavers [63]. In *B. bassiana*, the key CDP activator genes *brlA* and *abaA* have been shown to act as master regulators of hyphal invasion into insect body, dimorphic transition and aerial conidiation [58]. Expression of both CDP genes was sharply repressed in the absence of *vvd* [61] or each of two genes encoding frequency proteins (Frq1 and Frq2), which have opposite rhythms in nucleus to persistently activate the CDP genes in daytime and nighttime and hence support nonrhythmic conidiation independent of photoperiod change [62]. In our Δ*dim5* mutant, abolished transcription of *brlA* and *abaA* at early developmental stage and marked repression of *vvd* and *frq1* in the transcriptome could be associated with the severe defects in aerial conidiation and submerged dimorphic transition *in vivo* and *in vitro*. Due to increased carbohydrate epitopes on the spore surfaces of Δ*dim5*, both injected conidia and subsequently formed hyphal bodies could have more PAMPs exposed to host recognition, leading to more intensive host immune responses to be collapsed by their longer interaction in the host hemocoel and hence disturbed cell cycle and delayed proliferation *in vivo* prior to the collapse. This inference helps to explain the Δ*dim5* virulence attenuated via CBI. Moreover, our transcriptome revealed dysregulation of 100 genes involved in membrane biogenesis, transmembrane transport, cellular homeostasis and drug resistance, which are collectively influential on a large array of cellular processes and biological traits. Particularly, the reduced tolerance of our Δ*dim5* mutant to multiple stress cues, including high osmolarity of sorbitol similar to that of concentrated trehalose in insect hemolymph, was different from increased Δ*dim5* osmotolerance in *F. verticillioides* [45]. Different and/or opposite phenotypes shown by the present and previous Δ*dim5* mutants suggest an evolutionary trajectory of DIM5 toward fungal adaptation to different hosts and associated habitats.

H3K9me associated with heterochromatin formation and gene silencing is somewhat distinct from H3K4me and H3K36me associated with actively transcribed genes [2–4,28]. In our Δ*dim5* mutant, inhibited H3K9me3 and attenuated H3K4me1/me2, H3K9me1/me2 and H3K36me2 collectively led to a high up/down ratio of dysregulated genes at genomic level. This suggests a dependence of genome stability on both conserved and noncanonical roles of DIM5 in methylating the H3 core lysines in *B. bassiana*. Previously, H3K4me and H3K36me were reported as marks of the nucleosomes associated with transcription-active genes [28]. H3K4me1/me2/me3 abolished in the absence of *kmt2*/*set1* resulted in nearly equal number of genes up- or down-regulated (770:728) in *M. robertsii* [49]. The up/down ratio (1.46) of dysregulated genes in the present study is far above the ratio (1.06) in the previous study, and closer to the up/down ratio (1.79) of 92 dysregulated genes encoding TFs and PTMs-required enzymes, which enable direct regulation of genome-wide gene expression. These ratios hint at that the DIM5-catalyzed H3K4me1/me2, H3K9me1/m2/me3 and H3K36me2 could act as preferential marks of those transcription-regulating genes.

In conclusion, DIM5 can methylate H3 lysines 9, 4 and 36 usually methylated by enzymes classified to the KMT1, KMT2 and KMT3 families respectively [5] and hence is essential for the lifecycle *in vivo* and *in vitro* and genome stability of *B. bassiana*. Our findings offer novel insight into both conserved and noncanonical activities of DIM5 to multisite H3me and its indispensability for the fungal insect-pathogenic lifecycle. Further studies are needed to clarify whether the DIM5-dependent multisite H3me occurs only in insect-pathogenic Cordycipitaceae.

## Materials and methods

### Comparative analysis of fungal DIM5/Clr4 orthologues

The amino acid sequences of *N. crassa* DIM5 (XP_957479) and *S. pombe* Clr4 (NP_595186) was used as queries of H3K9-specific KMT1 to search through the NCBI databases of selected ascomycetes including insect and noninsect pathogens. The main domains of the resultant DIM5/Clr4 orthologues were predicted at http://smart.embl-heidelberg.de/ and compared with those of the queries. The sequence feature of *B. bassiana* DIM5 lacking a predictable post-SET domain was further revealed with full results of conserved domain analysis at https://www.ncbi.nlm.nih.gov/Structure/. For all of examined DIM5/Clr4 orthologues, phylogenetic links were analyzed with a maximum likelihood method in MEGA7 software at http://www.megasoftware.net/.

## *Subcellular localization of DIM5 in* B. bassiana

Transgenic strains expressing the fusion gene *dim5::gfp* in the WT strain were generated on a basis of our backbone vector pAN52-C-gfp-bar, in which the C cassette 5′-*Pme*I-*Spe*I-*Eco*RV-*Eco*RI-*Bam*HI-3′ is under the control of P*tef1*, a promoter of homologous *tef1* encoding translation elongation factor 1 alpha [67]. Briefly, the coding sequence of *dim5* was amplified from the WT cDNA with paired primers (Table S2) and inserted into the vector digested with *Xma*I/*Bam*HI for its fusion to the N-terminus of *gfp* (GenBank ID: U55763), followed by integrating the new vector into the WT strain via *Agrobacterium*-mediated transformation. Putative transformants were screened by the *bar* resistance to phosphinothricin (200 μg/ml). A transformant expressing desirable green fluorescence signal was incubated for conidiation on SDAY (4% glucose, 1% peptone and 1.5% agar plus 1% yeast extract) at the optimal regime of 25°C and L:D 12:12. Conidia from the culture were incubated in SDBY for 3 days on a shaking bed (150 rpm) at 25°C. Hyphae collected from the culture were stained with the nuclear dye DAPI (4′,6′-diamidine-2′-phenylindole dihydrochloride; Sigma) and visualized for subcellular localization of the fusion protein through LSCM analysis. The intensities of expressed green fluorescence were measured from the nuclei and cytoplasm of cells in 10 hyphae by means of ImageJ software at https://imagej.nih.gov/ij/. The N/C-GFI ratios were computed as an index of relative accumulation levels of the fusion protein in the nuclei of those hyphae.

## *Generation of* dim5 *mutants*

Disruption of *dim5* in the WT strain was implemented by homologous recombination of its 5′ and 3′ coding/flanking fragments separated by the *bar* marker in the constructed vector p038-3′dim5-bar-5′dim5. Complementation of *dim5* into an identified Δ*dim5* mutant was achieved by ectopic integration of a cassette comprising its full-length coding sequence with flank regions and *sur* marker in the vector p0380-sur-dim5. The vectors were transformed into the corresponding strains as aforementioned. Putative mutant colonies were screened by the *bar* resistance to phosphinothricin (200 μg/ml) or the *sur* resistance to chlorimuron ethyl (10 μg/ml), followed by examination of expected recombinant events via PCR and Southern blot analyses. All of paired primers used for amplification of nucleotide sequences from the WT DNA and verification of the recombinant events are listed in Table S2. The identified Δ*dim5* and Δ*dim5::dim5* mutants (Fig. S2) were evaluated together with parental WT in the experiments with each comprising three independent replicates to meet a requirement for one-factor (strain) analysis of variance and Tukey’s honestly significant difference (HSD) test to differentiate means between the Δ*dim5* mutant and its control strains.

### Western blotting of lysine-specific H3me

A nuclear and cytoplasmic protein extraction kit (Beyotime, Shanghai, China; Catalog No.: P0027) was used to extract nuclear proteins from the 3-day-old cultures of 50 ml 10^6^ conidia/ml suspension in SDBY following the manufacturer’s guide. The concentration of each nuclear protein extract from the culture of each strain was assessed with BCA Protein Assay Kit (KeyGen Biotech, Nanjing, China). Aliquots of 40 μg protein extracts loaded onto 12% SDS-PAGE were transferred to polyvinylidene difluoride (PVDF) membranes (Merck Millipore, Darmstadt, Germany). Subsequently, signals of expressed H3 and mono-, di- and tri-methylated H3K4, H3K9 and H3K36 were probed with 1000-fold dilutions of appropriate antibodies (detailed in Table S3) in western blot experiments. The bound antibodies were reacted with 5000-fold dilution of horseradish peroxidase (HRP) conjugated AffiniPure Goat Anti-rabbit IgG (H + L) antibodies (Boster, Wuhan, China; Catalog No.: BA1054) and visualized in a chemiluminescence detection system (Amersham Biosciences, Shanghai, China). Each blotting experiment included three nuclear protein samples from independent cultures of each strain. Signal intensities of all blots were quantified with ImageJ. The level of each lysine-specific methylation relative to nuclear H3 accumulation was computed as a ratio of the corresponding intensities (H3K_me/H3 ratio).

### Bioassays for fungal insect pathogenicity and virulence

For each fungal strain, NCI was initiated by immersing three groups (replicates) of ~35* G. mellonella* larvae for 10 s in 40 ml aliquots of a 10^7^ conidia/ml suspension. CBI was initiated by injecting 5 μl of a 10^5^ conidia/ml suspension into the hemocoel of each larva in each group. A control for each bioassay consisted of three groups of larvae immersed in or injected with 0.02% Tween 80 used for preparation of conidial suspension. All groups of larvae treated per strain were maintained at 25°C for up to 11 days. During the period, survival/mortality records were taken every 12 h. LT_50_ (in days) indicative of virulence in either infection mode was estimated via probit analysis of the resultant time-mortality trend in each group.

### Assessments of virulence-related cellular events

A series of cellular events associated with NCI and subsequent hemocoel colonization were examined to gain an insight into abolished pathogenicity of the Δ*dim5* mutant via NCI and its attenuated virulence via CBI as described previously [63,67]. First, hyphal outgrowths and aerial conidiation on cadaver surfaces at 25°C after death from CBI were observed to reveal a capability of hyphal invasion into insect via the normal route of cuticular penetration. Second, 50 ml aliquots of a 10^6^ conidia/ml suspension in CDB (3% sucrose, 0.3% NaNO_3_, 0.1% K_2_HPO_4_, 0.05% KCl, 0.05% MgSO_4_ and 0.001% FeSO_4_) amended with the sole nitrogen source of 0.3% BSA were incubated on the shaking bed for 3 days at 25°C, followed by assessing biomass level of each culture and total extra- and intracellular activities of cuticle-degrading ECEs and Pr1 proteases as U/ml supernatant and U/mg protein extract isolated from the culture. Third, hemolymph samples taken from surviving larvae 72–120 h post-injection were examined under a microscopy to reveal a status of intrahaemocoel fungal proliferation crucial for a speed of host death, followed by assessing a concentration of hyphal bodies in each sample with a hemocytometer. Next, 50 ml aliquots of a 10^6^ conidia/ml suspension in TPB (CDB amended with 3% trehalose as sole carbon source and 0.5% peptone as sole nitrogen source) were incubated for 3 days on the shaking bed at 25°C. Blastospore concentration and biomass level (mg/ml) were quantified to compute dimorphic transition rate (no. blastospores/mg biomass) in the cultures of each strain. The conidia used for culture initiation and the resultant blastospores were stained with the DNA-specific dye propidium iodide or labeled with the Alexa Fluor 488-labeled lectins ConA, WGA, PNA and NGL (Vector Laboratories, Burlingame, CA, USA), respectively, followed by fluorescence-activated cell sorter (FACS) analysis to assess G1, G2 and S phases of cell cycle in 5 × 10^4^ stained blastospores and the contents of carbohydrate epitopes on the surfaces of 5 × 10^4^ labeled blastospores or conidia with an argon laser at the excitation/emission wavelengths of 488/530 (± 15) nm in the flow cytometer FC 500 MCL (Beckman Coulter, CA, USA).

To reveal effect of *dim5* disruption on hyphal growth and invasion into insect body, 1 μl aliquots of a 10^6^ conidia/ml suspension per strain were spotted on the plates of SDAY, CDA and CDAs amended with different carbon or nitrogen sources, followed by an 8-day incubation at 25°C and L:D 12:12. The diameter of each colony was assessed as a growth index using two measurements taken perpendicular to each other across the center. Further, hyphal responses to stress cues likely appearing in the courses of host infection and hemocoel colonization were assayed by initiating colonies as aforementioned on CDA alone (control) or supplemented with NaCl (0.7 M) or sorbitol (1 M) for osmotic stress, menadione (0.02 mM) or H_2_O_2_ (2 mM) for oxidative stress, and Congo red (3 μg/ml) or calcofluor white (10 μg/ml) for cell wall perturbing stress, respectively. The diameter of each colony was measured as above after an 8-day incubation at 25°C. For response to heat shock, 2-day-old SDAY colonies initiated at 25°C were exposed to a 42°C heat shock for 3–9 h, followed by a 6-day growth recovery at 25°C for measurements of colony diameters. Relative growth inhibition (RGI) percentage was calculated as an index of hyphal sensitivity to each stress (RGI = (*d*_c_–*d*_t_)/*d*_c_×100; *d*_c_, control colony diameter; *d*_t_, stressed colony diameter).

For insight into nearly invisible conidiation in the sparse Δ*dim5* outgrowths on cadaver surfaces, SDAY cultures initiated by spreading 100 μl of a 10^7^ conidia/ml suspension per plate (9 cm diameter) were incubated for 11 days at the optimal regime. Samples taken from the 3-day-old cultures were stained with the cell wall-specific dye calcofluor white and observed under a microscope to reveal conidiation status of each strain at the early stage of development. From day 5 onwards, conidial yield in three samples taken every 2 days from each plate culture with a cork borer (5 mm diameter) was measured as the number of conidia per unit area (cm^2^) of culture as described previously [61,62]. Meanwhile, biomass levels of each strain were measured from cellophane-overlaid SDAY cultures initiated with the same method. Next, several quality indices of conidia from the 8-day-old SDAY cultures were assessed due to potential impact of each on conidial infectivity and stress response, including assessments of GT_50_ (h) at 25°C, conidial hydrophobicity in an aqueous-organic system, LT_50_ (min) for conidial tolerance to a wet-heat stress at 45°C and LD_50_ (J/cm^2^) for conidial resistance to UVB irradiation (weighted wavelength: 312 nm), as described previously [54,56].

## *Transcription profiling of* dim5 *and CDP genes*

Cultures initiated by spreading conidia on cellophane-overlaid SDAY plates were incubated for 3 or 7 days at the optimal regime. Total RNAs were extracted from the WT cultures daily during the 7-day incubation or from the 3-day-old cultures of the *dim5* mutant and WT strains with an RNAiso Plus Kit (TaKaRa, Dalian, China), and reversely transcribed into cDNAs with a Prime ScriptHRT reagent kit (TaKaRa), respectively. Transcripts of *dim5* in the daily cDNA samples of the WT strain and of four CDP genes in the cDNA samples derived from the 3-day-old cultures were quantified via real-time quantitative PCR (qPCR) with paired primers (Table S2) under the action of SYBR Premix *Ex Taq* (TaKaRa). The fungal β-actin gene was used as a reference. A threshold-cycle (2^–ΔΔCT^) method was adopted to compute relative transcript levels of *dim5* in the daily WT cultures with respect to the standard level on day 2 and of each CDP gene in the *dim5* mutants with respect to the WT standard.

### Transcriptomic analysis

Three 3-day-old cultures (replicates) of Δ*dim5* and WT grown on cellophane-overlaid SDAY plates were prepared as aforementioned and sent to Weifen BioTech Co. (Hefei, Anhui, China) for construction of transcriptomes through the routine procedures of total RNA extraction, isolation of mRNA from total RNA, fragmentation of mRNA, and syntheses of first- and second-strand cDNAs. Purified double-stranded cDNAs were end-repaired by adding a single adenine to the end of each. The resultant cDNA library was sequenced on Illumina Novaseq 6000 platform, followed by normalization of all data as fragments per kilobase of exon per million fragments mapped (FPKM). Clean tags generated through filtration of all raw reads from the sequencing were mapped to the *B. bassiana* genome [50]. DEGs in Δ*dim5* versus WT were identified from the transcriptomes at the significant levels of both log_2_ *R*≤ –1 (down-regulated) or ≥ 1 (up-regulated) and FDR < 0.05. All DEGs were annotated with gene information in the non-redundant NCBI protein databases, and subjected to GO analysis (http://www.geneontology.org/) for enrichment to GO terms in the function classes (*p*< 0.05) of cellular component, molecular function and biological process and to KEGG analysis (http://www.genome.jp/kegg/) for enrichment to various pathways (*p*< 0.05).

The validity of transcriptomic data was examined by assessing relative transcript levels of 14 DEGs in the 3-day-old SDAY cultures of *dim5* mutants with respect to the WT standard through qPCR analysis with paired primers (Table S8), as aforementioned.

All transcriptomic data aside from those reported in Supplementary Material (Tables S4 to S7) of this paper are available at the NCBI’s Gene Expression Omnibus under the accession GSE164202 (https://www.ncbi.nlm.nih.gov/geo/query/acc.cgi?ac=GSE164202).

## Supplementary Material

Supplemental Material
