# Supplementary material for: DIM5/KMT1 controls fungal insect pathogenicity and genome stability by methylation of histone H3K4, H3K9 and H3K36
Source: Virulence. 2021 May 6;12(1):1306–22. doi: 10.1080/21505594.2021.1923232 (PMC8115510; doi:10.1080/21505594.2021.1923232)
Supplement: Supplemental Material [file KVIR_A_1923232_SM5188.docx]

Supplementary Material for Virulence

**DIM5/KMT1 controls fungal insect pathogenicity and genome stability by methylation of histone H3K4, H3K9 and H3K36**

Kang Ren, Ya-Ni Mou, Sen-Miao Tong, Sheng-Hua Ying, and Ming-Guang Feng *

(*Corresponding author's e-mail: mgfeng@zju.edu.cn)

**Contents:**

Fig. S1. Sequence analysis of fungal DIM5/Clr4 orthologues, page 2

Fig. S2. Generation and identification of *dim5* mutants in *B. bassiana*, page 3

Table S1. Full results from conserved domain analysis of *B. basssiana* DIM5, page 4

Table S2. Paired primers used for manipulation of target genes in *B. basssiana*, page 5

Table S3. Antibodies used for western blotting of histone H3 and mon-, di- and tri-methylated H3 lysines, page 5

Table S4. A list of differentially expressed genes identified from the transcriptome of Δ*dim5* versus WT, pages 6 to 25

Table S5. GO classification of differentially expressed genes in the transcriptome of Δ*dim5* versus WT, pages 26 to 28

Table S6. Enriched KEGG pathways of differentially expressed genes in the transcriptome of Δ*dim5* versus WT, page 28

Table S7. Differentially expressed genes associated with phenotypic changes and genome stability of Δ*dim5*, pages 29 to 33

Table S8. Paired primers used for qPCR analysis of 14 DEGs to validate transcriptomic data, page 33

**
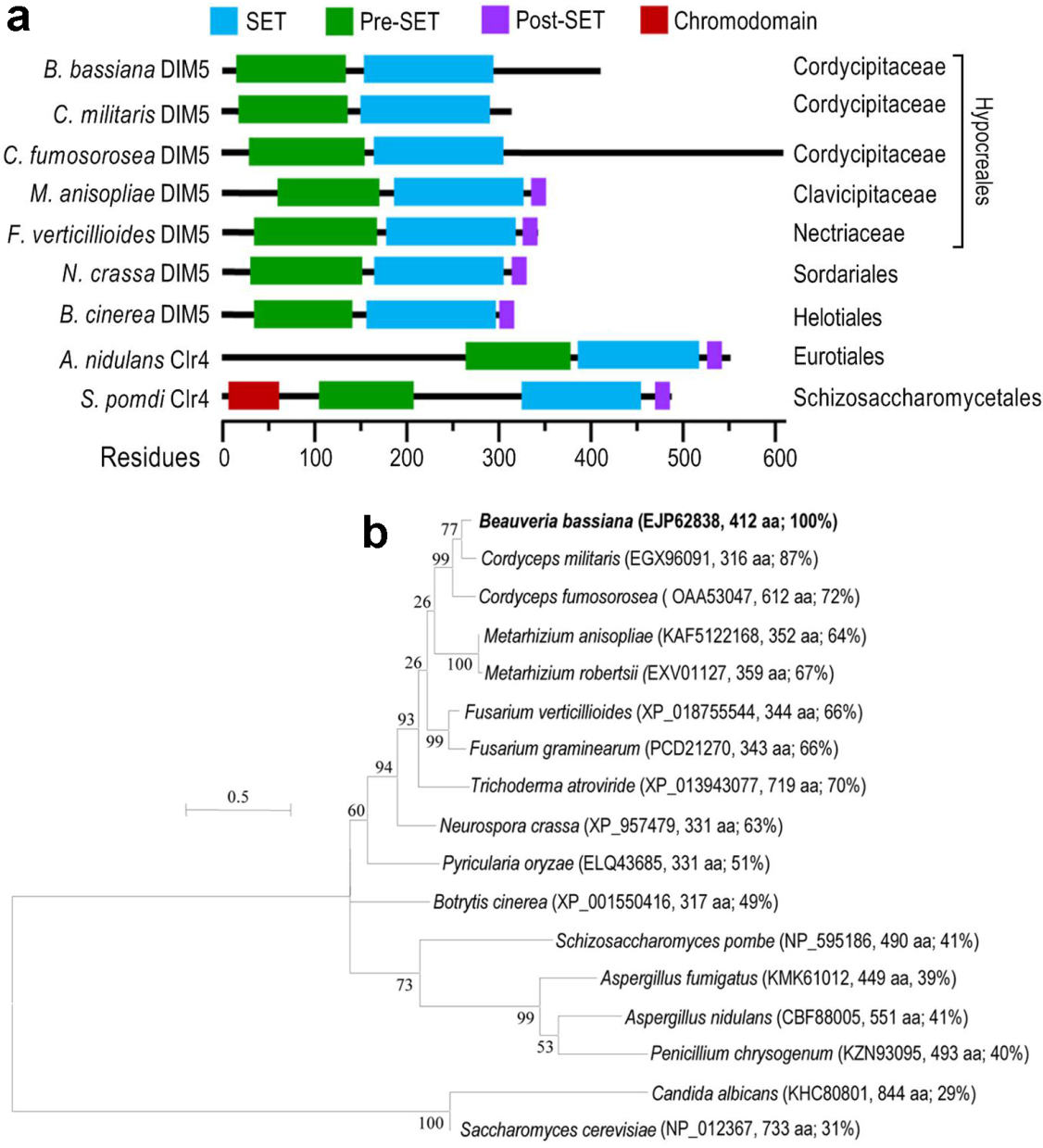
**

**Fig. S1.** Sequence analysis of fungal DIM5/Clr4 orthologues. (**a**) Comparison of main domains predicted via SMART domain analysis at <http://smart.embl-heidelberg.de/>. Note the absence of C-terminal post-SET domain in the orthologues of three fungi in Cordycipitaceae. (**b**) Phylogenetic links revealed with the maximum likelihood method in MEGA7 at <http://www.megasoftware.net/>. Bootstrap values of 1000 replications are shown at nodes. Scale: branch length proportional to genetic distance. The name of each fungus is followed by parentheses giving the NCBI accession code of its DIM5/Clr4, the length of its amino acid sequence, and its protein sequence identity to *B. bassiana* DIM5 respectively.


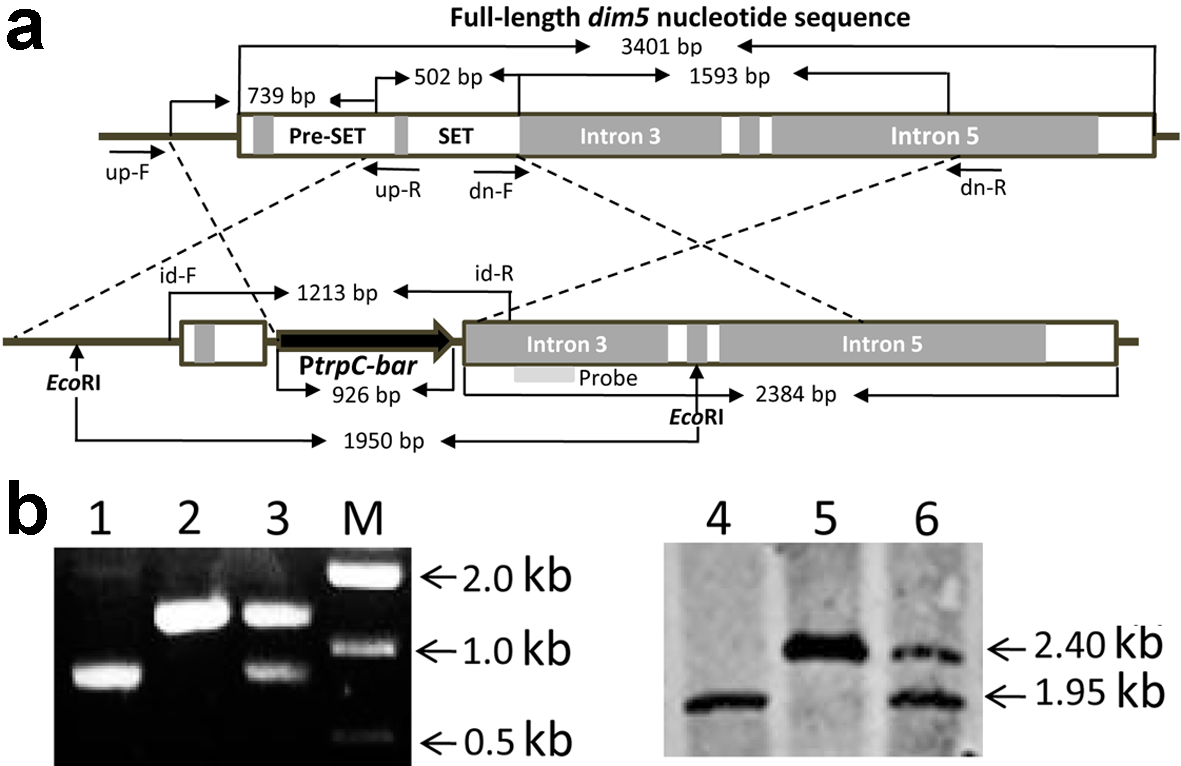


**Fig. S2.** Generation and identification of *dim5* mutants in *B. bassiana*. (**a**) Schematic diagram for the strategy of *dim5* disruption. Note that the full-length *dim5* nucleotide sequence of 3,401 bp consists of coding (white) and non-coding intron (grey) regions. (**b**) The *dim5* mutants identified through PCR (lanes 1–3) and Southern blot (lanes 4–6) analyses with paired primers and amplified 318-bp probe (Table S2) respectively. M, DNA ladder. Lanes 1 and 4: parental WT DNA. Lanes 2 and 5: Δ*dim5* DNA. Lanes 3 and 6: Δ*dim5::dim5* DNA. Genomic DNA of each strain used for Southern blotting of *dim5* was digested with *Eco*RI at the sites indicated in the diagram. Note that a 502-bp *dim5* fragment encoding the whole SET domain (residues 150-291) and partial pre-SET domain was deleted from the WT DNA by a replacement with P*trpC-bar*, as illustrated in (**a**), and that the deleted fragment was clarified by detected PCR bands of 789 and 1,213 bp in the DNAs of the WT and Δ*dim5* strains respectively and both bands in the DNA of Δ*dim5::dim5* (789 + 926 – 502 = 1213 bp).

**Table S1.** Full results from conserved domain analysis of *B. basssiana* DIM5.

| **Domain name** | **Accession** | **Description** | **Interval** | **E-value** |
| --- | --- | --- | --- | --- |
| SET_SUV39H_DIM5-like | [cd19473](https://www.ncbi.nlm.nih.gov/Structure/cdd/cddsrv.cgi?ascbin=8&maxaln=10&seltype=2&uid=cd19473) | SET domain (including pre-SET domain) found in *Neurospora crassa* (DIM-5) and similar proteins | 43-294 | 3.51E-163 |
| SET_SUV39H | [cd10542](https://www.ncbi.nlm.nih.gov/Structure/cdd/cddsrv.cgi?ascbin=8&maxaln=10&seltype=2&uid=cd10542) | SET domain (including pre-SET and post-SET domains) in suppressor of variegation 3-9 homologs, SUV39H1, SUV39H2, and similar proteins | 44-285 | 5.23E-87 |
| SET_SETDB-like | [cd10538](https://www.ncbi.nlm.nih.gov/Structure/cdd/cddsrv.cgi?ascbin=8&maxaln=10&seltype=2&uid=cd10538) | SET domain (including pre-SET and post-SET domains) found in SET domain bifurcated 1 (SETDB1) | 43-284 | 1.61E-79 |
| SET_SUV39H_Clr4-like | [cd20073](https://www.ncbi.nlm.nih.gov/Structure/cdd/cddsrv.cgi?ascbin=8&maxaln=10&seltype=2&uid=cd20073) | SET domain (including pre-SET and post-SET domains) found in *Schizosaccharomyces* pombe H3K9 | 44-312 | 2.78E-61 |
| SET_EHMT | [cd10543](https://www.ncbi.nlm.nih.gov/Structure/cdd/cddsrv.cgi?ascbin=8&maxaln=10&seltype=2&uid=cd10543) | SET domain (including pre-SET and post-SET domains) found in euchromatic histone-lysine | 67-287 | 8.98E-47 |
| SET_SETMAR | [cd10544](https://www.ncbi.nlm.nih.gov/Structure/cdd/cddsrv.cgi?ascbin=8&maxaln=10&seltype=2&uid=cd10544) | SET domain (including pre-SET and post-SET domains) found in SETMAR and similar proteins | 43-284 | 1.50E-45 |
| SET_AtSUVH-like | [cd10545](https://www.ncbi.nlm.nih.gov/Structure/cdd/cddsrv.cgi?ascbin=8&maxaln=10&seltype=2&uid=cd10545) | SET domain found in *Arabidopsis thalian*a histone H3-K9 methyltransferases (SUVHs) | 45-284 | 1.23E-42 |
| SET_SUV39H2 | [cd10532](https://www.ncbi.nlm.nih.gov/Structure/cdd/cddsrv.cgi?ascbin=8&maxaln=10&seltype=2&uid=cd10532) | SET domain (including pre-SET and post-SET domains) found in SUV39H2 and similar proteins | 44-284 | 1.49E-40 |
| SET_SUV39H1 | [cd10525](https://www.ncbi.nlm.nih.gov/Structure/cdd/cddsrv.cgi?ascbin=8&maxaln=10&seltype=2&uid=cd10525) | SET domain (including pre-SET and post-SET domains) found in SUV39H1 and similar proteins | 43-298 | 3.46E-40 |
| SET_SETDB1 | [cd10517](https://www.ncbi.nlm.nih.gov/Structure/cdd/cddsrv.cgi?ascbin=8&maxaln=10&seltype=2&uid=cd10517) | SET domain (including pre-SET and post-SET domains) found in SETDB1 and similar proteins | 33-284 | 5.43E-37 |
| SET_EHMT2 | [cd10533](https://www.ncbi.nlm.nih.gov/Structure/cdd/cddsrv.cgi?ascbin=8&maxaln=10&seltype=2&uid=cd10533) | SET domain (including pre-SET and post-SET domains) found in EHMT2 and similar proteins | 68-286 | 5.86E-34 |
| SET_EHMT1 | [cd10535](https://www.ncbi.nlm.nih.gov/Structure/cdd/cddsrv.cgi?ascbin=8&maxaln=10&seltype=2&uid=cd10535) | SET domain (including pre-SET and post-SET domains) found in EHMT1 and similar proteins | 68-286 | 4.58E-33 |
| SET | [smart00317](https://www.ncbi.nlm.nih.gov/Structure/cdd/cddsrv.cgi?ascbin=8&maxaln=10&seltype=2&uid=smart00317) | SET (Su(var)3-9, Enhancer-of-zeste, Trithorax) domain | 150-284 | 6.21E-30 |
| SET_ASH1L | [cd19174](https://www.ncbi.nlm.nih.gov/Structure/cdd/cddsrv.cgi?ascbin=8&maxaln=10&seltype=2&uid=cd19174) | SET domain (including post-SET domain) found in ASH1-like protein (ASH1L) and similar proteins | 152-284 | 1.71E-28 |
| SET_NSD | [cd19173](https://www.ncbi.nlm.nih.gov/Structure/cdd/cddsrv.cgi?ascbin=8&maxaln=10&seltype=2&uid=cd19173) | SET domain (including post-SET domain) found in nuclear SET domain-containing proteins | 150-284 | 1.07E-26 |
| SET_SETD2-like | [cd10531](https://www.ncbi.nlm.nih.gov/Structure/cdd/cddsrv.cgi?ascbin=8&maxaln=10&seltype=2&uid=cd10531) | SET domain (including post-SET domain) found in SET domain-containing protein 2 (SETD2) | 151-284 | 2.95E-26 |
| SET | [pfam00856](https://www.ncbi.nlm.nih.gov/Structure/cdd/cddsrv.cgi?ascbin=8&maxaln=10&seltype=2&uid=pfam00856) | SET domain found in lysine methyltransferase enzymes | 161-284 | 7.26E-23 |
| SET_SETDB | [cd10541](https://www.ncbi.nlm.nih.gov/Structure/cdd/cddsrv.cgi?ascbin=8&maxaln=10&seltype=2&uid=cd10541) | SET domain (including pre-SET and post-SET domains) found in SETDB1, SETDB2, and similar proteins | 43-284 | 1.77E-22 |
| SET_SETD1-like | [cd10518](https://www.ncbi.nlm.nih.gov/Structure/cdd/cddsrv.cgi?ascbin=8&maxaln=10&seltype=2&uid=cd10518) | SET domain (including post-SET domain) found in SET domain-containing proteins (SETD1A/SETD1B) | 161-284 | 2.18E-21 |
| SET_EZH | [cd10519](https://www.ncbi.nlm.nih.gov/Structure/cdd/cddsrv.cgi?ascbin=8&maxaln=10&seltype=2&uid=cd10519) | SET domain found in enhancer of zeste homolog 1 (EZH1), zeste homolog 2 (EZH2) and similar proteins | 161-284 | 1.98E-20 |
| SET_SETDB2 | [cd10523](https://www.ncbi.nlm.nih.gov/Structure/cdd/cddsrv.cgi?ascbin=8&maxaln=10&seltype=2&uid=cd10523) | SET domain (including pre-SET and post-SET domains) found in SETDB2 and similar proteins | 62-284 | 2.75E-20 |
| SET_NSD1 | [cd19210](https://www.ncbi.nlm.nih.gov/Structure/cdd/cddsrv.cgi?ascbin=8&maxaln=10&seltype=2&uid=cd19210) | SET domain (including post-SET domain) found in NSD1 and similar proteins | 152-284 | 1.58E-19 |
| SET_SETD2 | [cd19172](https://www.ncbi.nlm.nih.gov/Structure/cdd/cddsrv.cgi?ascbin=8&maxaln=10&seltype=2&uid=cd19172) | SET domain (including post-SET domain) found in SETD2 and similar proteins | 151-284 | 1.10E-18 |
| Pre-SET | [pfam05033](https://www.ncbi.nlm.nih.gov/Structure/cdd/cddsrv.cgi?ascbin=8&maxaln=10&seltype=2&uid=pfam05033) | Pre-SET motif as a zinc binding motif containing 9 conserved cysteines | 31-142 | 1.34E-18 |
| SET | [COG2940](https://www.ncbi.nlm.nih.gov/Structure/cdd/cddsrv.cgi?ascbin=8&maxaln=10&seltype=2&uid=COG2940) | SET domain-containing protein (function unknown) | 121-295 | 8.31E-18 |
| SET_NSD3 | [cd19212](https://www.ncbi.nlm.nih.gov/Structure/cdd/cddsrv.cgi?ascbin=8&maxaln=10&seltype=2&uid=cd19212) | SET domain (including post-SET domain) found in NSD3 and similar proteins | 153-284 | 1.18E-17 |
| SET_NSD2 | [cd19211](https://www.ncbi.nlm.nih.gov/Structure/cdd/cddsrv.cgi?ascbin=8&maxaln=10&seltype=2&uid=cd19211) | SET domain (including post-SET domain) found in NSD2 and similar proteins | 153-284 | 2.27E-17 |
| SET_ASHR3-like | [cd19175](https://www.ncbi.nlm.nih.gov/Structure/cdd/cddsrv.cgi?ascbin=8&maxaln=10&seltype=2&uid=cd19175) | SET domain (including post-SET domain) found in ASHR3 and similar proteins | 152-284 | 5.56E-16 |
| SET_KMT2C_2D | [cd19171](https://www.ncbi.nlm.nih.gov/Structure/cdd/cddsrv.cgi?ascbin=8&maxaln=10&seltype=2&uid=cd19171) | SET domain (including post-SET domain) found in KMT2C, KMT2D and similar proteins | 156-284 | 1.47E-15 |
| SET_LegAS4-like | [cd10522](https://www.ncbi.nlm.nih.gov/Structure/cdd/cddsrv.cgi?ascbin=8&maxaln=10&seltype=2&uid=cd10522) | SET domain found in *Legionella pneumophila* type IV secretion system effector LegAS4 and similar proteins | 161-284 | 5.73E-14 |
| SET | [cd08161](https://www.ncbi.nlm.nih.gov/Structure/cdd/cddsrv.cgi?ascbin=8&maxaln=10&seltype=2&uid=cd08161) | SET (Su(var)3-9, Enhancer-of-zeste, Trithorax) domain superfamily | 231-284 | 1.86E-13 |
| SET_EZH2 | [cd19218](https://www.ncbi.nlm.nih.gov/Structure/cdd/cddsrv.cgi?ascbin=8&maxaln=10&seltype=2&uid=cd19218) | SET domain found in enhancer of zeste homolog 2 (EZH2) and similar proteins | 161-284 | 7.05E-13 |
| SET_SET1 | [cd20072](https://www.ncbi.nlm.nih.gov/Structure/cdd/cddsrv.cgi?ascbin=8&maxaln=10&seltype=2&uid=cd20072) | SET domain (including post-SET domain) found in catalytic component of yeast COMPASS and similar proteins | 146-284 | 9.56E-13 |
| SET_SETD8 | [cd10528](https://www.ncbi.nlm.nih.gov/Structure/cdd/cddsrv.cgi?ascbin=8&maxaln=10&seltype=2&uid=cd10528) | SET domain found in SET domain-containing protein 8 (SETD8) and similar proteins | 142-286 | 1.27E-12 |
| SET_KMT2A_2B | [cd19170](https://www.ncbi.nlm.nih.gov/Structure/cdd/cddsrv.cgi?ascbin=8&maxaln=10&seltype=2&uid=cd19170) | SET domain (including post-SET domain) found in KMT2A, KMT2B and similar proteins | 154-284 | 9.14E-12 |
| SET_KMT2A | [cd19206](https://www.ncbi.nlm.nih.gov/Structure/cdd/cddsrv.cgi?ascbin=8&maxaln=10&seltype=2&uid=cd19206) | SET domain (including post-SET domain) found in KMT2A and similar proteins | 154-303 | 1.89E-11 |
| SET_EZH-like | [cd19168](https://www.ncbi.nlm.nih.gov/Structure/cdd/cddsrv.cgi?ascbin=8&maxaln=10&seltype=2&uid=cd19168) | SET domain found in EZH1 and EZH2 of polycomb repressive complex 2 (PRC2), and similar proteins | 161-286 | 9.86E-11 |
| SET_KMT2D | [cd19209](https://www.ncbi.nlm.nih.gov/Structure/cdd/cddsrv.cgi?ascbin=8&maxaln=10&seltype=2&uid=cd19209) | SET domain (including post-SET domain) found in KMT2D and similar proteins | 156-306 | 2.42E-10 |
| SET_SpSET3-like | [cd19183](https://www.ncbi.nlm.nih.gov/Structure/cdd/cddsrv.cgi?ascbin=8&maxaln=10&seltype=2&uid=cd19183) | SET domain (including post-SET domain) found in *Schizosaccharomyces pombe* SETD3 and similar proteins | 150-284 | 2.95E-10 |
| SET_EZH1 | [cd19217](https://www.ncbi.nlm.nih.gov/Structure/cdd/cddsrv.cgi?ascbin=8&maxaln=10&seltype=2&uid=cd19217) | SET domain found in enhancer of zeste homolog 1 (EZH1) and similar proteins | 145-284 | 1.07E-09 |
| SET_SETD5-like | [cd10529](https://www.ncbi.nlm.nih.gov/Structure/cdd/cddsrv.cgi?ascbin=8&maxaln=10&seltype=2&uid=cd10529) | SET domain found in SETD5, KMT2E and similar proteins | 163-284 | 2.38E-09 |
| SET_KMT2B | [cd19207](https://www.ncbi.nlm.nih.gov/Structure/cdd/cddsrv.cgi?ascbin=8&maxaln=10&seltype=2&uid=cd19207) | SET domain (including post-SET domain) found in KMT2B and similar proteins | 154-303 | 4.09E-09 |
| SET_KMT2C | [cd19208](https://www.ncbi.nlm.nih.gov/Structure/cdd/cddsrv.cgi?ascbin=8&maxaln=10&seltype=2&uid=cd19208) | SET domain (including post-SET domain) found in KMT2C and similar proteins | 156-306 | 7.77E-09 |
| SET_SETD1 | [cd19169](https://www.ncbi.nlm.nih.gov/Structure/cdd/cddsrv.cgi?ascbin=8&maxaln=10&seltype=2&uid=cd19169) | SET domain (including post-SET domain) found in SETD1 and similar proteins | 161-284 | 9.55E-09 |
| SET_SMYD | [cd20071](https://www.ncbi.nlm.nih.gov/Structure/cdd/cddsrv.cgi?ascbin=8&maxaln=10&seltype=2&uid=cd20071) | SET domain (including SET domain and post-SET domain) in SET and MYND domain-containing and similar proteins | 240-295 | 8.11E-08 |
| SET_SMYD3 | [cd19203](https://www.ncbi.nlm.nih.gov/Structure/cdd/cddsrv.cgi?ascbin=8&maxaln=10&seltype=2&uid=cd19203) | SET domain (including post-SET domain) found in SMYD3 and similar proteins | 152-295 | 2.91E-06 |
| SET_SETD1A | [cd19204](https://www.ncbi.nlm.nih.gov/Structure/cdd/cddsrv.cgi?ascbin=8&maxaln=10&seltype=2&uid=cd19204) | SET domain (including post-SET domain) found in SETD1A and similar proteins | 148-284 | 4.04E-06 |
| SET_Suv4-20-like | [cd10524](https://www.ncbi.nlm.nih.gov/Structure/cdd/cddsrv.cgi?ascbin=8&maxaln=10&seltype=2&uid=cd10524) | SET domain (including post-SET domain) found in *Drosophila melanogaster* Suv4-20 and similar proteins | 233-287 | 6.22E-06 |
| SET_SETD1B | [cd19205](https://www.ncbi.nlm.nih.gov/Structure/cdd/cddsrv.cgi?ascbin=8&maxaln=10&seltype=2&uid=cd19205) | SET domain (including post-SET domain) found in SETD1B and similar proteins | 148-306 | 1.73E-05 |
| SET_SpSet7-like | [cd10540](https://www.ncbi.nlm.nih.gov/Structure/cdd/cddsrv.cgi?ascbin=8&maxaln=10&seltype=2&uid=cd10540) | SET domain found in *Schizossacharomyces pombe* Set7 and similar proteins | 151-284 | 2.55E-05 |
| SET_KMT5C | [cd19185](https://www.ncbi.nlm.nih.gov/Structure/cdd/cddsrv.cgi?ascbin=8&maxaln=10&seltype=2&uid=cd19185) | SET domain (including post-SET domain) found in KMT5C and similar proteins | 236-295 | 3.74E-05 |
| SET_SMYD4 | [cd10536](https://www.ncbi.nlm.nih.gov/Structure/cdd/cddsrv.cgi?ascbin=8&maxaln=10&seltype=2&uid=cd10536) | SET domain (including iSET domain and post-SET domain) found in SMYD4 and similar proteins | 240-284 | 1.24E-04 |
| SET_SETD5 | [cd19181](https://www.ncbi.nlm.nih.gov/Structure/cdd/cddsrv.cgi?ascbin=8&maxaln=10&seltype=2&uid=cd19181) | SET domain (including post-SET domain) found in SETD5 and similar proteins | 220-284 | 2.26E-04 |
| PreSET | [smart00468](https://www.ncbi.nlm.nih.gov/Structure/cdd/cddsrv.cgi?ascbin=8&maxaln=10&seltype=2&uid=smart00468) | N-terminal to some SET domains | 28-84 | 1.03E-03 |
| SET_KMT5B | [cd19184](https://www.ncbi.nlm.nih.gov/Structure/cdd/cddsrv.cgi?ascbin=8&maxaln=10&seltype=2&uid=cd19184) | SET domain (including post-SET domain) found in KMT5B and similar proteins | 236-294 | 4.87E-03 |

**Table S2.** Paired primers used for manipulation of target genes in *B. basssiana*.

| Primers | Paired sequences (5′−3′) ^a^ | Purpose ^b^ |
| --- | --- | --- |
| Dim5-F/R | CAATCACAAACACCTTCAAAATGGAGCAAGCTATTGAGGAGC / CTCGCCCTTGCTCA CCATCCTCTTTCCGTTCTCGTAGAATTGAATG | Cloning *dim5* cDNA (1236 bp) for fusion to *gfp* |
| upDim5-F/R | ACGAGCTGTACAAGTAACCCGGGGTAGGTACTTTTTCCTTA / TGGCTGCAGGTCGA CGGATCCACCTCGCTCGACAACTCT | Cloning 3′ *dim5* (739 bp) for targeted gene disruption |
| dnDim5-F/R | GACCCATGGCTCGAGTCTAGAGTGGAGGGTATGACGAAATG / GGTGGTGGTGGCT AGCGTTAACTGGGAGGAGTGGTAAAATAA | Cloning 5′ *dim5* (1593 bp) for targeted gene disruption |
| flDim5-F/R | ATCCGTCGACCTGCAGCCAAGCTTAATTAAGGTCTTAGGGA / ACACTAGTCAGATCT TCTAGTGTTTTGGGACTGTGCTTCTGGA | Cloning full-length *dim5* (4491 bp) for complementation |
| pDim5-F/R | GCCCCTGTATGAATGCCA / GTAGCCTTGGTTTCCCCG | PCR detecting *dim5* |
| sbDim5-F/R | GGTGAGGCAAACAAAGGA / CCGAGGCTCTGGTGAACT | Cloning Southern probe |
| qDim5-F/R | ATGCCCGAATAGAGTTGTCG / TTGAGACACTGCCGAGTTTG | qPCR detecting *dim5* |
| qBrlA-F/R | GATGGATGACAAGTGCGATG / AAACTCGCACGAGAAACGAT | qPCR detecting *brlA* |
| qAbaA-F/R | GCAAGTCTCCAGCCATAT / CTCCTCTTCGTCATAGTAGTC | qPCR detecting *abaA* |
| qWetA-F/R | ATGCGGTACTACAGCCAAGG / GAGTTCCTGCTGGCTACTGG | qPCR detecting *wetA* |
| qVosA-F/R | ACTCATGGGCTCATTGGTGG / CCGGCAAGAGAGATCCGAAA | qPCR detecting *vosA* |
| qAct-F/R | GGCAACATTGTCATGTCTGG / TTTGCTGGAAGGTGGATAGG | qPCR detecting β-actin gene |

^a^ Underlined regions denote introduced cleavage sites of two pairs of restriction enzymes for homologous recombination of the *bar*-separated 5′ and 3′ fragments of *dim5* (*Xma*I/*Bam*HI *and Xba*I/*Hpa*I) for targeted gene disruption or the recognition fragments for gateway exchange to construct the complementation vector.

^b^ PCR detection aimed at the fragments of 1213 bp for Δ*dim5* and 789 bp for WT. The probe amplified for Southern blotting of *dim5* is 318 bp in length. Tag loci for *brlA*, *abaA*, *wetA*, *vosA* and the β-actin coding gene are BBA_07544, BBA_00300, BBA_06126, BBA_01023 and BBA_04860, respectively, under the NCBI accession NZ_ADAH00000000 of *B. bassiana* genome

**Table S3.** Antibodies used for Western blotting of histone H3 and mon-, di- and tri-methylated H3 lysines.

| Name of antibody product | No. catalog | Manufacturer* | Purpose |
| --- | --- | --- | --- |
| [Histone H3 (D1H2) XP^®^ Rabbit mAb](https://www.cellsignal.cn/products/primary-antibodies/histone-h3-d1h2-xp-rabbit-mab/4499?N=4294956287&Ntt=4499s&fromPage=plp) | 4499S | CST | Anti-histone H3 |
| [Mono-Methyl-Histone H3 (Lys4) (D1A9) XP^®^ Rabbit mAb](https://www.cellsignal.cn/products/primary-antibodies/mono-methyl-histone-h3-lys4-d1a9-xp-rabbit-mab/5326?N=4294956287&Ntt=5326t&fromPage=plp) | 5326T | CST | Anti-H3K4me1 |
| Di-Methyl-Histone H3 (Lys4) (C64G9) Rabbit mAb | 9725T | CST | Anti-H3K4me2 |
| [Tri-Methyl-Histone H3 (Lys4) (C42D8) Rabbit mAb](https://www.cellsignal.cn/products/primary-antibodies/tri-methyl-histone-h3-lys4-c42d8-rabbit-mab/9751?N=4294956287&Ntt=9751s&fromPage=plp) | 9751S | CST | Anti-H3K4me3 |
| [Mono-Methyl-Histone H3 (Lys9) Rabbit Polyclonal](https://www.cellsignal.cn/products/primary-antibodies/mono-methyl-histone-h3-lys4-d1a9-xp-rabbit-mab/5326?N=4294956287&Ntt=5326t&fromPage=plp) | 39887 | Active Motif | Anti-H3K9me1 |
| [Di-Methyl-Histone H3 (Lys9) (D85B4) XP^®^ Rabbit mAb](https://www.cellsignal.cn/products/primary-antibodies/di-methyl-histone-h3-lys9-d85b4-xp-rabbit-mab/4658?N=4294956287&Ntt=4658t&fromPage=plp) | 4658T | CST | Anti-H3K9me2 |
| [Tri-Methyl-Histone H3 (Lys9) (D4W1U) Rabbit mAb](https://www.cellsignal.cn/products/primary-antibodies/tri-methyl-histone-h3-lys9-d4w1u-rabbit-mab/13969?N=4294956287&Ntt=13969s&fromPage=plp) | 13969S | CST | Anti-H3K9me3 |
| Anti-Histone H3 (mono methyl K36) Rabbit Polyclonal | ab9048 | Abcam | Anti-H3K36me1 |
| [Di-Methyl-Histone H3 (Lys36) (C75H12) Rabbit mAb](https://www.cellsignal.cn/products/primary-antibodies/di-methyl-histone-h3-lys36-c75h12-rabbit-mab/2901?N=4294956287&Ntt=2901t&fromPage=plp) | 2901T | CST | Anti-H3K36me2 |
| [Tri-Methyl-Histone H3 (Lys36) (D5A7) XP^®^ Rabbit mAb](https://www.cellsignal.cn/products/primary-antibodies/tri-methyl-histone-h3-lys36-d5a7-xp-rabbit-mab/4909?N=4294956287&Ntt=4909s&fromPage=plp) | 4909S | CST | Anti-H3K36me3 |

* CST, Cell Signaling Technology (Boston, MA, USA); Active Motif (Shanghai, China); Abcam (Shanghai, China).

**Table S4.** A list of differentially expressed genes identified from the transcriptome of Δ*dim5* versus WT.

| **Gene_ID** | **Transcript_ID** | **log_2_ *R*** | **FDR** | **Regulation** | **NR_Annotation** |
| --- | --- | --- | --- | --- | --- |
| BBA_02837 | XM_008597934.1 | -8.970 | 1.35E-19 | down | ADP-ribosylation factor |
| BBA_03917 | XM_008599014.1 | -6.119 | 4.16E-05 | down | putative endochitinase CHI3 |
| MSTRG.13915 | MSTRG.13915.1 | -5.536 | 1.61E-03 | down | GTP cyclohydrolase I |
| BBA_01574 | XM_008596671.1 | -5.503 | 1.84E-30 | down | phosphate permease |
| BBA_07294 | XM_008602391.1 | -5.387 | 2.54E-03 | down | ribosomal protein L7Ae |
| BBA_03753 | XM_008598850.1 | -5.278 | 6.07E-03 | down | FMN-dependent dehydrogenase |
| BBA_03678 | XM_008598775.1 | -4.929 | 2.34E-02 | down | hypothetical protein BBA_04607 |
| BBA_08040 | XM_008603137.1 | -4.794 | 3.71E-02 | down | HemK family methyltransferase |
| BBA_02397 | XM_008597494.1 | -4.766 | 3.71E-02 | down | -- |
| BBA_04607 | XM_008599704.1 | -4.593 | 3.07E-19 | down | Acyl-CoA N-acyltransferase |
| BBA_02153 | XM_008597250.1 | -4.565 | 1.65E-21 | down | TfdA family Taurine catabolism dioxygenase TauD |
| MSTRG.10392 | MSTRG.10392.1 | -4.480 | 3.06E-20 | down | cupin domain protein |
| BBA_02926 | XM_008598023.1 | -4.470 | 2.24E-16 | down | glycosyltransferase family 4 |
| BBA_05761 | XM_008600858.1 | -4.254 | 1.28E-19 | down | phospholipase C PLC-C |
| BBA_08661 | XM_008603758.1 | -4.140 | 2.82E-19 | down | phosphoesterase-like protein |
| MSTRG.2896 | MSTRG.2896.1 | -4.113 | 6.82E-18 | down | acyl-CoA dehydrogenase domain-containing protein |
| MSTRG.9332 | MSTRG.9332.1 | -4.112 | 3.98E-19 | down | acid phosphatase |
| BBA_05762 | XM_008600859.1 | -3.989 | 1.55E-17 | down | Phosphoesterase-like protein |
| BBA_07651 | XM_008602748.1 | -3.970 | 3.66E-17 | down | hypothetical protein BBA_10187 |
| BBA_10187 | XM_008605284.1 | -3.939 | 9.42E-11 | down | urea active transporter |
| BBA_07917 | XM_008603014.1 | -3.836 | 8.30E-10 | down | Major facilitator superfamily, general substrate transporter |
| MSTRG.8559 | MSTRG.8559.1 | -3.815 | 4.66E-10 | down | hypothetical protein BBO_09316 |
| BBA_03202 | XM_008598299.1 | -3.808 | 2.42E-14 | down | transporter-like protein |
| MSTRG.10866 | MSTRG.10866.1 | -3.794 | 4.32E-03 | down | ribose-phosphate pyrophosphokinase, putative |
| BBA_00689 | XM_008595786.1 | -3.737 | 2.22E-09 | down | hypothetical protein BBAD15_g2733 |
| BBA_05007 | XM_008600104.1 | -3.703 | 1.02E-14 | down | methionine permease |
| BBA_05802 | XM_008600899.1 | -3.620 | 5.28E-04 | down | hypothetical protein BBA_05405 |
| BBA_08405 | XM_008603502.1 | -3.480 | 2.72E-06 | down | S25 ribosomal protein |
| BBA_03463 | XM_008598560.1 | -3.477 | 4.18E-11 | down | Protein of unknown function DUF3605 |
| BBA_06571 | XM_008601668.1 | -3.368 | 9.45E-13 | down | hypothetical protein BBA_02122 |
| BBA_08573 | XM_008603670.1 | -3.308 | 5.23E-07 | down | glycosyl transferase |
| BBA_06796 | XM_008601893.1 | -3.288 | 3.85E-03 | down | hypothetical protein BBA_06157 |
| BBA_04241 | XM_008599338.1 | -3.241 | 3.55E-13 | down | hypothetical protein BBA_00689 |
| MSTRG.14122 | MSTRG.14122.1 | -3.219 | 3.16E-10 | down | glycerophosphoryl diester phosphodiesterase |
| BBA_02122 | XM_008597219.1 | -3.172 | 1.65E-09 | down | -- |
| BBA_01995 | XM_008597092.1 | -3.096 | 5.01E-05 | down | FAD dependent oxidoreductase |
| BBA_05768 | XM_008600865.1 | -3.079 | 2.36E-03 | down | GARP complex component (Vps54) |
| MSTRG.4649 | MSTRG.4649.1 | -3.011 | 3.17E-07 | down | TfdA family Taurine catabolism dioxygenase TauD |
| BBA_00688 | XM_008595785.1 | -2.992 | 4.36E-03 | down | UbiA prenyltransferase |
| BBA_05508 | XM_008600605.1 | -2.943 | 6.62E-09 | down | hypothetical protein BBA_00687 |
| BBA_07281 | XM_008602378.1 | -2.928 | 1.09E-10 | down | TfdA family Taurine catabolism dioxygenase TauD |
| BBA_07286 | XM_008602383.1 | -2.926 | 4.18E-11 | down | hydrophobin-like protein |
| BBA_05764 | XM_008600861.1 | -2.863 | 1.18E-10 | down | hypothetical protein BBA_00968 |
| MSTRG.13959 | MSTRG.13959.1 | -2.854 | 6.47E-05 | down | C6 transcription factor |
| MSTRG.12658 | MSTRG.12658.1 | -2.827 | 4.53E-10 | down | hisactophilin C49S mutant/phototropin PHY3 fusion protein |
| BBA_09771 | XM_008604868.1 | -2.822 | 8.50E-09 | down | 2-C-methyl-D-erythritol 2,4-cyclodiphosphate synthase |
| BBA_07717 | XM_008602814.1 | -2.798 | 5.31E-04 | down | DJ-1/PfpI family protein |
| BBA_00049 | XM_008595146.1 | -2.789 | 4.12E-10 | down | intracellular serine protease |
| BBA_03404 | XM_008598501.1 | -2.785 | 1.11E-02 | down | hypothetical protein BBA_06750 |
| BBA_01994 | XM_008597091.1 | -2.783 | 5.89E-10 | down | ribonuclease T2 family protein |
| MSTRG.3571 | MSTRG.3571.1 | -2.775 | 1.07E-08 | down | LPS glycosyltransferase, putative |
| BBA_08576 | XM_008603673.1 | -2.757 | 7.77E-10 | down | FAD binding domain-containing protein |
| BBA_05405 | XM_008600502.1 | -2.756 | 7.18E-10 | down | hypothetical protein BBA_06442 |
| BBA_10301 | XM_008605398.1 | -2.751 | 1.64E-08 | down | major facilitator superfamily transporter |
| BBA_09445 | XM_008604542.1 | -2.743 | 4.64E-03 | down | extracellular serine-rich protein |
| BBA_01424 | XM_008596521.1 | -2.738 | 4.64E-03 | down | aminotransferase class-III |
| BBA_01784 | XM_008596881.1 | -2.691 | 1.86E-09 | down | hypothetical protein BBA_06442 |
| BBA_06157 | XM_008601254.1 | -2.656 | 2.15E-09 | down | serine carboxypeptidase |
| BBA_06418 | XM_008601515.1 | -2.646 | 4.27E-04 | down | -- |
| BBA_00417 | XM_008595514.1 | -2.644 | 2.54E-04 | down | hypothetical protein BBA_05273 |
| BBA_09496 | XM_008604593.1 | -2.637 | 4.33E-09 | down | peptidase S1 and S6, chymotrypsin/Hap |
| BBA_07943 | XM_008603040.1 | -2.633 | 3.44E-09 | down | feruloyl esterase A precursor |
| BBA_05394 | XM_008600491.1 | -2.628 | 4.27E-04 | down | hypothetical protein BBA_07770 |
| MSTRG.10861 | MSTRG.10861.2 | -2.622 | 7.29E-09 | down | ankyrin repeat domain-containing protein 52 |
| BBA_02379 | XM_008597476.1 | -2.605 | 1.13E-05 | down | C6 finger domain protein, putative |
| BBA_08131 | XM_008603228.1 | -2.593 | 2.53E-08 | down | Ankyrin repeat-containing protein |
| MSTRG.2509 | MSTRG.2509.2 | -2.591 | 2.83E-02 | down | -- |
| BBA_06600 | XM_008601697.1 | -2.588 | 1.44E-07 | down | acid phosphatase |
| BBA_03458 | XM_008598555.1 | -2.576 | 1.30E-08 | down | hypothetical protein BBA_08073 |
| BBA_10225 | XM_008605322.1 | -2.570 | 1.99E-06 | down | hypothetical protein BBA_08405 |
| MSTRG.4650 | MSTRG.4650.1 | -2.566 | 8.22E-07 | down | phosphoglycerate mutase family protein, putative |
| BBA_00687 | XM_008595784.1 | -2.554 | 1.67E-08 | down | hypothetical protein BBA_04636 |
| MSTRG.8173 | MSTRG.8173.1 | -2.552 | 3.52E-08 | down | Cytochrome P450 CYP5099A1 |
| BBA_09810 | XM_008604907.1 | -2.546 | 7.46E-07 | down | hypothetical protein BBA_09840 |
| BBA_10040 | XM_008605137.1 | -2.545 | 8.62E-08 | down | helix-loop-helix DNA-binding domain-containing protein |
| BBA_00428 | XM_008595525.1 | -2.542 | 1.22E-07 | down | fatty acid hydroxylase superfamily protein |
| BBA_10290 | XM_008605387.1 | -2.541 | 1.45E-03 | down | RTA1 domain-containing protein |
| BBA_07069 | XM_008602166.1 | -2.521 | 5.74E-08 | down | monooxygenase-like protein |
| BBA_00599 | XM_008595696.1 | -2.510 | 6.70E-08 | down | hypothetical protein BBA_10039 |
| MSTRG.12427 | MSTRG.12427.1 | -2.509 | 2.17E-08 | down | bacterial-type extracellular deoxyribonuclease |
| BBA_08073 | XM_008603170.1 | -2.495 | 2.54E-06 | down | phosphotransferase enzyme family protein |
| BBA_04717 | XM_008599814.1 | -2.481 | 1.93E-02 | down | -- |
| BBA_09806 | XM_008604903.1 | -2.468 | 3.88E-08 | down | hypothetical protein BBA_09033 |
| BBA_07503 | XM_008602600.1 | -2.465 | 7.53E-08 | down | Cytochrome P450 CYP539B1 |
| BBA_06599 | XM_008601696.1 | -2.462 | 2.79E-08 | down | adhesin protein Mad2 |
| BBA_02364 | XM_008597461.1 | -2.450 | 7.59E-08 | down | Alkaline phosphatase-like protein |
| BBA_00964 | XM_008596061.1 | -2.449 | 6.07E-03 | down | ferric reductase like transmembrane component |
| BBA_06744 | XM_008601841.1 | -2.448 | 2.57E-02 | down | -- |
| BBA_00968 | XM_008596065.1 | -2.445 | 5.74E-08 | down | phosphate transporter |
| BBA_02776 | XM_008597873.1 | -2.424 | 4.77E-04 | down | serine/threonine protein kinase |
| BBA_06750 | XM_008601847.1 | -2.411 | 1.13E-07 | down | adenylate-forming enzyme AfeA |
| BBA_09668 | XM_008604765.1 | -2.411 | 2.57E-02 | down | -- |
| MSTRG.10594 | MSTRG.10594.1 | -2.379 | 1.37E-07 | down | hemolysin-III family protein |
| BBA_04635 | XM_008599732.1 | -2.361 | 1.40E-07 | down | gp24-like protein |
| BBA_07118 | XM_008602215.1 | -2.356 | 9.06E-06 | down | flavin containing polyamine oxidase, putative |
| BBA_00037 | XM_008595134.1 | -2.346 | 2.71E-05 | down | sodium/hydrogen exchanger family protein |
| BBA_10347 | XM_008605444.1 | -2.345 | 2.39E-07 | down | Multicopper oxidase family protein |
| BBA_10011 | XM_008605108.1 | -2.342 | 1.79E-02 | down | hypothetical protein BBA_03917 |
| BBA_02033 | XM_008597130.1 | -2.336 | 1.43E-06 | down | phosphatidylinositol-specific phospholipase C |
| BBA_00850 | XM_008595947.1 | -2.305 | 1.24E-04 | down | high-affinity methionine permease |
| BBA_01015 | XM_008596112.1 | -2.305 | 4.10E-07 | down | methyltransferase-like protein |
| BBA_02286 | XM_008597383.1 | -2.279 | 9.32E-05 | down | -- |
| BBA_04975 | XM_008600072.1 | -2.259 | 9.51E-03 | down | transposase-like protein |
| MSTRG.8214 | MSTRG.8214.1 | -2.256 | 2.07E-05 | down | Chitinase II |
| BBA_09427 | XM_008604524.1 | -2.252 | 8.31E-07 | down | TfdA family Taurine catabolism dioxygenase TauD |
| BBA_07770 | XM_008602867.1 | -2.248 | 1.43E-06 | down | hypothetical protein BBA_06001 |
| BBA_07369 | XM_008602466.1 | -2.247 | 1.35E-06 | down | protein tyrosine phosphatase (Pyp1), putative |
| MSTRG.4513 | MSTRG.4513.1 | -2.242 | 2.51E-06 | down | hypothetical protein BBA_07700 |
| BBA_07652 | XM_008602749.1 | -2.224 | 2.26E-05 | down | OPT oligopeptide transporter |
| BBA_05273 | XM_008600370.1 | -2.199 | 9.36E-07 | down | acid phosphatase |
| MSTRG.7896 | MSTRG.7896.2 | -2.197 | 2.89E-03 | down | -- |
| BBA_05767 | XM_008600864.1 | -2.193 | 2.37E-03 | down | hypothetical protein BBA_00259 |
| MSTRG.10657 | MSTRG.10657.2 | -2.192 | 8.20E-05 | down | 4-hydroxyacetophenone monooxygenase |
| MSTRG.896 | MSTRG.896.1 | -2.192 | 9.27E-07 | down | hypothetical protein BBA_09033 |
| BBA_02327 | XM_008597424.1 | -2.182 | 9.12E-06 | down | Ribonuclease/ribotoxin |
| BBA_09839 | XM_008604936.1 | -2.180 | 2.20E-06 | down | putative SERINE-TYPE CARBOXYPEPTIDASE F PRECURSOR |
| BBA_02800 | XM_008597897.1 | -2.177 | 2.92E-06 | down | filamentous hemagglutinin / adhesin |
| BBA_00259 | XM_008595356.1 | -2.169 | 1.17E-04 | down | LysR family regulatory protein |
| BBA_05340 | XM_008600437.1 | -2.166 | 1.44E-06 | down | hypothetical protein BBA_10004 |
| BBA_08666 | XM_008603763.1 | -2.166 | 3.96E-02 | down | ATP synthase F1 |
| BBA_07234 | XM_008602331.1 | -2.156 | 7.66E-06 | down | hypothetical protein BBA_08793 |
| MSTRG.4116 | MSTRG.4116.1 | -2.151 | 2.39E-06 | down | Cytochrome P450 CYP548A5 |
| BBA_02099 | XM_008597196.1 | -2.138 | 5.11E-06 | down | metallo-beta-lactamase superfamily protein |
| BBA_06472 | XM_008601569.1 | -2.133 | 4.01E-06 | down | histidine acid phosphatase |
| BBA_04636 | XM_008599733.1 | -2.132 | 2.98E-06 | down | eukaryotic aspartyl protease |
| BBA_00690 | XM_008595787.1 | -2.126 | 3.41E-04 | down | carbon-nitrogen hydrolase |
| BBA_01342 | XM_008596439.1 | -2.122 | 4.09E-03 | down | -- |
| BBA_08997 | XM_008604094.1 | -2.122 | 4.97E-05 | down | hypothetical protein BBA_06679 |
| BBA_09669 | XM_008604766.1 | -2.118 | 2.13E-04 | down | hypothetical protein BBA_00769 |
| BBA_10041 | XM_008605138.1 | -2.118 | 2.25E-05 | down | formyl transferase domain-containing protein |
| BBA_09890 | XM_008604987.1 | -2.101 | 3.10E-05 | down | -- |
| BBA_08793 | XM_008603890.1 | -2.100 | 1.56E-04 | down | K-family cellulase |
| BBA_06151 | XM_008601248.1 | -2.096 | 1.39E-05 | down | pheromone receptor |
| BBA_07958 | XM_008603055.1 | -2.093 | 3.34E-04 | down | hypothetical protein BBA_04251 |
| BBA_09840 | XM_008604937.1 | -2.081 | 4.91E-06 | down | S1/P1 nuclease |
| BBA_08393 | XM_008603490.1 | -2.078 | 1.28E-02 | down | hypothetical protein BBA_07521 |
| BBA_06999 | XM_008602096.1 | -2.074 | 2.25E-05 | down | hypothetical protein BBA_08127 |
| BBA_02653 | XM_008597750.1 | -2.070 | 1.78E-04 | down | SWIM zinc finger protein |
| BBA_10039 | XM_008605136.1 | -2.069 | 8.77E-06 | down | Cytochrome P450 CYP620D1 |
| BBA_01677 | XM_008596774.1 | -2.064 | 1.02E-02 | down | -- |
| BBA_05071 | XM_008600168.1 | -2.057 | 1.06E-05 | down | hypothetical protein BBA_00690 |
| BBA_03704 | XM_008598801.1 | -2.053 | 2.16E-02 | down | feebly protein |
| BBA_09773 | XM_008604870.1 | -2.036 | 3.34E-04 | down | NmrA-like family protein |
| BBA_03308 | XM_008598405.1 | -2.034 | 6.33E-06 | down | hypothetical protein BBA_06418 |
| BBA_06475 | XM_008601572.1 | -2.032 | 1.77E-05 | down | C4-dicarboxylate transporter/malic acid transporter |
| BBA_02952 | XM_008598049.1 | -2.032 | 6.46E-06 | down | phosphatidylethanolamine N-methyltransferase |
| BBA_06864 | XM_008601961.1 | -2.028 | 1.69E-04 | down | hypothetical protein BBA_05790 |
| BBA_08789 | XM_008603886.1 | -2.017 | 5.29E-04 | down | choline transport protein |
| BBA_03222 | XM_008598319.1 | -2.003 | 1.68E-05 | down | MedA-like protein |
| BBA_09498 | XM_008604595.1 | -1.997 | 1.05E-05 | down | lipase/thioesterase family protein |
| MSTRG.3516 | MSTRG.3516.1 | -1.996 | 1.65E-05 | down | ferric reductase like transmembrane component |
| BBA_10179 | XM_008605276.1 | -1.995 | 1.36E-05 | down | ankyrin repeat protein |
| BBA_05439 | XM_008600536.1 | -1.995 | 1.09E-05 | down | phosphorylcholine phosphatase |
| BBA_01240 | XM_008596337.1 | -1.991 | 1.28E-04 | down | hypothetical protein BBA_07717 |
| BBA_09459 | XM_008604556.1 | -1.990 | 1.44E-02 | down | major facilitator superfamily transporter |
| MSTRG.9538 | MSTRG.9538.1 | -1.989 | 1.08E-05 | down | taurine catabolism dioxygenase TauD |
| BBA_09033 | XM_008604130.1 | -1.987 | 1.09E-05 | down | dethiobiotin synthetase |
| BBA_10004 | XM_008605101.1 | -1.984 | 1.30E-04 | down | inorganic pyrophosphatase |
| BBA_05605 | XM_008600702.1 | -1.980 | 2.25E-04 | down | cyclopropane-fatty-acyl-phospholipid synthase |
| BBA_10063 | XM_008605160.1 | -1.966 | 3.06E-05 | down | palmitoyl protein thioesterase |
| BBA_00305 | XM_008595402.1 | -1.965 | 4.79E-02 | down | Pfs, NACHT, and Ankyrin domain protein |
| BBA_08072 | XM_008603169.1 | -1.964 | 1.57E-05 | down | hypothetical protein BBA_08623 |
| MSTRG.12604 | MSTRG.12604.1 | -1.961 | 1.57E-05 | down | -- |
| MSTRG.8081 | MSTRG.8081.1 | -1.954 | 7.47E-03 | down | transcriptional regulator, putative |
| BBA_04825 | XM_008599922.1 | -1.953 | 1.02E-02 | down | polyketide synthase |
| BBA_04320 | XM_008599417.1 | -1.952 | 1.06E-03 | down | hypothetical protein BBA_04798 |
| BBA_10303 | XM_008605400.1 | -1.944 | 6.71E-05 | down | carbonic anhydrase |
| BBA_08973 | XM_008604070.1 | -1.932 | 4.79E-02 | down | penicillin-binding protein |
| BBA_06679 | XM_008601776.1 | -1.924 | 1.89E-04 | down | hypothetical protein BBA_08839 |
| BBA_03283 | XM_008598380.1 | -1.923 | 5.38E-04 | down | arylsulfatase, putative |
| BBA_05741 | XM_008600838.1 | -1.916 | 1.18E-04 | down | vacuolar iron transporter Ccc1 |
| BBA_07285 | XM_008602382.1 | -1.915 | 3.73E-05 | down | canalicular multispecific organic anion transporter 1 |
| MSTRG.6513 | MSTRG.6513.1 | -1.914 | 2.62E-03 | down | -- |
| MSTRG.3760 | MSTRG.3760.1 | -1.913 | 8.73E-04 | down | OPT oligopeptide transporter |
| BBA_03206 | XM_008598303.1 | -1.906 | 1.11E-04 | down | apurinic endonuclease |
| BBA_07854 | XM_008602951.1 | -1.897 | 6.38E-04 | down | sodium symporter family protein |
| BBA_02152 | XM_008597249.1 | -1.887 | 4.27E-05 | down | metabolite transport protein GIT1 |
| MSTRG.4742 | MSTRG.4742.1 | -1.880 | 6.11E-05 | down | zinc-binding dehydrogenase |
| BBA_05154 | XM_008600251.1 | -1.880 | 4.43E-04 | down | hypothetical protein BBA_04320 |
| BBA_08839 | XM_008603936.1 | -1.875 | 7.48E-04 | down | hypothetical protein BBA_09467 |
| MSTRG.5855 | MSTRG.5855.1 | -1.873 | 1.15E-03 | down | UV-endonuclease UvdE |
| BBA_00769 | XM_008595866.1 | -1.867 | 2.01E-04 | down | hypothetical protein BBA_08502 |
| BBA_04631 | XM_008599728.1 | -1.851 | 6.22E-03 | down | hypothetical protein BBA_01595 |
| BBA_07700 | XM_008602797.1 | -1.851 | 9.54E-05 | down | lipase class 2 |
| BBA_08715 | XM_008603812.1 | -1.845 | 1.67E-04 | down | polyketide synthase |
| MSTRG.12616 | MSTRG.12616.1 | -1.839 | 6.76E-05 | down | phosphate:H+ symporter |
| BBA_07521 | XM_008602618.1 | -1.838 | 3.22E-04 | down | dopa 4,5-dioxygenase |
| BBA_08916 | XM_008604013.1 | -1.837 | 7.15E-04 | down | major facilitator superfamily transporter |
| BBA_06751 | XM_008601848.1 | -1.833 | 8.72E-04 | down | ABC transporter with duplicated ATPase domains |
| BBA_06273 | XM_008601370.1 | -1.832 | 9.54E-05 | down | Cytochrome P450 CYP684A2 |
| BBA_04389 | XM_008599486.1 | -1.830 | 1.43E-04 | down | hypothetical protein BBA_08764 |
| BBA_10154 | XM_008605251.1 | -1.830 | 6.67E-05 | down | Cytochrome P450 CYP542B3 |
| MSTRG.12594 | MSTRG.12594.2 | -1.827 | 1.20E-04 | down | C6 finger domain protein |
| BBA_04401 | XM_008599498.1 | -1.826 | 8.50E-05 | down | sporulation-specific protein |
| BBA_04843 | XM_008599940.1 | -1.820 | 5.22E-04 | down | calcineurin-like phosphoesterase |
| MSTRG.1750 | MSTRG.1750.1 | -1.814 | 2.49E-04 | down | peptide synthetase |
| BBA_06341 | XM_008601438.1 | -1.807 | 5.70E-03 | down | tetracycline efflux protein (otrb) |
| MSTRG.3515 | MSTRG.3515.1 | -1.805 | 1.16E-04 | down | cucumopine synthase |
| BBA_01016 | XM_008596113.1 | -1.805 | 1.23E-04 | down | protease S8 tripeptidyl peptidase I (cln2) |
| BBA_04271 | XM_008599368.1 | -1.803 | 2.86E-04 | down | hypothetical protein BBA_02951 |
| BBA_02312 | XM_008597409.1 | -1.803 | 1.73E-04 | down | enoyl-CoA hydratase/isomerase family protein |
| MSTRG.10615 | MSTRG.10615.1 | -1.793 | 1.85E-02 | down | hypothetical protein VHEMI08667 |
| BBA_08127 | XM_008603224.1 | -1.788 | 3.23E-04 | down | -- |
| MSTRG.11965 | MSTRG.11965.1 | -1.785 | 3.08E-04 | down | nitrate assimilation regulatory protein nirA |
| MSTRG.10692 | MSTRG.10692.1 | -1.782 | 6.60E-04 | down | histidine acid phosphatase |
| BBA_07463 | XM_008602560.1 | -1.778 | 1.57E-02 | down | putative SAM-dependent methyltransferase |
| BBA_03909 | XM_008599006.1 | -1.773 | 1.24E-04 | down | trascription factor |
| BBA_09514 | XM_008604611.1 | -1.773 | 1.00E-02 | down | hypothetical protein BBA_08813 |
| BBA_08085 | XM_008603182.1 | -1.769 | 1.74E-04 | down | dibenzothiophene desulfurization enzyme A |
| BBA_05559 | XM_008600656.1 | -1.769 | 6.70E-04 | down | Acyl-CoA N-acyltransferase |
| BBA_08623 | XM_008603720.1 | -1.764 | 6.42E-04 | down | putative aspartic protease |
| BBA_04178 | XM_008599275.1 | -1.760 | 5.68E-04 | down | aspartic proteinase |
| MSTRG.5889 | MSTRG.5889.1 | -1.760 | 1.78E-04 | down | homogentisate 1,2-dioxygenase |
| BBA_01515 | XM_008596612.1 | -1.743 | 1.65E-04 | down | aminotransferase class I and II |
| BBA_07494 | XM_008602591.1 | -1.710 | 6.97E-03 | down | hypothetical protein LLEC1_02252 |
| BBA_10242 | XM_008605339.1 | -1.707 | 9.57E-04 | down | hypothetical protein BBA_04720 |
| BBA_04908 | XM_008600005.1 | -1.705 | 2.42E-04 | down | Peptidase cysteine/serine, trypsin |
| BBA_08030 | XM_008603127.1 | -1.703 | 2.55E-02 | down | hypothetical protein BBA_06796 |
| BBA_06319 | XM_008601416.1 | -1.700 | 1.07E-02 | down | Pre-mRNA-splicing factor srp2 |
| BBA_01665 | XM_008596762.1 | -1.695 | 3.24E-03 | down | C6 finger domain protein |
| MSTRG.7321 | MSTRG.7321.1 | -1.694 | 1.91E-02 | down | thiol-specific monooxygenase |
| BBA_05790 | XM_008600887.1 | -1.693 | 4.60E-04 | down | sterigmatocystin 8-O-methyltransferase precursor, putative |
| BBA_04251 | XM_008599348.1 | -1.688 | 2.90E-04 | down | Peptidase cysteine/serine, trypsin |
| BBA_09418 | XM_008604515.1 | -1.684 | 4.88E-02 | down | MFS quinate transporter QutD |
| BBA_07117 | XM_008602214.1 | -1.681 | 6.47E-03 | down | dipeptidyl peptidase III |
| BBA_04181 | XM_008599278.1 | -1.680 | 3.21E-04 | down | fatty acid hydroxylase superfamily protein |
| MSTRG.9531 | MSTRG.9531.1 | -1.676 | 3.38E-04 | down | hypothetical protein BBA_00688 |
| BBA_06710 | XM_008601807.1 | -1.671 | 1.20E-03 | down | Cytochrome P450 CYP542B2 |
| MSTRG.3498 | MSTRG.3498.1 | -1.668 | 7.16E-04 | down | aspartate-tRNA ligase |
| BBA_07771 | XM_008602868.1 | -1.667 | 4.13E-04 | down | zinc finger protein |
| BBA_10240 | XM_008605337.1 | -1.667 | 2.94E-03 | down | hypothetical protein BBA_01424 |
| BBA_09142 | XM_008604239.1 | -1.666 | 5.09E-04 | down | choline-sulfatase-like protein |
| BBA_10302 | XM_008605399.1 | -1.660 | 1.37E-03 | down | subtilase-like protein |
| BBA_06473 | XM_008601570.1 | -1.656 | 1.56E-03 | down | Acyl-CoA N-acyltransferase |
| BBA_04123 | XM_008599220.1 | -1.656 | 4.13E-04 | down | short chain dehydrogenase |
| BBA_02920 | XM_008598017.1 | -1.653 | 5.10E-04 | down | indoleamine 2,3-dioxygenase family protein |
| BBA_08434 | XM_008603531.1 | -1.651 | 1.34E-03 | down | hypothetical protein BBA_09447 |
| BBA_09473 | XM_008604570.1 | -1.651 | 5.38E-04 | down | HMG box protein |
| MSTRG.13164 | MSTRG.13164.1 | -1.649 | 3.67E-03 | down | hypothetical protein BBA_07671 |
| BBA_05740 | XM_008600837.1 | -1.647 | 5.34E-04 | down | indoleamine 2,3-dioxygenase |
| BBA_09373 | XM_008604470.1 | -1.645 | 5.62E-04 | down | CFEM domain-containing protein |
| BBA_05155 | XM_008600252.1 | -1.645 | 1.01E-02 | down | allantoate permease |
| BBA_10091 | XM_008605188.1 | -1.633 | 7.86E-04 | down | phosphoesterase-like protein |
| BBA_04959 | XM_008600056.1 | -1.632 | 4.93E-04 | down | glutamate/Leucine/Phenylalanine/Valine dehydrogenase |
| MSTRG.10727 | MSTRG.10727.1 | -1.631 | 5.68E-04 | down | Cytochrome P450 CYP6004A2 |
| MSTRG.10374 | MSTRG.10374.1 | -1.630 | 5.72E-04 | down | putative hexosaminidase |
| MSTRG.2847 | MSTRG.2847.1 | -1.627 | 4.29E-03 | down | hypothetical protein BBA_09281 |
| BBA_09467 | XM_008604564.1 | -1.623 | 1.18E-03 | down | hypothetical protein BBA_00913 |
| BBA_05122 | XM_008600219.1 | -1.622 | 1.88E-02 | down | hypothetical protein BBA_00666 |
| BBA_07617 | XM_008602714.1 | -1.621 | 5.76E-04 | down | SPX domain-containing protein |
| MSTRG.8502 | MSTRG.8502.1 | -1.614 | 2.59E-03 | down | aminotransferase class-III |
| BBA_00212 | XM_008595309.1 | -1.608 | 1.48E-02 | down | major facilitator superfamily transporter |
| BBA_00200 | XM_008595297.1 | -1.607 | 2.55E-03 | down | glycoside hydrolase family 55 |
| BBA_09825 | XM_008604922.1 | -1.604 | 6.70E-04 | down | cutinase-like protein |
| BBA_03547 | XM_008598644.1 | -1.596 | 1.82E-02 | down | -- |
| BBA_06350 | XM_008601447.1 | -1.596 | 3.67E-02 | down | tat pathway signal sequence |
| BBA_04798 | XM_008599895.1 | -1.595 | 7.05E-04 | down | methyltransferase domain-containing protein |
| BBA_02182 | XM_008597279.1 | -1.595 | 3.06E-02 | down | hypothetical protein BBA_10341 |
| BBA_10300 | XM_008605397.1 | -1.595 | 7.16E-04 | down | Cytochrome P450 CYP625A1 |
| MSTRG.51 | MSTRG.51.1 | -1.592 | 4.23E-02 | down | proclavaminate amidinohydrolase |
| BBA_10107 | XM_008605204.1 | -1.589 | 1.42E-03 | down | phospholipase D2 |
| BBA_04242 | XM_008599339.1 | -1.588 | 8.35E-04 | down | transaldolase-like protein |
| BBA_04824 | XM_008599921.1 | -1.581 | 1.48E-03 | down | hypothetical protein BBA_10342 |
| BBA_01623 | XM_008596720.1 | -1.577 | 1.01E-03 | down | AMP-binding enzyme |
| BBA_06552 | XM_008601649.1 | -1.576 | 1.67E-03 | down | hypothetical protein BBA_09776 |
| BBA_03110 | XM_008598207.1 | -1.573 | 9.02E-04 | down | multidrug resistance protein 1 |
| BBA_06474 | XM_008601571.1 | -1.569 | 2.54E-03 | down | hypothetical protein BBA_09444 |
| BBA_05449 | XM_008600546.1 | -1.565 | 1.01E-03 | down | xanthine dehydrogenase |
| BBA_04827 | XM_008599924.1 | -1.558 | 2.22E-03 | down | extracellular aldonolactonase |
| BBA_04720 | XM_008599817.1 | -1.558 | 3.70E-03 | down | D-isomer specific 2-hydroxyacid dehydrogenase |
| MSTRG.12444 | MSTRG.12444.1 | -1.555 | 4.04E-03 | down | oxidoreductase, 2OG-Fe(II) oxygenase family |
| BBA_02281 | XM_008597378.1 | -1.554 | 1.29E-02 | down | -- |
| BBA_02326 | XM_008597423.1 | -1.552 | 9.74E-04 | down | mitochondrial peroxiredoxin PRX1 |
| BBA_05874 | XM_008600971.1 | -1.548 | 4.87E-03 | down | MFS drug efflux transporter, putative |
| BBA_04727 | XM_008599824.1 | -1.544 | 1.29E-03 | down | -- |
| BBA_09472 | XM_008604569.1 | -1.543 | 3.52E-03 | down | aromatic amino acid aminotransferase |
| BBA_00051 | XM_008595148.1 | -1.543 | 1.23E-02 | down | endonuclease/exonuclease/phosphatase family |
| BBA_07525 | XM_008602622.1 | -1.541 | 3.39E-02 | down | calcium-transporting ATPase |
| BBA_01595 | XM_008596692.1 | -1.541 | 1.32E-03 | down | hypothetical protein BBA_06708 |
| BBA_01149 | XM_008596246.1 | -1.535 | 4.03E-02 | down | hypothetical protein BBA_00051 |
| MSTRG.9807 | MSTRG.9807.1 | -1.534 | 1.64E-03 | down | hypothetical protein BBA_06621 |
| BBA_08502 | XM_008603599.1 | -1.527 | 1.24E-03 | down | Cytochrome P450 CYP542B3 |
| BBA_02951 | XM_008598048.1 | -1.521 | 2.57E-03 | down | FAD binding domain-containing protein |
| BBA_01283 | XM_008596380.1 | -1.520 | 1.46E-03 | down | Zn-dependent alcohol dehydrogenases (ISS) |
| BBA_04063 | XM_008599160.1 | -1.512 | 3.47E-03 | down | multicopper oxidase |
| BBA_08764 | XM_008603861.1 | -1.512 | 1.57E-03 | down | MFS transporter |
| BBA_04401 | XM_008599498.1 | -1.499 | 1.83E-03 | down | perilipin MPL1-like protein |
| BBA_01992 | XM_008597089.1 | -1.499 | 1.86E-03 | down | beta-galactosidase |
| BBA_09225 | XM_008604322.1 | -1.497 | 5.11E-03 | down | hypothetical protein BBA_09459 |
| MSTRG.14394 | MSTRG.14394.1 | -1.490 | 9.41E-03 | down | proline dehydrogenase |
| BBA_10055 | XM_008605152.1 | -1.484 | 6.27E-03 | down | hypothetical protein BBA_08529 |
| BBA_05008 | XM_008600105.1 | -1.480 | 2.41E-02 | down | nicotinate-nucleotide diphosphorylase |
| BBA_04976 | XM_008600073.1 | -1.477 | 2.58E-03 | down | -- |
| BBA_09447 | XM_008604544.1 | -1.475 | 5.18E-03 | down | ABC transporter |
| BBA_09318 | XM_008604415.1 | -1.474 | 2.11E-03 | down | cholinephosphotransferase-like protein |
| BBA_03956 | XM_008599053.1 | -1.472 | 4.03E-03 | down | DUF323 domain-containing protein |
| BBA_00754 | XM_008595851.1 | -1.471 | 4.01E-02 | down | hypothetical protein CTHT_0044350 |
| BBA_00196 | XM_008595293.1 | -1.467 | 7.91E-03 | down | fatty acid hydroxylase superfamily protein |
| BBA_05412 | XM_008600509.1 | -1.465 | 7.35E-03 | down | hypothetical protein BBA_03547 |
| BBA_04936 | XM_008600033.1 | -1.459 | 9.82E-03 | down | allantoate permease |
| BBA_08898 | XM_008603995.1 | -1.451 | 3.71E-03 | down | Malate synthase, glyoxysomal |
| BBA_02722 | XM_008597819.1 | -1.447 | 2.80E-03 | down | hypothetical protein BBA_05122 |
| BBA_01214 | XM_008596311.1 | -1.445 | 5.29E-03 | down | hypothetical protein BBA_07014 |
| BBA_00438 | XM_008595535.1 | -1.444 | 2.66E-03 | down | GTP-binding protein EsdC |
| BBA_09661 | XM_008604758.1 | -1.440 | 3.49E-03 | down | hypothetical protein BBA_09470 |
| MSTRG.13149 | MSTRG.13149.1 | -1.435 | 1.08E-02 | down | Dephospho-CoA kinase |
| BBA_08813 | XM_008603910.1 | -1.435 | 3.06E-03 | down | guanine nucleotide exchange factor synembryn |
| BBA_08501 | XM_008603598.1 | -1.432 | 3.01E-03 | down | hypothetical protein BBA_09967 |
| BBA_09412 | XM_008604509.1 | -1.432 | 3.03E-03 | down | hypothetical protein BBAD15_g4409 |
| BBA_09660 | XM_008604757.1 | -1.431 | 1.22E-02 | down | -- |
| BBA_05739 | XM_008600836.1 | -1.430 | 3.12E-03 | down | taurine catabolism dioxygenase TauD |
| BBA_01701 | XM_008596798.1 | -1.426 | 3.98E-03 | down | acetyltransferase, GNAT family protein |
| MSTRG.10701 | MSTRG.10701.1 | -1.421 | 5.47E-03 | down | -- |
| MSTRG.4787 | MSTRG.4787.1 | -1.420 | 3.89E-03 | down | choline transporter |
| BBA_09470 | XM_008604567.1 | -1.420 | 1.98E-02 | down | MFS multidrug transporter, putative |
| BBA_04084 | XM_008599181.1 | -1.419 | 3.40E-03 | down | cytosol Mn-superoxide dismutase |
| BBA_01557 | XM_008596654.1 | -1.418 | 4.70E-03 | down | hypothetical protein BBA_02534 |
| BBA_05291 | XM_008600388.1 | -1.412 | 4.02E-03 | down | hypothetical protein BBA_09540 |
| BBA_04240 | XM_008599337.1 | -1.409 | 1.35E-02 | down | phosphoglycerate mutase |
| BBA_04371 | XM_008599468.1 | -1.409 | 2.23E-02 | down | heterokaryon incompatibility protein |
| BBA_09119 | XM_008604216.1 | -1.408 | 6.01E-03 | down | short chain dehydrogenase |
| BBA_04826 | XM_008599923.1 | -1.408 | 4.62E-03 | down | C2H2 type zinc finger domain-containing protein |
| MSTRG.9223 | MSTRG.9223.1 | -1.405 | 2.18E-02 | down | hypothetical protein BBA_06002 |
| BBA_08759 | XM_008603856.1 | -1.405 | 1.42E-02 | down | hypothetical protein BBA_05008 |
| BBA_05303 | XM_008600400.1 | -1.402 | 4.73E-03 | down | Amine oxidase |
| BBA_07918 | XM_008603015.1 | -1.401 | 3.15E-02 | down | hypothetical protein BBA_08971 |
| MSTRG.12085 | MSTRG.12085.2 | -1.400 | 7.86E-03 | down | hypothetical protein BBA_08030 |
| BBA_09396 | XM_008604493.1 | -1.397 | 1.41E-02 | down | hypothetical protein BBA_02789 |
| BBA_07554 | XM_008602651.1 | -1.394 | 4.14E-03 | down | Cytochrome P450 CYP504B10 |
| BBA_10346 | XM_008605443.1 | -1.393 | 5.59E-03 | down | hypothetical protein BBA_06744 |
| BBA_09769 | XM_008604866.1 | -1.393 | 4.32E-03 | down | hypothetical protein BBA_09668 |
| BBA_02876 | XM_008597973.1 | -1.392 | 6.76E-03 | down | vivid PAS protein VVD |
| BBA_00246 | XM_008595343.1 | -1.392 | 7.83E-03 | down | -- |
| BBA_10010 | XM_008605107.1 | -1.391 | 4.54E-03 | down | MFS multidrug transporter |
| MSTRG.4298 | MSTRG.4298.1 | -1.384 | 7.17E-03 | down | hypothetical protein BBA_05675 |
| BBA_00528 | XM_008595625.1 | -1.383 | 4.96E-03 | down | lovastatin nonaketide synthase |
| MSTRG.9885 | MSTRG.9885.1 | -1.383 | 1.57E-02 | down | reverse transcriptase, RNaseH |
| BBA_06056 | XM_008601153.1 | -1.382 | 5.70E-03 | down | -- |
| BBA_03179 | XM_008598276.1 | -1.381 | 1.15E-02 | down | major facilitator superfamily transporter |
| MSTRG.10409 | MSTRG.10409.1 | -1.381 | 4.67E-03 | down | hypothetical protein BBA_05637 |
| MSTRG.9130 | MSTRG.9130.1 | -1.371 | 6.77E-03 | down | hypothetical protein BBAD15_g9227 |
| BBA_04896 | XM_008599993.1 | -1.371 | 2.80E-02 | down | cholinesterase-like protein |
| BBA_09446 | XM_008604543.1 | -1.370 | 7.07E-03 | down | Pfs domain protein |
| BBA_08371 | XM_008603468.1 | -1.366 | 5.07E-03 | down | UTP-glucose-1-phosphate uridylyltransferase |
| BBA_04743 | XM_008599840.1 | -1.361 | 3.49E-02 | down | nitrite transporter |
| BBA_03639 | XM_008598736.1 | -1.360 | 8.49E-03 | down | FAD binding domain-containing protein |
| BBA_00666 | XM_008595763.1 | -1.358 | 6.81E-03 | down | Bicarbonate transporter |
| MSTRG.542 | MSTRG.542.1 | -1.354 | 4.57E-02 | down | BTB/POZ domain protein |
| BBA_09774 | XM_008604871.1 | -1.353 | 3.47E-02 | down | glucose/galactose transporter |
| BBA_07970 | XM_008603067.1 | -1.350 | 9.23E-03 | down | MFS transporter |
| BBA_01923 | XM_008597020.1 | -1.348 | 2.01E-02 | down | monooxygenase-like protein |
| BBA_01598 | XM_008596695.1 | -1.348 | 8.80E-03 | down | hypothetical protein BBAD15_g11927 |
| BBA_02516 | XM_008597613.1 | -1.348 | 1.87E-02 | down | protein kinase domain-containing protein |
| BBA_04761 | XM_008599858.1 | -1.348 | 1.37E-02 | down | fusicoccadiene synthase |
| BBA_04013 | XM_008599110.1 | -1.342 | 7.82E-03 | down | Cytochrome P450 CYP5099A1 |
| BBA_07120 | XM_008602217.1 | -1.340 | 8.18E-03 | down | methionine transporter, putative |
| BBA_10221 | XM_008605318.1 | -1.338 | 3.89E-02 | down | FAD dependent oxidoreductase |
| BBA_04065 | XM_008599162.1 | -1.337 | 3.39E-02 | down | C6 transcription factor |
| BBA_09281 | XM_008604378.1 | -1.336 | 6.63E-03 | down | OPT peptide transporter Mtd1 |
| MSTRG.12133 | MSTRG.12133.1 | -1.333 | 1.58E-02 | down | hexose transporter |
| BBA_06621 | XM_008601718.1 | -1.331 | 1.23E-02 | down | PRO41 protein |
| BBA_10341 | XM_008605438.1 | -1.330 | 8.40E-03 | down | DnaJ domain-containing protein |
| BBA_01664 | XM_008596761.1 | -1.327 | 4.74E-02 | down | hypothetical protein BBA_00654 |
| BBA_00426 | XM_008595523.1 | -1.327 | 9.19E-03 | down | L-serine dehydratase |
| BBA_09449 | XM_008604546.1 | -1.321 | 4.35E-02 | down | MFS transporter |
| BBA_02077 | XM_008597174.1 | -1.315 | 8.12E-03 | down | Catalase-like domain, heme-dependent |
| MSTRG.972 | MSTRG.972.1 | -1.315 | 1.47E-02 | down | serine/threonine kinase |
| BBA_05618 | XM_008600715.1 | -1.315 | 9.22E-03 | down | D-galacturonic acid reductase |
| BBA_00944 | XM_008596041.1 | -1.311 | 3.15E-02 | down | alpha-1,3-mannosyltransferase CMT1 |
| BBA_04779 | XM_008599876.1 | -1.306 | 1.35E-02 | down | hypothetical protein BBA_06000 |
| BBA_07683 | XM_008602780.1 | -1.295 | 4.74E-02 | down | zeta toxin |
| BBA_09776 | XM_008604873.1 | -1.292 | 9.61E-03 | down | IDC1 protein |
| BBA_08947 | XM_008604044.1 | -1.289 | 3.30E-02 | down | potassium/sodium efflux P-type ATPase |
| MSTRG.8406 | MSTRG.8406.1 | -1.289 | 3.00E-02 | down | integral membrane protein |
| MSTRG.4104 | MSTRG.4104.1 | -1.286 | 4.47E-02 | down | pantothenate transporter |
| BBA_09444 | XM_008604541.1 | -1.286 | 9.98E-03 | down | glutathione S-transferase II |
| MSTRG.8451 | MSTRG.8451.1 | -1.285 | 2.09E-02 | down | potassium/sodium efflux P-type ATPase |
| BBA_05282 | XM_008600379.1 | -1.283 | 1.75E-02 | down | Histone H5 |
| BBA_07590 | XM_008602687.1 | -1.277 | 1.10E-02 | down | guanine nucleotide exchange factor synembryn |
| MSTRG.827 | MSTRG.827.1 | -1.274 | 1.08E-02 | down | tripeptidyl peptidase A |
| BBA_06708 | XM_008601805.1 | -1.273 | 1.19E-02 | down | hypothetical protein BBA_01162 |
| BBA_07082 | XM_008602179.1 | -1.273 | 4.11E-02 | down | hypothetical protein BBA_05507 |
| BBA_03638 | XM_008598735.1 | -1.269 | 1.35E-02 | down | DNA photolyase |
| BBA_08387 | XM_008603484.1 | -1.268 | 1.15E-02 | down | biotin synthase |
| MSTRG.10689 | MSTRG.10689.1 | -1.268 | 4.49E-02 | down | C6 zinc finger domain-containing protein |
| BBA_08911 | XM_008604008.1 | -1.267 | 2.38E-02 | down | zeta toxin |
| BBA_07024 | XM_008602121.1 | -1.267 | 2.38E-02 | down |  |
| BBA_10310 | XM_008605407.1 | -1.267 | 2.03E-02 | down | Cytochrome P450 CYP628A2 |
| BBA_04686 | XM_008599783.1 | -1.266 | 1.49E-02 | down | hypothetical protein BBA_08973 |
| MSTRG.8551 | MSTRG.8551.1 | -1.264 | 1.14E-02 | down | hypothetical protein BBA_04334 |
| BBA_08498 | XM_008603595.1 | -1.263 | 1.15E-02 | down | guanine nucleotide exchange factor synembryn |
| BBA_06002 | XM_008601099.1 | -1.262 | 2.40E-02 | down | DNA repair protein (Rad57), putative |
| BBA_08529 | XM_008603626.1 | -1.259 | 1.49E-02 | down | pheromone-regulated membrane protein |
| MSTRG.13910 | MSTRG.13910.1 | -1.257 | 1.80E-02 | down | BTB/POZ domain containing protein |
| BBA_05603 | XM_008600700.1 | -1.254 | 3.96E-02 | down | isoprenylcysteine carboxyl methyltransferase |
| BBA_04007 | XM_008599104.1 | -1.254 | 1.25E-02 | down | hypothetical protein BBA_05637 |
| BBA_06411 | XM_008601508.1 | -1.254 | 3.59E-02 | down | -- |
| BBA_09573 | XM_008604670.1 | -1.253 | 1.26E-02 | down | beta-glucosidase |
| BBA_02789 | XM_008597886.1 | -1.252 | 2.57E-02 | down | cercosporin toxin biosynthesis protein |
| MSTRG.14397 | MSTRG.14397.1 | -1.252 | 1.26E-02 | down | ABC transporter |
| BBA_08447 | XM_008603544.1 | -1.250 | 1.51E-02 | down | Major Facilitator Superfamily protein |
| MSTRG.13736 | MSTRG.13736.1 | -1.249 | 1.53E-02 | down | hypothetical protein BBA_07859 |
| BBA_04180 | XM_008599277.1 | -1.242 | 3.36E-02 | down | K-3-type glutaminase |
| BBA_05554 | XM_008600651.1 | -1.240 | 1.55E-02 | down | finger protein AZF1 |
| BBA_06425 | XM_008601522.1 | -1.239 | 1.43E-02 | down | PH domain-containing protein |
| BBA_07744 | XM_008602841.1 | -1.231 | 3.63E-02 | down | hypothetical protein BBA_07936 |
| BBA_06921 | XM_008602018.1 | -1.228 | 4.60E-02 | down | hypothetical protein BBA_08825 |
| BBA_07014 | XM_008602111.1 | -1.225 | 1.89E-02 | down | cupin 2 |
| MSTRG.10291 | MSTRG.10291.1 | -1.225 | 1.97E-02 | down | vacuolar calcium ion transporter |
| BBA_09280 | XM_008604377.1 | -1.221 | 1.59E-02 | down | Ni2+-Co2+ transporter transition metal uptake transporter |
| MSTRG.12825 | MSTRG.12825.1 | -1.221 | 2.07E-02 | down | hypothetical protein BBA_02790 |
| BBA_05143 | XM_008600240.1 | -1.220 | 1.65E-02 | down | hypothetical protein BBA_09131 |
| BBA_08971 | XM_008604068.1 | -1.219 | 2.52E-02 | down | hypothetical protein BBA_06776 |
| BBA_09967 | XM_008605064.1 | -1.218 | 2.06E-02 | down | -- |
| BBA_04064 | XM_008599161.1 | -1.213 | 1.87E-02 | down | hypothetical protein BBA_00152 |
| BBA_05666 | XM_008600763.1 | -1.208 | 3.39E-02 | down | endo-N-acetyl-beta-D-glucosaminidase precursor |
| BBA_09981 | XM_008605078.1 | -1.208 | 2.39E-02 | down | -- |
| BBA_09477 | XM_008604574.1 | -1.208 | 3.69E-02 | down | hypothetical protein BBA_00641 |
| MSTRG.543 | MSTRG.543.1 | -1.207 | 2.01E-02 | down | amino acid permease family protein |
| BBA_06348 | XM_008601445.1 | -1.206 | 1.80E-02 | down | uracil permease |
| BBA_06862 | XM_008601959.1 | -1.200 | 2.80E-02 | down | extracellular dipeptidyl-peptidase Dpp4 |
| BBA_04044 | XM_008599141.1 | -1.199 | 2.18E-02 | down | transcriptional regulatory protein pro-1 |
| MSTRG.13737 | MSTRG.13737.1 | -1.198 | 3.42E-02 | down | hypothetical protein BBA_08646 |
| BBA_03456 | XM_008598553.1 | -1.198 | 1.86E-02 | down | Cytochrome P450 CYP504B10 |
| BBA_02775 | XM_008597872.1 | -1.198 | 2.44E-02 | down | hypothetical protein BBA_04174 |
| BBA_00790 | XM_008595887.1 | -1.198 | 3.77E-02 | down | endonuclease/Exonuclease/phosphatase protein |
| BBA_09811 | XM_008604908.1 | -1.190 | 2.87E-02 | down | eukaryotic translation initiation factor 3 subunit G |
| BBA_04899 | XM_008599996.1 | -1.188 | 2.42E-02 | down | SH3 domain-containing protein |
| BBA_09540 | XM_008604637.1 | -1.187 | 2.34E-02 | down | NADPH oxidase |
| BBA_01010 | XM_008596107.1 | -1.186 | 2.86E-02 | down | hypothetical protein BBA_01211 |
| BBA_07478 | XM_008602575.1 | -1.181 | 3.13E-02 | down | thioester reductase domain-containing protein |
| BBA_07807 | XM_008602904.1 | -1.179 | 2.15E-02 | down | cytosol Mn-superoxide dismutase |
| MSTRG.12261 | MSTRG.12261.1 | -1.178 | 2.95E-02 | down | C6 zinc finger domain protein |
| MSTRG.1749 | MSTRG.1749.1 | -1.176 | 2.76E-02 | down | carboxylesterase-like protein |
| BBA_07381 | XM_008602478.1 | -1.175 | 4.88E-02 | down | hypothetical protein BBA_01561 |
| BBA_09706 | XM_008604803.1 | -1.175 | 2.24E-02 | down | glutamate carboxypeptidase |
| BBA_03285 | XM_008598382.1 | -1.175 | 2.34E-02 | down | endoribonuclease L-PSP |
| MSTRG.7941 | MSTRG.7941.1 | -1.170 | 3.04E-02 | down | arginine-tRNA-protein transferase |
| BBA_02534 | XM_008597631.1 | -1.168 | 2.30E-02 | down | PAN domain containing protein |
| BBA_09634 | XM_008604731.1 | -1.166 | 2.39E-02 | down | gamma-glutamyltranspeptidase |
| MSTRG.838 | MSTRG.838.1 | -1.166 | 3.35E-02 | down | hypothetical protein BBA_01337 |
| MSTRG.9222 | MSTRG.9222.1 | -1.166 | 3.05E-02 | down | integral membrane protein PTH11 |
| BBA_02413 | XM_008597510.1 | -1.166 | 3.88E-02 | down | Cytochrome P450 CYP542B1 |
| BBA_05675 | XM_008600772.1 | -1.165 | 2.83E-02 | down | N,O-diacetyl muramidase, putative |
| BBA_08435 | XM_008603532.1 | -1.164 | 3.17E-02 | down | agmatine deiminase |
| BBA_02876 | XM_008597973.1 | -1.163 | 2.75E-02 | down | polyol transporter 5 |
| BBA_04886 | XM_008599983.1 | -1.163 | 4.06E-02 | down | hypothetical protein BBA_01077 |
| BBA_01243 | XM_008596340.1 | -1.160 | 4.01E-02 | down | XAP5 domain-containing protein |
| BBA_04152 | XM_008599249.1 | -1.156 | 2.57E-02 | down | phospholipid methyltransferase |
| MSTRG.897 | MSTRG.897.1 | -1.153 | 3.76E-02 | down | MFS allantoate transporter, putative |
| BBA_04789 | XM_008599886.1 | -1.144 | 4.60E-02 | down | haloacid dehalogenase-like hydrolase |
| BBA_00399 | XM_008595496.1 | -1.135 | 4.84E-02 | down | hypothetical protein BBA_09141 |
| MSTRG.12593 | MSTRG.12593.1 | -1.135 | 4.23E-02 | down | -- |
| BBA_08603 | XM_008603700.1 | -1.134 | 3.60E-02 | down | vegetative incompatibility protein HET-E-1 |
| BBA_10180 | XM_008605277.1 | -1.134 | 3.93E-02 | down | hypothetical protein BBA_01861 |
| BBA_09284 | XM_008604381.1 | -1.133 | 4.32E-02 | down | hypothetical protein BBA_01585 |
| BBA_09372 | XM_008604469.1 | -1.133 | 2.95E-02 | down | Cytochrome P450 CYP5099A1 |
| BBA_07878 | XM_008602975.1 | -1.131 | 3.34E-02 | down | DUF218 domain-containing protein |
| MSTRG.11474 | MSTRG.11474.1 | -1.126 | 3.74E-02 | down | pheromone-regulated membrane protein 6 |
| MSTRG.12096 | MSTRG.12096.2 | -1.124 | 4.19E-02 | down | prefoldin subunit |
| BBA_05507 | XM_008600604.1 | -1.117 | 4.68E-02 | down | -- |
| MSTRG.10925 | MSTRG.10925.1 | -1.116 | 3.57E-02 | down | eukaryotic aspartyl protease |
| BBA_07661 | XM_008602758.1 | -1.116 | 4.39E-02 | down | short-chain dehydrogenase |
| MSTRG.13053 | MSTRG.13053.1 | -1.116 | 4.23E-02 | down | hypothetical protein BBA_03023 |
| MSTRG.986 | MSTRG.986.1 | -1.110 | 4.54E-02 | down | hypothetical protein BBA_10099 |
| BBA_01162 | XM_008596259.1 | -1.108 | 4.66E-02 | down | centromere binding protein B |
| BBA_01930 | XM_008597027.1 | -1.104 | 3.68E-02 | down | HD domain-containing protein |
| BBA_01327 | XM_008596424.1 | -1.096 | 4.49E-02 | down | tripeptidyl peptidase SED3 |
| BBA_09982 | XM_008605079.1 | -1.094 | 4.19E-02 | down | nonribosomal peptide synthase |
| BBA_06000 | XM_008601097.1 | -1.094 | 4.06E-02 | down | peptidase S1 and S6, chymotrypsin/Hap |
| BBA_02629 | XM_008597726.1 | -1.094 | 4.39E-02 | down | pyruvate decarboxylase |
| MSTRG.8325 | MSTRG.8325.1 | -1.091 | 4.94E-02 | down | ferric-chelate reductase |
| BBA_05337 | XM_008600434.1 | -1.087 | 4.04E-02 | down | heavy-metal-associated domain-containing protein |
| BBA_01528 | XM_008596625.1 | -1.082 | 4.74E-02 | down | frequency clock protein |
| BBA_06416 | XM_008601513.1 | -1.079 | 4.90E-02 | down | cupin 2 domain-containing protein |
| MSTRG.2340 | MSTRG.2340.1 | -1.078 | 4.30E-02 | down | dipeptidyl peptidase |
| MSTRG.1868 | MSTRG.1868.1 | -1.078 | 4.88E-02 | down | Cupredoxin-like protein |
| MSTRG.1937 | MSTRG.1937.1 | -1.078 | 4.99E-02 | down | -- |
| BBA_02530 | XM_008597627.1 | -1.077 | 4.39E-02 | down | integral membrane protein |
| BBA_02783 | XM_008597880.1 | -1.076 | 4.27E-02 | down | S-adenosylmethionine:diacylglycerol 3-amino-3-carboxypropyl transferase btaA |
| BBA_04372 | XM_008599469.1 | -1.076 | 4.88E-02 | down | glucose-repressible protein |
| BBA_09471 | XM_008604568.1 | -1.072 | 4.74E-02 | down | magnesium and cobalt transporter CorA |
| BBA_04201 | XM_008599298.1 | -1.072 | 4.42E-02 | down | family S53 protease |
| BBA_03957 | XM_008599054.1 | -1.072 | 4.39E-02 | down | -- |
| BBA_05637 | XM_008600734.1 | -1.068 | 4.94E-02 | down | hypothetical protein BBA_02871 |
| BBA_04334 | XM_008599431.1 | -1.067 | 4.83E-02 | down | RBP protein |
| MSTRG.6406 | MSTRG.6406.1 | -1.062 | 4.88E-02 | down | hypothetical protein BBA_07813 |
| BBA_00600 | XM_008595697.1 | -1.060 | 4.74E-02 | down | ATP-citrate synthase subunit 1 |
| BBA_03054 | XM_008598151.1 | -1.060 | 4.88E-02 | down | metallopeptidase family M24 |
| MSTRG.5285 | MSTRG.5285.1 | -1.058 | 4.88E-02 | down | GPI anchored protein, putative |
| BBA_02720 | XM_008597817.1 | 1.064 | 4.90E-02 | up | glycoside hydrolase family 16 |
| BBA_04958 | XM_008600055.1 | 1.068 | 4.74E-02 | up | RadR putative transcriptional regulator |
| MSTRG.1265 | MSTRG.1265.1 | 1.068 | 4.78E-02 | up | actin filament organization protein |
| BBA_08197 | XM_008603294.1 | 1.074 | 4.98E-02 | up | 2-nitropropane dioxygenase |
| BBA_07842 | XM_008602939.1 | 1.075 | 4.35E-02 | up | cyanide hydratase |
| BBA_02053 | XM_008597150.1 | 1.079 | 4.99E-02 | up | cation efflux family protein |
| BBA_02231 | XM_008597328.1 | 1.079 | 4.42E-02 | up | hypothetical protein BBA_02231 |
| BBA_02338 | XM_008597435.1 | 1.085 | 4.60E-02 | up | hypothetical protein BBA_02338 |
| BBA_02833 | XM_008597930.1 | 1.085 | 4.06E-02 | up | phosphoenolpyruvate carboxykinase |
| BBA_01653 | XM_008596750.1 | 1.087 | 4.80E-02 | up | DUF1275 domain protein |
| MSTRG.120 | MSTRG.120.1 | 1.087 | 4.05E-02 | up | hypothetical protein OCS_01881 |
| BBA_02428 | XM_008597525.1 | 1.090 | 4.27E-02 | up | Cytochrome P450 CYP52G8 |
| BBA_02396 | XM_008597493.1 | 1.103 | 3.83E-02 | up | NADPH-dependent FMN reductase |
| BBA_02056 | XM_008597153.1 | 1.106 | 4.45E-02 | up | YT521-B-like splicing factor |
| BBA_02435 | XM_008597532.1 | 1.108 | 4.55E-02 | up | peptidase C14 |
| BBA_07319 | XM_008602416.1 | 1.118 | 3.88E-02 | up | hypothetical protein BBA_07319 |
| BBA_09070 | XM_008604167.1 | 1.120 | 3.23E-02 | up | hypothetical protein BBA_09070 |
| BBA_03446 | XM_008598543.1 | 1.121 | 4.39E-02 | up | MFS transporter |
| BBA_01953 | XM_008597050.1 | 1.128 | 4.19E-02 | up | DUF962 domain-containing protein |
| BBA_02505 | XM_008597602.1 | 1.130 | 3.93E-02 | up | homeobox domain-containing protein |
| BBA_09181 | XM_008604278.1 | 1.131 | 3.87E-02 | up | chalcone-flavanone isomerase |
| BBA_06849 | XM_008601946.1 | 1.133 | 3.30E-02 | up | thermotolerance protein |
| BBA_01825 | XM_008596922.1 | 1.135 | 3.72E-02 | up | siderochrome-iron transporter MirB |
| BBA_02945 | XM_008598042.1 | 1.136 | 4.04E-02 | up | LysM domain-containing protein |
| MSTRG.4363 | MSTRG.4363.1 | 1.137 | 3.30E-02 | up | homogentisate 1,2-dioxygenase |
| MSTRG.10487 | MSTRG.10487.1 | 1.140 | 4.35E-02 | up | NADP-dependent alcohol dehydrogenase |
| BBA_09697 | XM_008604794.1 | 1.142 | 4.00E-02 | up | C2H2 transcription factor |
| BBA_01415 | XM_008596512.1 | 1.142 | 3.21E-02 | up | hypothetical protein BBA_01415 |
| MSTRG.4412 | MSTRG.4412.1 | 1.146 | 2.74E-02 | up | LysM domain-containing protein |
| BBA_06872 | XM_008601969.1 | 1.147 | 2.87E-02 | up | aspartyl-tRNA synthetase |
| BBA_03901 | XM_008598998.1 | 1.149 | 3.88E-02 | up | zinc finger protein 1 |
| BBA_04613 | XM_008599710.1 | 1.154 | 2.81E-02 | up | pyridoxamine phosphate oxidase |
| BBA_06991 | XM_008602088.1 | 1.167 | 4.07E-02 | up | alcohol dehydrogenase GroES-like domain-containing protein |
| MSTRG.4846 | MSTRG.4846.2 | 1.171 | 2.42E-02 | up | NAPE-hydrolyzing phospholipase D |
| BBA_09551 | XM_008604648.1 | 1.173 | 2.53E-02 | up | C6 transcription factor, putative |
| MSTRG.733 | MSTRG.733.1 | 1.175 | 2.18E-02 | up | hypothetical protein BBA_00531 |
| MSTRG.2906 | MSTRG.2906.1 | 1.181 | 2.43E-02 | up | -- |
| MSTRG.5332 | MSTRG.5332.1 | 1.181 | 2.16E-02 | up | O-methyltransferase family protein |
| BBA_07836 | XM_008602933.1 | 1.183 | 3.53E-02 | up | hypothetical protein BBA_07836 |
| BBA_07420 | XM_008602517.1 | 1.187 | 2.84E-02 | up | polyketide synthase, putative |
| BBA_08424 | XM_008603521.1 | 1.189 | 2.07E-02 | up | nonribosomal peptide synthase, putative |
| BBA_04262 | XM_008599359.1 | 1.197 | 2.28E-02 | up | hypothetical protein BBA_04262 |
| BBA_02808 | XM_008597905.1 | 1.197 | 2.68E-02 | up | ABC1 family protein |
| BBA_02731 | XM_008597828.1 | 1.198 | 1.99E-02 | up | hypothetical protein BBA_02731 |
| BBA_09786 | XM_008604883.1 | 1.200 | 3.35E-02 | up | nitrilotriacetate monooxygenase component B |
| MSTRG.6534 | MSTRG.6534.1 | 1.201 | 3.06E-02 | up | hypothetical protein BBAD15_g5487 |
| BBA_07721 | XM_008602818.1 | 1.201 | 2.09E-02 | up | phosphotransferase enzyme family protein |
| BBA_02558 | XM_008597655.1 | 1.201 | 2.78E-02 | up | ferulic acid esterase (FaeA) |
| BBA_03924 | XM_008599021.1 | 1.202 | 4.13E-02 | up | hypothetical protein BBA_03924 |
| BBA_08880 | XM_008603977.1 | 1.204 | 2.65E-02 | up | apolipoprotein A-I binding protein |
| BBA_01232 | XM_008596329.1 | 1.205 | 2.38E-02 | up | IKI3 family protein |
| BBA_00764 | XM_008595861.1 | 1.205 | 3.04E-02 | up | ubiquitin-conjugating enzyme E2 |
| BBA_05952 | XM_008601049.1 | 1.206 | 3.34E-02 | up | beta-mannosidase |
| BBA_03332 | XM_008598429.1 | 1.208 | 2.10E-02 | up | PRELI-like family protein |
| BBA_07387 | XM_008602484.1 | 1.215 | 1.70E-02 | up | general amidase GmdB |
| BBA_09084 | XM_008604181.1 | 1.217 | 2.62E-02 | up | actin filament organization protein |
| BBA_06555 | XM_008601652.1 | 1.217 | 3.16E-02 | up | 2-nitropropane dioxygenase |
| BBA_03703 | XM_008598800.1 | 1.220 | 1.65E-02 | up | amidase-like protein |
| MSTRG.13265 | MSTRG.13265.1 | 1.221 | 1.64E-02 | up | restless-like transposase |
| MSTRG.14151 | MSTRG.14151.1 | 1.225 | 1.75E-02 | up | hypothetical protein VFPPC_18138 |
| BBA_07748 | XM_008602845.1 | 1.230 | 1.78E-02 | up | ABC transporter |
| MSTRG.3120 | MSTRG.3120.1 | 1.235 | 1.49E-02 | up | chitinase-like protein |
| BBA_02404 | XM_008597501.1 | 1.235 | 2.48E-02 | up | Putative Zn(II)2Cys6 transcription factor |
| BBA_07868 | XM_008602965.1 | 1.236 | 3.79E-02 | up | hypothetical protein BBA_07868 |
| BBA_05069 | XM_008600166.1 | 1.236 | 4.65E-02 | up | hypothetical protein BBA_05069 |
| BBA_01298 | XM_008596395.1 | 1.238 | 1.48E-02 | up | monocarboxylate permease-like protein |
| MSTRG.12457 | MSTRG.12457.1 | 1.239 | 3.76E-02 | up | -- |
| BBA_05606 | XM_008600703.1 | 1.239 | 1.38E-02 | up | short chain dehydrogenase |
| BBA_02913 | XM_008598010.1 | 1.240 | 4.17E-02 | up | methyltransferase domain-containing protein |
| BBA_06070 | XM_008601167.1 | 1.248 | 3.36E-02 | up | ABC multidrug transporter |
| BBA_05891 | XM_008600988.1 | 1.250 | 1.42E-02 | up | hypothetical protein BBA_05891 |
| BBA_04941 | XM_008600038.1 | 1.253 | 1.25E-02 | up | radH flavin-dependent halogenase |
| BBA_07988 | XM_008603085.1 | 1.254 | 1.25E-02 | up | hypothetical protein BBA_07988 |
| BBA_06557 | XM_008601654.1 | 1.256 | 1.80E-02 | up | FAD binding domain protein |
| BBA_03928 | XM_008599025.1 | 1.257 | 1.48E-02 | up | hypothetical protein BBA_03928 |
| BBA_03138 | XM_008598235.1 | 1.258 | 1.19E-02 | up | LysM domain-containing protein |
| MSTRG.13111 | MSTRG.13111.1 | 1.262 | 1.91E-02 | up | -- |
| BBA_09641 | XM_008604738.1 | 1.262 | 1.31E-02 | up | Fungal Zn binuclear cluster domain containing protein |
| BBA_08733 | XM_008603830.1 | 1.266 | 1.35E-02 | up | allantoicase-like protein |
| BBA_02658 | XM_008597755.1 | 1.267 | 4.84E-02 | up | hypothetical protein BBA_02658 |
| BBA_00207 | XM_008595304.1 | 1.269 | 1.21E-02 | up | cation diffusion facilitator family transporter |
| BBA_09617 | XM_008604714.1 | 1.270 | 3.58E-02 | up | NFX1-type zinc finger-containing protein 1 |
| BBA_00080 | XM_008595177.1 | 1.273 | 1.89E-02 | up | hypothetical protein BBA_00080 |
| BBA_08220 | XM_008603317.1 | 1.275 | 1.05E-02 | up | trichothecene 3-O-acetyltransferase |
| BBA_06154 | XM_008601251.1 | 1.278 | 3.22E-02 | up | triacylglycerol lipase |
| BBA_07533 | XM_008602630.1 | 1.279 | 1.79E-02 | up | NADP-dependent alcohol dehydrogenase |
| BBA_07304 | XM_008602401.1 | 1.285 | 2.02E-02 | up | malate dehydrogenase, putative |
| MSTRG.12421 | MSTRG.12421.1 | 1.288 | 1.12E-02 | up | apolipoprotein A-I binding protein |
| BBA_03594 | XM_008598691.1 | 1.296 | 1.98E-02 | up | Thioredoxin-like protein |
| BBA_07054 | XM_008602151.1 | 1.297 | 2.22E-02 | up | C6 zinc finger domain-containing protein |
| MSTRG.10578 | MSTRG.10578.1 | 1.299 | 9.93E-03 | up | stress responsive A/B Barrel domain-containing protein |
| BBA_04327 | XM_008599424.1 | 1.299 | 3.17E-02 | up | amino acid adenylation domain protein |
| MSTRG.14287 | MSTRG.14287.1 | 1.300 | 3.67E-02 | up | Retrovirus-related Pol polyprotein from type-1 retrotransposable element R1 |
| BBA_02968 | XM_008598065.1 | 1.300 | 1.60E-02 | up | regulator of G protein signaling |
| BBA_01713 | XM_008596810.1 | 1.309 | 1.15E-02 | up | pyridine nucleotide-disulfide oxidoreductase, putative |
| BBA_09600 | XM_008604697.1 | 1.309 | 1.26E-02 | up | FluG domain-containing protein |
| BBA_08717 | XM_008603814.1 | 1.312 | 1.29E-02 | up | hypothetical protein BBA_08717 |
| BBA_00071 | XM_008595168.1 | 1.315 | 1.76E-02 | up | nitrate assimilation regulatory protein nirA |
| BBA_08264 | XM_008603361.1 | 1.317 | 1.07E-02 | up | 2-amino-3-carboxymuconate-6-semialdehyde decarboxylase |
| BBA_04807 | XM_008599904.1 | 1.319 | 2.28E-02 | up | SPX domain-containing protein |
| BBA_00807 | XM_008595904.1 | 1.321 | 1.09E-02 | up | glucose repressible protein Grg1 |
| BBA_04725 | XM_008599822.1 | 1.321 | 9.51E-03 | up | eukaryotic and archaeal DNA primase |
| BBA_06386 | XM_008601483.1 | 1.322 | 1.38E-02 | up | hypothetical protein BBA_06386 |
| BBA_04703 | XM_008599800.1 | 1.324 | 3.44E-02 | up | hypothetical protein BBA_04703 |
| BBA_02608 | XM_008597705.1 | 1.325 | 1.64E-02 | up | biotrophy-associated secreted protein 2 |
| BBA_03670 | XM_008598767.1 | 1.327 | 7.00E-03 | up | MFS transporter, putative |
| MSTRG.7570 | MSTRG.7570.1 | 1.330 | 3.62E-02 | up | hypothetical protein BBA_05366 |
| BBA_01863 | XM_008596960.1 | 1.331 | 7.47E-03 | up | -- |
| BBA_06121 | XM_008601218.1 | 1.338 | 8.58E-03 | up | C2H2 finger domain-containing protein |
| MSTRG.6307 | MSTRG.6307.1 | 1.351 | 1.02E-02 | up | hypothetical protein BBAD15_g5650 |
| BBA_06361 | XM_008601458.1 | 1.351 | 1.12E-02 | up | dienelactone hydrolase family protein |
| BBA_05366 | XM_008600463.1 | 1.352 | 1.71E-02 | up | hypothetical protein BBA_05366 |
| BBA_04696 | XM_008599793.1 | 1.354 | 6.75E-03 | up | short chain dehydrogenase/reductase |
| BBA_07952 | XM_008603049.1 | 1.354 | 3.70E-02 | up | cellulase-like protein |
| BBA_06336 | XM_008601433.1 | 1.358 | 5.72E-03 | up | hexose transporter-like protein |
| BBA_07212 | XM_008602309.1 | 1.362 | 8.40E-03 | up | phospholipase A2 protein family |
| BBA_03078 | XM_008598175.1 | 1.365 | 5.35E-03 | up | Cytochrome P450 CYP68N1 |
| BBA_08222 | XM_008603319.1 | 1.366 | 5.13E-03 | up | nonribosomal peptide synthase, putative |
| BBA_08439 | XM_008603536.1 | 1.366 | 6.85E-03 | up | zinc-binding dehydrogenase |
| MSTRG.12229 | MSTRG.12229.1 | 1.367 | 2.19E-02 | up | -- |
| BBA_09356 | XM_008604453.1 | 1.368 | 9.51E-03 | up | Major Facilitator Superfamily protein |
| BBA_08186 | XM_008603283.1 | 1.382 | 7.71E-03 | up | integral membrane protein |
| MSTRG.121 | MSTRG.121.1 | 1.386 | 4.26E-03 | up | hypothetical protein OCS_01881 |
| MSTRG.9628 | MSTRG.9628.1 | 1.386 | 9.31E-03 | up | -- |
| BBA_02936 | XM_008598033.1 | 1.389 | 8.83E-03 | up | peptidase family protein |
| BBA_02735 | XM_008597832.1 | 1.390 | 1.04E-02 | up | hypothetical protein BBA_02735 |
| BBA_07303 | XM_008602400.1 | 1.392 | 7.76E-03 | up | hypothetical protein BBA_07303 |
| BBA_05051 | XM_008600148.1 | 1.392 | 6.04E-03 | up | MIP family channel protein |
| BBA_04500 | XM_008599597.1 | 1.398 | 6.01E-03 | up | Glycoside hydrolase, family 31 |
| BBA_00696 | XM_008595793.1 | 1.398 | 6.48E-03 | up | hypothetical protein BBA_00696 |
| BBA_03472 | XM_008598569.1 | 1.398 | 7.26E-03 | up | Ankyrin repeat protein |
| BBA_08458 | XM_008603555.1 | 1.402 | 5.72E-03 | up | two component transcriptional regulator, LuxR family |
| BBA_08505 | XM_008603602.1 | 1.408 | 1.12E-02 | up | subtilase-like protein |
| BBA_02340 | XM_008597437.1 | 1.409 | 3.85E-03 | up | hypothetical protein BBA_02340 |
| BBA_08214 | XM_008603311.1 | 1.426 | 6.37E-03 | up | cell wall glucanosyltransferase Mwg1 |
| BBA_06971 | XM_008602068.1 | 1.432 | 3.34E-03 | up | methyltransferase-like protein |
| BBA_08402 | XM_008603499.1 | 1.435 | 2.90E-03 | up | monooxygenase-like protein |
| BBA_00792 | XM_008595889.1 | 1.436 | 2.80E-03 | up | beta-1, 3 exoglucanase precursor |
| BBA_10103 | XM_008605200.1 | 1.437 | 3.81E-03 | up | ATP synthase beta chain, mitochondrial |
| BBA_05833 | XM_008600930.1 | 1.437 | 4.45E-03 | up | major facilitator superfamily transporter |
| BBA_08224 | XM_008603321.1 | 1.440 | 3.12E-03 | up | hypothetical protein BBA_08224 |
| BBA_10115 | XM_008605212.1 | 1.444 | 7.37E-03 | up | endo alpha-1,4 polygalactosaminidase precursor |
| BBA_10358 | XM_008605455.1 | 1.445 | 4.03E-03 | up | hypothetical protein BBA_10358 |
| BBA_02736 | XM_008597833.1 | 1.445 | 3.59E-03 | up | hypothetical protein BBA_02736 |
| BBA_06824 | XM_008601921.1 | 1.446 | 2.89E-02 | up | Cytochrome P450 CYP51F2 |
| BBA_06058 | XM_008601155.1 | 1.447 | 5.81E-03 | up | esterase-like protein |
| BBA_01820 | XM_008596917.1 | 1.447 | 1.89E-02 | up | voltage-gated hydrogen channel 1 |
| BBA_07869 | XM_008602966.1 | 1.453 | 5.59E-03 | up | nudix domain containing protein |
| BBA_08176 | XM_008603273.1 | 1.454 | 4.79E-02 | up | mitochondrial CorA family metal ion transporter |
| BBA_04406 | XM_008599503.1 | 1.455 | 2.85E-03 | up | hypothetical protein BBA_04406 |
| MSTRG.3793 | MSTRG.3793.1 | 1.455 | 3.23E-03 | up | hypothetical protein BBAD15_g8818 |
| BBA_03682 | XM_008598779.1 | 1.457 | 5.86E-03 | up | isoflavone reductase family protein |
| BBA_06338 | XM_008601435.1 | 1.458 | 3.07E-03 | up | hypothetical protein BBA_06338 |
| BBA_05376 | XM_008600473.1 | 1.458 | 2.36E-03 | up | alpha-amylase A type-3 |
| BBA_08974 | XM_008604071.1 | 1.460 | 5.58E-03 | up | antifungal protein |
| BBA_05123 | XM_008600220.1 | 1.461 | 4.40E-03 | up | Putative Zn(II)2Cys6 transcription factor |
| BBA_04668 | XM_008599765.1 | 1.467 | 9.19E-03 | up | phenylacrylic acid decarboxylase, putative |
| MSTRG.260 | MSTRG.260.1 | 1.470 | 3.17E-02 | up | -- |
| BBA_04973 | XM_008600070.1 | 1.470 | 2.15E-03 | up | MFS transporter |
| BBA_09458 | XM_008604555.1 | 1.471 | 2.07E-02 | up | gag protein |
| BBA_09931 | XM_008605028.1 | 1.472 | 1.31E-02 | up | hypothetical protein BBA_09931 |
| BBA_07667 | XM_008602764.1 | 1.473 | 1.79E-02 | up | reverse transcriptase |
| MSTRG.3683 | MSTRG.3683.1 | 1.478 | 5.90E-03 | up | -- |
| BBA_05859 | XM_008600956.1 | 1.479 | 3.85E-03 | up | hypothetical protein BBA_05859 |
| BBA_08180 | XM_008603277.1 | 1.480 | 1.91E-03 | up | MFS multidrug resistance transporter, putative |
| BBA_06595 | XM_008601692.1 | 1.483 | 3.30E-02 | up | hypothetical protein UCP014753 |
| BBA_06726 | XM_008601823.1 | 1.490 | 3.23E-02 | up | Cytochrome P450 CYP625A1 |
| BBA_00732 | XM_008595829.1 | 1.501 | 2.02E-02 | up | apoptosis-inducing factor, putative |
| BBA_05939 | XM_008601036.1 | 1.502 | 2.57E-02 | up | fungal specific transcription factor |
| BBA_02942 | XM_008598039.1 | 1.503 | 6.46E-03 | up | LysM domain containing protein |
| BBA_07211 | XM_008602308.1 | 1.504 | 1.67E-03 | up | hypothetical protein BBA_07211 |
| BBA_01781 | XM_008596878.1 | 1.507 | 1.50E-03 | up | ABC-2 type transporter |
| BBA_08183 | XM_008603280.1 | 1.508 | 1.46E-03 | up | laccase 2 |
| BBA_07782 | XM_008602879.1 | 1.510 | 1.46E-03 | up | hypothetical protein BBA_07782 |
| BBA_04061 | XM_008599158.1 | 1.512 | 1.42E-03 | up | tyrosinase 2 |
| BBA_01654 | XM_008596751.1 | 1.515 | 1.42E-03 | up | oxalate decarboxylase family bicupin |
| MSTRG.2615 | MSTRG.2615.1 | 1.515 | 1.37E-03 | up | -- |
| BBA_06453 | XM_008601550.1 | 1.516 | 1.04E-02 | up | hypothetical protein BBA_06453 |
| BBA_00379 | XM_008595476.1 | 1.518 | 2.47E-03 | up | TspO/MBR family protein |
| BBA_06207 | XM_008601304.1 | 1.521 | 2.42E-03 | up | hypothetical protein BBA_06207 |
| BBA_07141 | XM_008602238.1 | 1.522 | 2.87E-03 | up | MFS transporter, putative |
| BBA_07534 | XM_008602631.1 | 1.524 | 1.28E-03 | up | hypothetical protein BBA_07534 |
| BBA_06090 | XM_008601187.1 | 1.527 | 5.03E-03 | up | aminodeoxychorismate lyase |
| BBA_08469 | XM_008603566.1 | 1.533 | 2.18E-02 | up | amidohydrolase-like protein |
| BBA_02303 | XM_008597400.1 | 1.542 | 1.13E-03 | up | coproporphyrinogen III oxidase |
| BBA_01737 | XM_008596834.1 | 1.544 | 4.87E-03 | up | F-box protein |
| BBA_02311 | XM_008597408.1 | 1.547 | 1.08E-03 | up | Cu/Zn superoxide dismutase |
| BBA_07094 | XM_008602191.1 | 1.549 | 2.98E-03 | up | mitochondrial phosphate carrier protein |
| BBA_02140 | XM_008597237.1 | 1.549 | 9.99E-04 | up | hypothetical protein BBA_02140 |
| MSTRG.12820 | MSTRG.12820.1 | 1.550 | 2.34E-03 | up | hypothetical protein BBA_09903 |
| BBA_01236 | XM_008596333.1 | 1.552 | 1.22E-03 | up | major facilitator superfamily transporter |
| BBA_05612 | XM_008600709.1 | 1.552 | 6.21E-03 | up | beta-mannosidase A |
| BBA_01231 | XM_008596328.1 | 1.553 | 1.72E-03 | up | hypothetical protein BBA_01231 |
| MSTRG.11461 | MSTRG.11461.1 | 1.555 | 6.07E-03 | up | -- |
| BBA_03815 | XM_008598912.1 | 1.557 | 4.89E-02 | up | formate/nitrate family transporter |
| BBA_07518 | XM_008602615.1 | 1.558 | 1.22E-03 | up | WD repeat domain-containing protein |
| BBA_04935 | XM_008600032.1 | 1.559 | 1.13E-03 | up | hypothetical protein BBA_04935 |
| BBA_07781 | XM_008602878.1 | 1.560 | 2.63E-03 | up | F-box and wd40 domain protein |
| BBA_04778 | XM_008599875.1 | 1.566 | 2.31E-03 | up | transcriptional repressor TUP1 |
| MSTRG.3657 | MSTRG.3657.1 | 1.567 | 2.88E-02 | up | hypothetical protein PV08_01672 |
| MSTRG.13202 | MSTRG.13202.1 | 1.568 | 2.51E-03 | up | -- |
| BBA_02569 | XM_008597666.1 | 1.570 | 1.26E-02 | up | hypothetical protein BBA_02569 |
| BBA_05255 | XM_008600352.1 | 1.571 | 2.88E-02 | up | ankyrin repeat protein |
| BBA_06996 | XM_008602093.1 | 1.573 | 2.62E-02 | up | AMP-binding enzyme |
| MSTRG.7946 | MSTRG.7946.1 | 1.575 | 8.45E-04 | up | hypothetical protein CCM_04613 |
| BBA_07379 | XM_008602476.1 | 1.576 | 8.46E-04 | up | transferase family protein |
| BBA_09347 | XM_008604444.1 | 1.579 | 1.36E-02 | up | MFS transporter, putative |
| BBA_03436 | XM_008598533.1 | 1.580 | 7.53E-04 | up | hypothetical protein BBA_03436 |
| BBA_03953 | XM_008599050.1 | 1.580 | 4.78E-02 | up | eukaryotic aspartyl protease |
| BBA_09110 | XM_008604207.1 | 1.582 | 2.44E-03 | up | tachykinin family protein |
| BBA_03242 | XM_008598339.1 | 1.584 | 9.75E-04 | up | putative alternative NADH dehydrogenase |
| BBA_02824 | XM_008597921.1 | 1.586 | 1.57E-02 | up | short chain dehydrogenase |
| BBA_09913 | XM_008605010.1 | 1.589 | 7.91E-03 | up | SET domain-containing protein 5 |
| BBA_09985 | XM_008605082.1 | 1.591 | 2.91E-03 | up | DUF124 domain protein |
| BBA_09298 | XM_008604395.1 | 1.595 | 1.51E-03 | up | Zn(II)2Cys6 transcription factor |
| MSTRG.5748 | MSTRG.5748.1 | 1.598 | 8.66E-03 | up | -- |
| MSTRG.11685 | MSTRG.11685.1 | 1.616 | 6.70E-04 | up | Pectin lyase fold/virulence factor |
| MSTRG.13430 | MSTRG.13430.1 | 1.617 | 4.09E-02 | up | -- |
| BBA_04473 | XM_008599570.1 | 1.620 | 9.51E-03 | up | hypothetical protein BBA_04473 |
| MSTRG.12974 | MSTRG.12974.1 | 1.629 | 1.83E-03 | up | hypothetical protein Micbo1qcDRAFT_168462 |
| BBA_06806 | XM_008601903.1 | 1.631 | 1.44E-03 | up | multidrug resistance protein MDR, putative |
| BBA_01780 | XM_008596877.1 | 1.633 | 1.14E-03 | up | putative xylanase 1 |
| BBA_10215 | XM_008605312.1 | 1.638 | 8.58E-03 | up | hypothetical protein BBA_10215 |
| BBA_00423 | XM_008595520.1 | 1.641 | 2.24E-03 | up | multidrug and toxin extrusion protein |
| BBA_06867 | XM_008601964.1 | 1.643 | 8.11E-04 | up | major facilitator superfamily transporter |
| MSTRG.12540 | MSTRG.12540.1 | 1.644 | 7.53E-04 | up | antifungal protein |
| BBA_03058 | XM_008598155.1 | 1.649 | 6.56E-04 | up | DUF1348 domain protein |
| MSTRG.12957 | MSTRG.12957.1 | 1.649 | 2.87E-03 | up | hypothetical protein BBAD15_g11245 |
| MSTRG.12224 | MSTRG.12224.1 | 1.652 | 8.84E-04 | up | -- |
| BBA_09464 | XM_008604561.1 | 1.655 | 3.15E-02 | up | hypothetical protein BBA_09464 |
| BBA_02214 | XM_008597311.1 | 1.658 | 6.95E-04 | up | subtilase-like protein |
| BBA_05929 | XM_008601026.1 | 1.659 | 3.59E-04 | up | WSC domain-containing protein |
| MSTRG.11166 | MSTRG.11166.1 | 1.660 | 3.84E-03 | up | family S53 protease-like protein |
| MSTRG.2102 | MSTRG.2102.1 | 1.665 | 2.02E-02 | up | IST1 protein [Beauveria bassiana D1-5] |
| BBA_04702 | XM_008599799.1 | 1.666 | 7.52E-04 | up | C6 transcription factor |
| BBA_09113 | XM_008604210.1 | 1.667 | 1.57E-02 | up | epoxide hydrolase |
| BBA_01971 | XM_008597068.1 | 1.668 | 9.14E-04 | up | LPS glycosyltransferase, putative |
| BBA_00828 | XM_008595925.1 | 1.672 | 6.73E-04 | up | integral membrane family protein |
| MSTRG.2147 | MSTRG.2147.1 | 1.672 | 3.00E-02 | up | -- |
| BBA_04295 | XM_008599392.1 | 1.680 | 4.03E-03 | up | C2 domain-containing protein |
| BBA_08688 | XM_008603785.1 | 1.680 | 5.34E-04 | up | heat shock protein 30 |
| BBA_07402 | XM_008602499.1 | 1.682 | 7.16E-04 | up | kelch repeat protein |
| BBA_02230 | XM_008597327.1 | 1.693 | 2.59E-04 | up | chitinase-like protein |
| BBA_02864 | XM_008597961.1 | 1.702 | 5.89E-04 | up | heavy metal translocating P-type ATPase |
| BBA_10169 | XM_008605266.1 | 1.702 | 1.22E-03 | up | ATPase protein |
| MSTRG.10016 | MSTRG.10016.1 | 1.707 | 3.78E-02 | up | -- |
| BBA_03824 | XM_008598921.1 | 1.708 | 2.90E-04 | up | CFEM domain-containing protein |
| BBA_04252 | XM_008599349.1 | 1.709 | 4.80E-04 | up | F1F0-ATP synthase regulatory factor Stf2 |
| BBA_01748 | XM_008596845.1 | 1.712 | 2.14E-04 | up | NADPH dehydrogenase |
| MSTRG.1636 | MSTRG.1636.2 | 1.714 | 4.73E-03 | up | hypothetical protein BBAD15_g4297 |
| BBA_01115 | XM_008596212.1 | 1.722 | 1.24E-02 | up | epoxide hydrolase 1 |
| BBA_09314 | XM_008604411.1 | 1.723 | 2.83E-02 | up | Putative Zn(II)2Cys6 transcription factor |
| BBA_04585 | XM_008599682.1 | 1.730 | 2.17E-02 | up | elongation factor-2 kinase EFK-1B isoform |
| MSTRG.13813 | MSTRG.13813.1 | 1.730 | 9.55E-04 | up | 2-oxoacid dehydrogenase acyltransferase |
| BBA_09295 | XM_008604392.1 | 1.741 | 1.66E-04 | up | glutathione-dependent formaldehyde-activating enzyme |
| BBA_06578 | XM_008601675.1 | 1.744 | 1.65E-04 | up | heterokaryon incompatibility protein |
| BBA_05483 | XM_008600580.1 | 1.746 | 1.75E-02 | up | hypothetical protein BBA_05483 |
| BBA_07944 | XM_008603041.1 | 1.748 | 1.57E-04 | up | PAF acetylhydrolase |
| MSTRG.3106 | MSTRG.3106.1 | 1.750 | 1.58E-03 | up | -- |
| BBA_10213 | XM_008605310.1 | 1.751 | 3.47E-03 | up | platelet-activating factor acetylhydrolase |
| BBA_03073 | XM_008598170.1 | 1.757 | 2.23E-04 | up | DJ-1/PfpI family protein |
| BBA_05710 | XM_008600807.1 | 1.757 | 5.11E-03 | up | hypothetical protein BBA_05710 |
| MSTRG.9177 | MSTRG.9177.1 | 1.759 | 6.96E-03 | up | -- |
| BBA_01464 | XM_008596561.1 | 1.760 | 1.24E-02 | up | Actin-like protein |
| MSTRG.9326 | MSTRG.9326.1 | 1.762 | 5.79E-04 | up | hypothetical protein BBA_06637 |
| BBA_09208 | XM_008604305.1 | 1.776 | 2.03E-02 | up | dimethylaniline monooxygenase |
| BBA_08996 | XM_008604093.1 | 1.779 | 2.07E-03 | up | NADH-cytochrome b5 reductase |
| BBA_01838 | XM_008596935.1 | 1.788 | 4.29E-03 | up | hypothetical protein BBA_01838 |
| BBA_01117 | XM_008596214.1 | 1.789 | 6.25E-04 | up | C6 zinc finger domain-containing protein |
| MSTRG.14004 | MSTRG.14004.1 | 1.790 | 4.28E-03 | up | kynurenine 3-monooxygenase, putative |
| BBA_02067 | XM_008597164.1 | 1.793 | 1.57E-02 | up | proline-specific permease, putative |
| BBA_09197 | XM_008604294.1 | 1.799 | 2.05E-04 | up | interferon-induced GTP-binding protein Mx |
| BBA_04675 | XM_008599772.1 | 1.799 | 1.15E-02 | up | hypothetical protein BBA_04675 |
| BBA_09123 | XM_008604220.1 | 1.800 | 2.01E-04 | up | multidrug resistance protein MDR, putative |
| BBA_09719 | XM_008604816.1 | 1.800 | 1.55E-04 | up | MFS toxin efflux pump (AflT) |
| MSTRG.14426 | MSTRG.14426.1 | 1.802 | 3.87E-02 | up | -- |
| BBA_08801 | XM_008603898.1 | 1.804 | 1.08E-03 | up | beta-1,6-glucanase precursor |
| BBA_09539 | XM_008604636.1 | 1.807 | 3.31E-04 | up | protein phosphatase regulator |
| BBA_09821 | XM_008604918.1 | 1.814 | 1.21E-03 | up | translation factor (SUA5) |
| BBA_09299 | XM_008604396.1 | 1.817 | 2.01E-04 | up | sarcosine oxidase |
| BBA_07886 | XM_008602983.1 | 1.818 | 1.02E-04 | up | hsp20-like protein |
| BBA_02630 | XM_008597727.1 | 1.818 | 6.95E-05 | up | bassianolide nonribosomal peptide synthetase |
| MSTRG.6417 | MSTRG.6417.1 | 1.823 | 6.67E-05 | up | hypothetical protein BBA_04559 |
| BBA_04627 | XM_008599724.1 | 1.824 | 8.21E-05 | up | ACC deaminase |
| BBA_06206 | XM_008601303.1 | 1.834 | 2.09E-02 | up | vacuolar protein sorting-associated protein 62 |
| BBA_04704 | XM_008599801.1 | 1.850 | 7.53E-04 | up | hypothetical protein BBA_04704 |
| BBA_07697 | XM_008602794.1 | 1.853 | 4.57E-03 | up | hypothetical protein BBA_07697 |
| MSTRG.3901 | MSTRG.3901.1 | 1.854 | 3.84E-04 | up | hypothetical protein G647_08909 |
| BBA_00931 | XM_008596028.1 | 1.856 | 6.44E-05 | up | 7 alpha-cephem-methoxylase, putative |
| BBA_07774 | XM_008602871.1 | 1.856 | 4.67E-04 | up | serine/threonine protein kinase |
| BBA_10364 | XM_008605459.1 | 1.861 | 9.46E-04 | up | kinesin light chain |
| BBA_07101 | XM_008602198.1 | 1.864 | 6.76E-05 | up | hypothetical protein BBA_07101 |
| BBA_02037 | XM_008597134.1 | 1.866 | 8.80E-05 | up | multicopper oxidase |
| MSTRG.10580 | MSTRG.10580.1 | 1.876 | 7.47E-03 | up | -- |
| BBA_10168 | XM_008605265.1 | 1.878 | 1.66E-04 | up | geranylgeranyl pyrophosphate synthetase |
| BBA_10351 | XM_008605448.1 | 1.878 | 4.02E-03 | up | serine/threonine protein kinase Japonica Group |
| BBA_09400 | XM_008604497.1 | 1.881 | 4.84E-04 | up | hypothetical protein BBA_09400 |
| BBA_03002 | XM_008598099.1 | 1.892 | 4.57E-05 | up | Cytochrome P450 CYP561D2P |
| BBA_02357 | XM_008597454.1 | 1.893 | 7.09E-05 | up | NADP-dependent alcohol dehydrogenase |
| BBA_04088 | XM_008599185.1 | 1.894 | 8.87E-03 | up | hypothetical protein BBA_04088 |
| MSTRG.14238 | MSTRG.14238.1 | 1.895 | 1.95E-04 | up | -- |
| BBA_05551 | XM_008600648.1 | 1.896 | 3.17E-05 | up | Beta-lactamase-type transpeptidase |
| MSTRG.9078 | MSTRG.9078.1 | 1.897 | 4.12E-04 | up | -- |
| BBA_06677 | XM_008601774.1 | 1.898 | 5.64E-04 | up | hypothetical protein BBA_06677 |
| BBA_00006 | XM_008595103.1 | 1.898 | 2.39E-04 | up | hypothetical protein BBA_00006 |
| BBA_00405 | XM_008595502.1 | 1.906 | 1.93E-02 | up | BTB/POZ domain containing protein |
| BBA_02757 | XM_008597854.1 | 1.907 | 3.26E-05 | up | aldo-keto reductase yakc |
| BBA_02114 | XM_008597211.1 | 1.908 | 3.48E-05 | up | mitochondrial phosphate carrier protein |
| BBA_08473 | XM_008603570.1 | 1.909 | 1.66E-04 | up | siderophore iron transporter mirA |
| BBA_09079 | XM_008604176.1 | 1.910 | 6.41E-05 | up | major facilitator superfamily protein |
| MSTRG.9074 | MSTRG.9074.1 | 1.910 | 5.42E-04 | up | -- |
| BBA_10214 | XM_008605311.1 | 1.920 | 2.86E-05 | up | hypothetical protein BBA_10214 |
| MSTRG.5915 | MSTRG.5915.1 | 1.921 | 5.72E-03 | up | hypothetical protein LEL_08298 |
| MSTRG.10824 | MSTRG.10824.1 | 1.923 | 2.81E-05 | up | -- |
| BBA_04833 | XM_008599930.1 | 1.926 | 1.46E-04 | up | hypothetical protein BBA_04833 |
| MSTRG.6438 | MSTRG.6438.1 | 1.929 | 1.69E-03 | up | WD domain-containing protein |
| BBA_02383 | XM_008597480.1 | 1.934 | 6.34E-04 | up | hypothetical protein BBA_02383 |
| BBA_09726 | XM_008604823.1 | 1.934 | 2.03E-05 | up | 2-dehydropantoate 2-reductase, putative |
| BBA_10100 | XM_008605197.1 | 1.936 | 7.04E-03 | up | hypothetical protein BBA_10100 |
| MSTRG.13775 | MSTRG.13775.1 | 1.944 | 1.83E-04 | up | Ankyrin repeat protein |
| BBA_06714 | XM_008601811.1 | 1.954 | 1.32E-02 | up | hypothetical protein BBA_06714 |
| BBA_00896 | XM_008595993.1 | 1.954 | 1.78E-05 | up | major facilitator superfamily transporter |
| BBA_07335 | XM_008602432.1 | 1.957 | 3.79E-05 | up | Cytochrome P450 CYP655C1 |
| MSTRG.5146 | MSTRG.5146.1 | 1.959 | 4.27E-05 | up | -- |
| MSTRG.1224 | MSTRG.1224.1 | 1.961 | 1.06E-04 | up | Isoleucyl-tRNA synthetase |
| BBA_08602 | XM_008603699.1 | 1.967 | 1.77E-05 | up | LysM domain-containing protein |
| BBA_04512 | XM_008599609.1 | 1.969 | 8.99E-05 | up | hypothetical protein BBA_04512 |
| BBA_09048 | XM_008604145.1 | 1.970 | 2.98E-05 | up | hypothetical protein BBA_09048 |
| BBA_06609 | XM_008601706.1 | 1.972 | 2.06E-03 | up | myosin-cross-reactive antigen |
| BBA_05015 | XM_008600112.1 | 1.977 | 1.50E-05 | up | multidrug resistance protein CDR1 |
| BBA_07374 | XM_008602471.1 | 1.981 | 2.41E-05 | up | hypothetical protein BBA_07374 |
| BBA_07416 | XM_008602513.1 | 1.983 | 9.47E-05 | up | glutamyl-tRNA synthetase |
| MSTRG.6408 | MSTRG.6408.1 | 1.986 | 1.44E-02 | up | -- |
| BBA_05349 | XM_008600446.1 | 1.986 | 1.28E-05 | up | fasciclin domain-containing protein |
| BBA_09164 | XM_008604261.1 | 1.990 | 2.58E-04 | up | oxidoreductase, 2OG-Fe(II) oxygenase family protein |
| MSTRG.14001 | MSTRG.14001.1 | 1.993 | 8.49E-03 | up | hypothetical protein HIM_09824 |
| MSTRG.3128 | MSTRG.3128.1 | 2.019 | 2.69E-02 | up | F-box domain containing protein |
| BBA_06217 | XM_008601314.1 | 2.026 | 1.10E-05 | up | quinone oxidoreductase |
| BBA_01908 | XM_008597005.1 | 2.026 | 9.41E-04 | up | hypothetical protein BBA_01908 |
| BBA_04746 | XM_008599843.1 | 2.034 | 8.63E-06 | up | HypA protein |
| BBA_06446 | XM_008601543.1 | 2.036 | 1.82E-05 | up | hypothetical protein BBA_06446 |
| BBA_06533 | XM_008601630.1 | 2.038 | 6.42E-06 | up | alcohol oxidase |
| BBA_07735 | XM_008602832.1 | 2.040 | 3.05E-02 | up | cytochrome c oxidase subunit Via |
| BBA_08663 | XM_008603760.1 | 2.055 | 2.24E-03 | up | hypothetical protein BBA_08663 |
| BBA_09592 | XM_008604689.1 | 2.055 | 1.55E-05 | up | major facilitator superfamily transporter |
| BBA_01001 | XM_008596098.1 | 2.059 | 6.21E-06 | up | phospholipase/carboxylesterase |
| BBA_02726 | XM_008597823.1 | 2.060 | 3.91E-04 | up | hypothetical protein BBA_02726 |
| MSTRG.13784 | MSTRG.13784.1 | 2.062 | 1.73E-02 | up | hypothetical protein BBA_09919 |
| BBA_05117 | XM_008600214.1 | 2.071 | 1.73E-02 | up | SET domain-containing protein |
| MSTRG.13413 | MSTRG.13413.1 | 2.073 | 1.21E-03 | up | hypothetical protein BBA_10216 |
| BBA_06637 | XM_008601734.1 | 2.081 | 3.86E-06 | up | hypothetical protein BBA_06637 |
| MSTRG.4577 | MSTRG.4577.1 | 2.081 | 1.04E-02 | up | transcription-coupled repair protein CSB/RAD26 |
| MSTRG.10283 | MSTRG.10283.1 | 2.083 | 2.33E-04 | up | -- |
| BBA_05309 | XM_008600406.1 | 2.085 | 1.34E-04 | up | phosphotransferase enzyme family protein |
| BBA_08934 | XM_008604031.1 | 2.091 | 3.33E-03 | up | proline dehydrogenase |
| MSTRG.7549 | MSTRG.7549.1 | 2.093 | 2.41E-02 | up | -- |
| MSTRG.12698 | MSTRG.12698.1 | 2.099 | 7.15E-04 | up | hypothetical protein BBA_09106 |
| BBA_08792 | XM_008603889.1 | 2.104 | 2.14E-05 | up | DUF300 domain protein, putative |
| BBA_03635 | XM_008598732.1 | 2.109 | 1.39E-03 | up | IDI-2 precursor |
| BBA_03668 | XM_008598765.1 | 2.114 | 5.64E-04 | up | hypothetical protein BBA_03668 |
| BBA_07009 | XM_008602106.1 | 2.123 | 3.15E-06 | up | alkaline foam protein B precursor |
| MSTRG.12029 | MSTRG.12029.1 | 2.125 | 2.04E-06 | up | LysM domain-containing protein |
| BBA_09565 | XM_008604662.1 | 2.126 | 2.59E-04 | up | hypothetical protein BBA_09565 |
| BBA_00525 | XM_008595622.1 | 2.132 | 2.34E-06 | up | GPI anchored cell wall protein |
| BBA_03411 | XM_008598508.1 | 2.137 | 8.81E-04 | up | hypothetical protein BBA_03411 |
| BBA_04905 | XM_008600002.1 | 2.148 | 2.97E-05 | up | hypothetical protein BBA_04905 |
| BBA_06382 | XM_008601479.1 | 2.149 | 3.06E-06 | up | hypothetical protein BBA_06382 |
| BBA_06869 | XM_008601966.1 | 2.153 | 4.62E-05 | up | CgERG6-2 protein |
| MSTRG.11355 | MSTRG.11355.1 | 2.153 | 1.91E-02 | up | hypothetical protein BBA_08113 |
| BBA_02751 | XM_008597848.1 | 2.155 | 4.58E-04 | up | Cytochrome P450 CYP5282A1 |
| BBA_02941 | XM_008598038.1 | 2.158 | 4.09E-06 | up | chitinase A1 |
| BBA_06215 | XM_008601312.1 | 2.162 | 2.34E-04 | up | hypothetical protein BBA_06215 |
| BBA_02602 | XM_008597699.1 | 2.173 | 1.19E-06 | up | cell wall protein |
| BBA_00297 | XM_008595394.1 | 2.183 | 1.25E-06 | up | class V chitinase, putative |
| BBA_08548 | XM_008603645.1 | 2.191 | 1.50E-02 | up | hypothetical protein BBA_08548 |
| BBA_08328 | XM_008603425.1 | 2.196 | 9.41E-07 | up | Pectin lyase fold/virulence factor |
| BBA_06196 | XM_008601293.1 | 2.207 | 3.10E-05 | up | monooxygenase, FAD-binding protein |
| BBA_03398 | XM_008598495.1 | 2.213 | 1.61E-06 | up | pyridoxal-dependent decarboxylase domain protein |
| BBA_06086 | XM_008601183.1 | 2.219 | 8.68E-04 | up | glycerophosphoryl diester phosphodiesterase family protein |
| BBA_09500 | XM_008604597.1 | 2.227 | 1.62E-03 | up | cuticle-degrading serine protease |
| MSTRG.14044 | MSTRG.14044.1 | 2.230 | 1.37E-03 | up | -- |
| BBA_06186 | XM_008601283.1 | 2.236 | 4.39E-06 | up | Catalase-like domain, heme-dependent |
| BBA_01032 | XM_008596129.1 | 2.238 | 2.49E-06 | up | nmrA-like family protein |
| BBA_08985 | XM_008604082.1 | 2.242 | 8.81E-04 | up | ThiJ/PfpI family protein |
| MSTRG.11466 | MSTRG.11466.1 | 2.243 | 4.63E-07 | up | glutathione-s-transferase |
| BBA_08804 | XM_008603901.1 | 2.246 | 7.41E-04 | up | hypothetical protein BBA_08804 |
| BBA_00718 | XM_008595815.1 | 2.247 | 1.92E-05 | up | mismatched base pair and cruciform dna recognition protein |
| BBA_09199 | XM_008604296.1 | 2.248 | 7.17E-03 | up | aminotransferase class-III |
| BBA_01615 | XM_008596712.1 | 2.251 | 2.07E-05 | up | aquaporin-2 |
| BBA_07844 | XM_008602941.1 | 2.255 | 2.47E-06 | up | O-methyltransferase, family 3 |
| MSTRG.13789 | MSTRG.13789.2 | 2.255 | 1.57E-06 | up | Pfs domain protein |
| BBA_05180 | XM_008600277.1 | 2.267 | 1.88E-06 | up | hypothetical protein BBA_05180 |
| BBA_06214 | XM_008601311.1 | 2.272 | 2.32E-06 | up | Cytochrome P450 CYP5293A1 |
| MSTRG.10547 | MSTRG.10547.1 | 2.289 | 1.95E-03 | up | hypothetical protein MAA_08203 |
| BBA_08729 | XM_008603826.1 | 2.290 | 4.09E-07 | up | phosphatidylserine decarboxylase family protein |
| BBA_08175 | XM_008603272.1 | 2.296 | 2.34E-02 | up | evolved D-lactonohydrolase |
| MSTRG.12969 | MSTRG.12969.1 | 2.296 | 2.40E-07 | up | hypothetical protein BBA_09296 |
| MSTRG.12 | MSTRG.12.1 | 2.298 | 2.59E-07 | up | ABC transporter transmembrane region |
| BBA_04690 | XM_008599787.1 | 2.301 | 2.39E-07 | up | Na,H/K antiporter P-type ATPase |
| BBA_03664 | XM_008598761.1 | 2.302 | 3.11E-04 | up | amine oxidase |
| MSTRG.7406 | MSTRG.7406.1 | 2.312 | 4.62E-03 | up | -- |
| BBA_09290 | XM_008604387.1 | 2.317 | 1.90E-07 | up | CFEM domain-containing protein |
| MSTRG.6258 | MSTRG.6258.1 | 2.322 | 8.31E-07 | up | metallopeptidase MepB |
| MSTRG.13028 | MSTRG.13028.1 | 2.326 | 2.14E-06 | up | catalase/peroxidase HPI |
| BBA_04679 | XM_008599776.1 | 2.329 | 5.42E-06 | up | urea amidolyase, putative |
| MSTRG.11100 | MSTRG.11100.1 | 2.330 | 9.12E-06 | up | hypothetical protein LEL_01859 |
| BBA_03683 | XM_008598780.1 | 2.330 | 1.39E-04 | up | 2OG-Fe(II) oxygenase family oxidoreductase |
| BBA_03935 | XM_008599032.1 | 2.332 | 8.47E-04 | up | hypothetical protein BBA_03935 |
| BBA_07371 | XM_008602468.1 | 2.339 | 3.82E-04 | up | alcohol dehydrogenase |
| BBA_04646 | XM_008599743.1 | 2.339 | 6.44E-07 | up | C6 zinc finger domain protein |
| BBA_05616 | XM_008600713.1 | 2.343 | 5.47E-07 | up | major facilitator superfamily transporter |
| BBA_02215 | XM_008597312.1 | 2.351 | 2.02E-07 | up | monocarboxylate transporter |
| BBA_06636 | XM_008601733.1 | 2.364 | 1.13E-07 | up | hypothetical protein BBA_06636 |
| BBA_03570 | XM_008598667.1 | 2.368 | 3.71E-03 | up | hypothetical protein BBA_03570 |
| BBA_02765 | XM_008597862.1 | 2.371 | 9.99E-08 | up | DJ-1/PfpI family protein |
| BBA_09103 | XM_008604200.1 | 2.375 | 2.14E-04 | up | glyoxalase-like protein |
| BBA_09257 | XM_008604354.1 | 2.375 | 7.83E-03 | up | cis,cis-muconate lactonizing enzyme precursor |
| BBA_07573 | XM_008602670.1 | 2.375 | 2.96E-03 | up | hypothetical protein BBA_07573 |
| MSTRG.12617 | MSTRG.12617.1 | 2.382 | 9.16E-08 | up | Ferric/cupric reductase transmembrane component 2 |
| MSTRG.9883 | MSTRG.9883.1 | 2.383 | 5.64E-04 | up | -- |
| BBA_10012 | XM_008605109.1 | 2.388 | 4.77E-04 | up | FluG domain-containing protein |
| BBA_02233 | XM_008597330.1 | 2.392 | 3.17E-07 | up | Glycoside hydrolase, catalytic core |
| MSTRG.5397 | MSTRG.5397.1 | 2.397 | 2.21E-05 | up | hypothetical protein BBO_01897 |
| MSTRG.7945 | MSTRG.7945.2 | 2.398 | 1.55E-03 | up | -- |
| BBA_04090 | XM_008599187.1 | 2.402 | 5.44E-06 | up | short-chain dehydrogenase |
| MSTRG.12972 | MSTRG.12972.1 | 2.403 | 9.09E-08 | up | hypothetical protein F503_05994 |
| BBA_03278 | XM_008598375.1 | 2.403 | 4.24E-07 | up | NAD dependent epimerase/dehydratase |
| BBA_06635 | XM_008601732.1 | 2.412 | 3.33E-07 | up | hypothetical protein BBA_06635 |
| MSTRG.6984 | MSTRG.6984.1 | 2.412 | 2.57E-02 | up | -- |
| BBA_00005 | XM_008595102.1 | 2.413 | 5.27E-08 | up | ABC transporter transmembrane region |
| BBA_09346 | XM_008604443.1 | 2.433 | 4.22E-07 | up | amidase-like protein |
| MSTRG.10303 | MSTRG.10303.1 | 2.434 | 6.07E-03 | up | hypothetical protein BBA_07376 |
| MSTRG.6591 | MSTRG.6591.1 | 2.435 | 5.58E-07 | up | -- |
| BBA_03965 | XM_008599062.1 | 2.438 | 1.27E-03 | up | aorsin-like protein |
| BBA_09428 | XM_008604525.1 | 2.461 | 4.51E-04 | up | peptidase family M3 |
| BBA_06212 | XM_008601309.1 | 2.464 | 3.34E-04 | up | MFS monocarboxylate transporter |
| BBA_08818 | XM_008603915.1 | 2.469 | 9.09E-08 | up | alkaline D-peptidase |
| BBA_09591 | XM_008604688.1 | 2.473 | 2.95E-03 | up | aspergillopepsin, putative |
| BBA_04657 | XM_008599754.1 | 2.478 | 8.10E-03 | up | hypothetical protein BBA_04657 |
| BBA_07323 | XM_008602420.1 | 2.492 | 4.23E-06 | up | 6-phosphogluconate dehydrogenase |
| BBA_03812 | XM_008598909.1 | 2.499 | 1.22E-03 | up | SCP-like extracellular protein |
| BBA_03412 | XM_008598509.1 | 2.502 | 1.61E-08 | up | putative cell wall glycoprotein |
| BBA_07377 | XM_008602474.1 | 2.502 | 3.84E-02 | up | hypothetical protein BBAD15_g8291 |
| BBA_06669 | XM_008601766.1 | 2.507 | 3.84E-02 | up | protein kinase |
| BBA_07147 | XM_008602244.1 | 2.510 | 1.82E-08 | up | hypothetical protein BBA_07147 |
| BBA_05173 | XM_008600270.1 | 2.513 | 2.73E-06 | up | Cytochrome P450 CYP52G6 |
| BBA_07175 | XM_008602272.1 | 2.513 | 2.75E-08 | up | FAD dependent oxidoreductase domain containing protein |
| MSTRG.13376 | MSTRG.13376.1 | 2.513 | 2.57E-04 | up | hypothetical protein BBA_07666 |
| BBA_07497 | XM_008602594.1 | 2.515 | 5.47E-07 | up | hypothetical protein BBA_07497 |
| BBA_08327 | XM_008603424.1 | 2.516 | 1.43E-08 | up | CFEM domain-containing protein |
| MSTRG.12973 | MSTRG.12973.1 | 2.525 | 1.32E-08 | up | hypothetical protein BBO_00726 |
| BBA_06480 | XM_008601577.1 | 2.539 | 8.35E-06 | up | oxidoreductase FAD-binding domain-containing protein |
| BBA_09923 | XM_008605020.1 | 2.555 | 4.62E-08 | up | putative peptidylarginine deiminase |
| BBA_10072 | XM_008605169.1 | 2.556 | 2.39E-06 | up | basic proline-rich protein |
| BBA_00091 | XM_008595188.1 | 2.557 | 1.93E-07 | up | 15-hydroxyprostaglandin dehydrogenase (NAD(+)) |
| BBA_09350 | XM_008604447.1 | 2.567 | 1.07E-08 | up | LysM domain-containing protein |
| BBA_08480 | XM_008603577.1 | 2.594 | 4.89E-09 | up | hypothetical protein BBA_08480 |
| MSTRG.2118 | MSTRG.2118.1 | 2.600 | 7.59E-08 | up | MOSC domain-containing protein |
| BBA_04278 | XM_008599375.1 | 2.606 | 1.48E-06 | up | pyridine nucleotide-disulfide oxidoreductase, putative |
| BBA_08219 | XM_008603316.1 | 2.623 | 3.19E-09 | up | polyketide synthase, putative |
| BBA_02631 | XM_008597728.1 | 2.626 | 3.11E-09 | up | hypothetical protein BBA_02631 |
| BBA_08221 | XM_008603318.1 | 2.629 | 2.84E-09 | up | 4-coumarate-CoA ligase |
| BBA_00193 | XM_008595290.1 | 2.636 | 3.48E-08 | up | galactose oxidase precursor |
| MSTRG.3723 | MSTRG.3723.1 | 2.640 | 2.48E-09 | up | hypothetical protein BBA_02631 |
| BBA_05174 | XM_008600271.1 | 2.641 | 3.97E-09 | up | hypothetical protein BBA_05174 |
| BBA_07528 | XM_008602625.1 | 2.651 | 7.37E-04 | up | hypothetical protein BBA_07528 |
| BBA_09919 | XM_008605016.1 | 2.651 | 9.57E-06 | up | hypothetical protein BBA_09919 |
| BBA_09289 | XM_008604386.1 | 2.656 | 2.04E-09 | up | Pectin lyase fold/virulence factor |
| BBA_10298 | XM_008605395.1 | 2.682 | 4.16E-05 | up | ABC transporter, transmembrane domain, type 1 |
| MSTRG.14139 | MSTRG.14139.2 | 2.684 | 4.11E-06 | up | Ribonuclease H-like protein |
| BBA_10357 | XM_008605454.1 | 2.695 | 2.51E-05 | up | hypothetical protein BBA_10357 |
| BBA_06166 | XM_008601263.1 | 2.712 | 5.68E-09 | up | alcohol dehydrogenase I |
| BBA_09927 | XM_008605024.1 | 2.716 | 2.15E-03 | up | pol-like protein |
| BBA_05177 | XM_008600274.1 | 2.718 | 1.53E-06 | up | enoyl reductase |
| BBA_09603 | XM_008604700.1 | 2.719 | 1.16E-04 | up | transposase-like protein |
| BBA_02167 | XM_008597264.1 | 2.720 | 1.50E-02 | up | hypothetical protein BBA_02167 |
| BBA_07370 | XM_008602467.1 | 2.721 | 4.77E-04 | up | penicillin-binding protein |
| BBA_09924 | XM_008605021.1 | 2.722 | 1.65E-09 | up | zinc finger protein |
| BBA_09271 | XM_008604368.1 | 2.724 | 8.47E-04 | up | hypothetical protein BBA_09271 |
| BBA_01537 | XM_008596634.1 | 2.726 | 1.03E-07 | up | hypothetical protein BBA_01537 |
| BBA_10322 | XM_008605419.1 | 2.741 | 5.36E-07 | up | hypothetical protein BBA_10322 |
| BBA_02036 | XM_008597133.1 | 2.764 | 3.71E-08 | up | iron permease FTR1 family protein |
| BBA_08967 | XM_008604064.1 | 2.768 | 1.50E-02 | up | fungal zinc cluster transcription factor |
| BBA_06717 | XM_008601814.1 | 2.775 | 1.80E-06 | up | putative YFW family protein 5 |
| MSTRG.13412 | MSTRG.13412.1 | 2.780 | 3.50E-03 | up | hypothetical protein BBAD15_g11840 |
| MSTRG.2192 | MSTRG.2192.1 | 2.791 | 4.19E-02 | up | hypothetical protein BBA_01536 |
| BBA_08686 | XM_008603783.1 | 2.812 | 2.17E-10 | up | UDP-glucosyltransferase |
| BBA_09429 | XM_008604526.1 | 2.813 | 4.19E-02 | up | hypothetical protein BBA_09429 |
| BBA_03633 | XM_008598730.1 | 2.816 | 2.41E-04 | up | hypothetical protein BBA_03633 |
| BBA_06838 | XM_008601935.1 | 2.823 | 4.64E-10 | up | phospholipase/carboxylesterase |
| BBA_00908 | XM_008596005.1 | 2.827 | 4.20E-04 | up | RTA1 like protein |
| MSTRG.8596 | MSTRG.8596.1 | 2.832 | 2.62E-03 | up | Chaperone protein DnaJ |
| MSTRG.7042 | MSTRG.7042.1 | 2.852 | 3.00E-02 | up | -- |
| BBA_09362 | XM_008604459.1 | 2.856 | 3.00E-02 | up | RTA-like protein |
| BBA_02750 | XM_008597847.1 | 2.858 | 3.00E-02 | up | DUF1772 family protein |
| BBA_01383 | XM_008596480.1 | 2.863 | 4.39E-06 | up | hypothetical protein BBA_01383 |
| BBA_08685 | XM_008603782.1 | 2.872 | 9.26E-11 | up | FkbM family methyltransferase |
| BBA_05175 | XM_008600272.1 | 2.874 | 6.67E-10 | up | LCCL domain-containing protein |
| BBA_09078 | XM_008604175.1 | 2.875 | 1.70E-05 | up | beta-hexosaminidase |
| BBA_05437 | XM_008600534.1 | 2.881 | 1.15E-10 | up | glycoside hydrolase family 2 |
| BBA_04328 | XM_008599425.1 | 2.892 | 2.33E-10 | up | aminotriazole resistance protein |
| MSTRG.1304 | MSTRG.1304.1 | 2.905 | 2.39E-07 | up | -- |
| BBA_06992 | XM_008602089.1 | 2.905 | 1.40E-07 | up | short chain dehydrogenase |
| BBA_08218 | XM_008603315.1 | 2.908 | 4.29E-08 | up | Acyl-CoA N-acyltransferase |
| BBA_06445 | XM_008601542.1 | 2.917 | 8.06E-10 | up | hypothetical protein BBA_06445 |
| BBA_08173 | XM_008603270.1 | 2.931 | 2.74E-10 | up | hypothetical protein BBA_08173 |
| BBA_03662 | XM_008598759.1 | 2.939 | 1.79E-07 | up | multidrug resistance protein MDR, putative |
| MSTRG.8561 | MSTRG.8561.1 | 2.943 | 1.54E-08 | up | f-box and wd-40 protein cdc4 |
| BBA_09297 | XM_008604394.1 | 2.950 | 1.37E-08 | up | hypothetical protein BBA_09297 |
| BBA_05678 | XM_008600775.1 | 2.953 | 2.80E-05 | up | nitrogen assimilation transcription factor nirA |
| BBA_08182 | XM_008603279.1 | 2.966 | 2.68E-11 | up | FAD binding domain-containing protein |
| MSTRG.3916 | MSTRG.3916.1 | 2.969 | 3.55E-04 | up | -- |
| BBA_04409 | XM_008599506.1 | 2.974 | 1.12E-03 | up | hypothetical protein BBA_04409 |
| BBA_04706 | XM_008599803.1 | 2.977 | 1.73E-08 | up | MFS multidrug resistance transporter |
| BBA_04772 | XM_008599869.1 | 2.988 | 4.60E-11 | up | mitochondrial protein |
| MSTRG.12672 | MSTRG.12672.1 | 3.002 | 1.19E-10 | up | hypothetical protein ASPVEDRAFT_653782 |
| BBA_04263 | XM_008599360.1 | 3.006 | 1.41E-11 | up | Bys1 family protein |
| BBA_02459 | XM_008597556.1 | 3.019 | 3.47E-11 | up | hypothetical protein BBA_02459 |
| MSTRG.12845 | MSTRG.12845.1 | 3.020 | 1.19E-06 | up | -- |
| BBA_07777 | XM_008602874.1 | 3.026 | 4.38E-10 | up | ABC metal ion transporter |
| BBA_08457 | XM_008603554.1 | 3.028 | 1.27E-10 | up | NACHT and WD40 domain protein |
| BBA_00081 | XM_008595178.1 | 3.047 | 1.50E-02 | up | hypothetical protein BBA_00081 |
| BBA_04689 | XM_008599786.1 | 3.048 | 1.50E-02 | up | hypothetical protein BBA_04689 |
| MSTRG.1341 | MSTRG.1341.1 | 3.056 | 1.09E-11 | up | hypothetical protein BBA_00932 |
| BBA_07148 | XM_008602245.1 | 3.060 | 5.65E-12 | up | hypothetical protein BBA_07148 |
| BBA_09925 | XM_008605022.1 | 3.062 | 1.36E-08 | up | tetratricopeptide repeat domain protein |
| BBA_03338 | XM_008598435.1 | 3.065 | 1.45E-09 | up | Bys1 family protein |
| BBA_08723 | XM_008603820.1 | 3.075 | 1.23E-11 | up | Epl1 protein |
| BBA_05184 | XM_008600281.1 | 3.099 | 2.07E-08 | up | Trypsin-related protease |
| BBA_01386 | XM_008596483.1 | 3.103 | 1.07E-02 | up | histone H2A, putative |
| BBA_03973 | XM_008599070.1 | 3.104 | 1.30E-04 | up | hypothetical protein BBA_03973 |
| MSTRG.10585 | MSTRG.10585.1 | 3.106 | 2.76E-12 | up | hypothetical protein BBA_07597 |
| BBA_03614 | XM_008598711.1 | 3.115 | 1.07E-02 | up | hypothetical protein BBA_03614 |
| MSTRG.10768 | MSTRG.10768.1 | 3.123 | 1.70E-03 | up | -- |
| BBA_03898 | XM_008598995.1 | 3.124 | 3.04E-12 | up | small secreted protein |
| MSTRG.4801 | MSTRG.4801.1 | 3.131 | 5.50E-09 | up | putative ABC transporter |
| BBA_03082 | XM_008598179.1 | 3.137 | 1.15E-05 | up | beta (1-3) glucanosyltransferase |
| MSTRG.93 | MSTRG.93.1 | 3.140 | 1.07E-02 | up | hypothetical protein BBA_00081 |
| BBA_07780 | XM_008602877.1 | 3.143 | 6.10E-10 | up | alpha-N-acetylglucosaminidase |
| BBA_08718 | XM_008603815.1 | 3.172 | 1.26E-03 | up | ankyrin repeat protein |
| BBA_10163 | XM_008605260.1 | 3.180 | 2.92E-12 | up | hypothetical protein BBA_10163 |
| BBA_02700 | XM_008597797.1 | 3.207 | 1.69E-07 | up | hypothetical protein BBA_02700 |
| BBA_09819 | XM_008604916.1 | 3.226 | 2.50E-10 | up | Tfo1 transposase |
| BBA_02996 | XM_008598093.1 | 3.246 | 2.11E-10 | up | antigenic cell wall galactomannoprotein, putative |
| BBA_09211 | XM_008604308.1 | 3.247 | 5.41E-03 | up | exo-beta-1,3-glucanase, putative |
| BBA_10068 | XM_008605165.1 | 3.249 | 1.79E-11 | up | hypothetical protein BBA_10068 |
| BBA_09212 | XM_008604309.1 | 3.258 | 2.99E-11 | up | hypothetical protein BBA_09212 |
| BBA_09108 | XM_008604205.1 | 3.264 | 3.73E-05 | up | hypothetical protein BBA_09108 |
| BBA_09293 | XM_008604390.1 | 3.275 | 1.30E-10 | up | subtilase-like protein |
| BBA_10233 | XM_008605330.1 | 3.294 | 6.08E-10 | up | sexual development transcription factor NsdD |
| BBA_02932 | XM_008598029.1 | 3.296 | 1.29E-04 | up | General substrate transporter |
| MSTRG.1153 | MSTRG.1153.1 | 3.307 | 4.34E-02 | up | UreD urease accessory protein |
| BBA_09423 | XM_008604520.1 | 3.324 | 7.56E-05 | up | translation factor (SUA5) |
| MSTRG.13035 | MSTRG.13035.2 | 3.362 | 2.97E-02 | up | -- |
| BBA_03053 | XM_008598150.1 | 3.383 | 1.44E-05 | up | abc transporter |
| BBA_07418 | XM_008602515.1 | 3.398 | 4.77E-11 | up | fungal specific transcription factor |
| BBA_10097 | XM_008605194.1 | 3.422 | 2.91E-07 | up | hypothetical protein BBA_10097 |
| BBA_08724 | XM_008603821.1 | 3.425 | 2.16E-04 | up | hypothetical protein BBA_08724 |
| BBA_04701 | XM_008599798.1 | 3.438 | 3.55E-11 | up | hypothetical protein BBA_04701 |
| BBA_05738 | XM_008600835.1 | 3.450 | 2.27E-12 | up | hypothetical protein BBA_05738 |
| MSTRG.12124 | MSTRG.12124.2 | 3.457 | 6.62E-14 | up | FMN-dependent dehydrogenase |
| BBA_09990 | XM_008605087.1 | 3.462 | 2.60E-05 | up | dipeptidyl aminopeptidase/acylaminoacyl peptidas |
| BBA_10147 | XM_008605244.1 | 3.474 | 2.60E-05 | up | hypothetical protein BBA_10147 |
| MSTRG.3658 | MSTRG.3658.1 | 3.511 | 5.89E-10 | up | hypothetical protein BBAD15_g4434 |
| MSTRG.5177 | MSTRG.5177.1 | 3.526 | 9.95E-04 | up | -- |
| BBA_09552 | XM_008604649.1 | 3.528 | 2.23E-07 | up | hypothetical protein BBA_09552 |
| BBA_07885 | XM_008602982.1 | 3.538 | 8.79E-08 | up | hypothetical protein BBA_07885 |
| MSTRG.11519 | MSTRG.11519.1 | 3.546 | 9.20E-05 | up | Acyl-CoA N-acyltransferase |
| BBA_09812 | XM_008604909.1 | 3.567 | 1.36E-02 | up | subtilase-like protein |
| BBA_09799 | XM_008604896.1 | 3.578 | 1.81E-06 | up | beta-1,3-glucanosyltransferase |
| BBA_08676 | XM_008603773.1 | 3.608 | 1.65E-09 | up | hypothetical protein BBA_08676 |
| BBA_02747 | XM_008597844.1 | 3.619 | 1.33E-10 | up | Cytochrome P450 CYP682N1 |
| BBA_01612 | XM_008596709.1 | 3.646 | 9.32E-03 | up | hypothetical protein BBA_01612 |
| BBA_10106 | XM_008605203.1 | 3.650 | 9.32E-03 | up | hypothetical protein BBA_10106 |
| BBA_08677 | XM_008603774.1 | 3.652 | 2.11E-15 | up | hypothetical protein BBA_08677 |
| BBA_10070 | XM_008605167.1 | 3.666 | 9.32E-03 | up | kynurenine 3-monooxygenase |
| MSTRG.12140 | MSTRG.12140.1 | 3.677 | 4.17E-16 | up | hypothetical protein BBA_08677 |
| BBA_00932 | XM_008596029.1 | 3.684 | 2.59E-10 | up | hypothetical protein BBA_00932 |
| BBA_03852 | XM_008598949.1 | 3.717 | 7.85E-16 | up | penicillin-binding protein |
| BBA_03667 | XM_008598764.1 | 3.737 | 4.18E-16 | up | methyltransferase domain-containing protein |
| BBA_08185 | XM_008603282.1 | 3.746 | 6.88E-17 | up | hypothetical protein BBA_08185 |
| BBA_01610 | XM_008596707.1 | 3.749 | 3.70E-13 | up | pyridoxamine 5&apos;-phosphate oxidase, putative |
| BBA_07278 | XM_008602375.1 | 3.755 | 1.71E-06 | up | hypothetical protein BBA_07278 |
| BBA_08662 | XM_008603759.1 | 3.759 | 5.84E-17 | up | Cytochrome P450 CYP561D2P |
| BBA_09936 | XM_008605033.1 | 3.817 | 4.32E-03 | up | hypothetical protein BBA_09936 |
| BBA_08179 | XM_008603276.1 | 3.827 | 1.70E-17 | up | polyketide synthase |
| BBA_06617 | XM_008601714.1 | 3.843 | 1.69E-09 | up | conidial pigment biosynthesis scytalone dehydratase Arp1 |
| BBA_09593 | XM_008604690.1 | 3.862 | 8.62E-17 | up | adenosine deaminase |
| BBA_04618 | XM_008599715.1 | 3.864 | 5.65E-16 | up | glucose-methanol-choline (gmc) oxidoreductase |
| BBA_03952 | XM_008599049.1 | 3.868 | 2.08E-08 | up | glutathione-dependent formaldehyde-activating, GFA |
| MSTRG.7480 | MSTRG.7480.1 | 3.868 | 3.23E-15 | up | Aminoglycoside phosphotransferase, partial |
| MSTRG.13424 | MSTRG.13424.1 | 3.876 | 1.07E-04 | up | phosphotransferase family protein |
| MSTRG.12675 | MSTRG.12675.1 | 3.917 | 3.34E-18 | up | hypothetical protein BBA_09080 |
| MSTRG.14361 | MSTRG.14361.1 | 3.980 | 1.97E-06 | up | -- |
| BBA_10365 | XM_008605460.1 | 4.004 | 1.70E-07 | up | restless-like transposase |
| BBA_09231 | XM_008604328.1 | 4.005 | 1.38E-03 | up | hypothetical protein BBA_09231 |
| MSTRG.13409 | MSTRG.13409.1 | 4.006 | 1.72E-11 | up | dsp1-1-like protein |
| MSTRG.14045 | MSTRG.14045.1 | 4.032 | 1.19E-06 | up | hypothetical protein BBA_07666 |
| BBA_09503 | XM_008604600.1 | 4.033 | 8.93E-08 | up | WD domain protein |
| MSTRG.13055 | MSTRG.13055.1 | 4.054 | 3.32E-19 | up | hypothetical protein BBA_09363 |
| MSTRG.12131 | MSTRG.12131.1 | 4.117 | 2.27E-18 | up | Cytochrome P450 CYP561D2P |
| BBA_09213 | XM_008604310.1 | 4.136 | 3.08E-08 | up | hypothetical protein BBA_09213 |
| BBA_02701 | XM_008597798.1 | 4.176 | 2.50E-09 | up | Asp-hemolysin |
| BBA_09109 | XM_008604206.1 | 4.177 | 3.78E-14 | up | Catalase-like domain, heme-dependent |
| BBA_04728 | XM_008599825.1 | 4.208 | 4.43E-19 | up | coenzyme A transferase |
| BBA_04832 | XM_008599929.1 | 4.209 | 4.85E-20 | up | Cytochrome P450 CYP684B2 |
| MSTRG.11470 | MSTRG.11470.1 | 4.230 | 3.06E-20 | up | hypothetical protein BBA_08185 |
| BBA_07336 | XM_008602433.1 | 4.248 | 3.82E-06 | up | Cytochrome P450 CYP623C1 |
| BBA_06804 | XM_008601901.1 | 4.305 | 2.30E-04 | up | MFS transporter, putative |
| BBA_09645 | XM_008604742.1 | 4.308 | 5.07E-20 | up | hypothetical protein BBA_09645 |
| BBA_08513 | XM_008603610.1 | 4.353 | 1.63E-04 | up | gluconate 5-dehydrogenase |
| BBA_04700 | XM_008599797.1 | 4.383 | 4.26E-18 | up | hypothetical protein BBA_04700 |
| BBA_04783 | XM_008599880.1 | 4.385 | 2.41E-10 | up | UDP-N-acetylmuramate--L-alanine ligase |
| BBA_02767 | XM_008597864.1 | 4.387 | 6.98E-12 | up | hypothetical protein BBA_02767 |
| BBA_05436 | XM_008600533.1 | 4.417 | 6.82E-22 | up | hypothetical protein BBA_05436 |
| BBA_07146 | XM_008602243.1 | 4.469 | 2.61E-22 | up | LysM domain-containing protein |
| BBA_09111 | XM_008604208.1 | 4.522 | 4.26E-21 | up | glucose-methanol-choline oxidoreductase |
| BBA_09107 | XM_008604204.1 | 4.542 | 3.98E-05 | up | hypothetical protein BBA_09107 |
| BBA_06496 | XM_008601593.1 | 4.612 | 1.12E-11 | up | penicillin-binding protein |
| BBA_03290 | XM_008598387.1 | 4.636 | 2.08E-09 | up | hypothetical protein BBA_03290 |
| BBA_09628 | XM_008604725.1 | 4.722 | 4.15E-08 | up | C6 zinc finger domain-containing protein |
| BBA_10254 | XM_008605351.1 | 4.763 | 2.55E-08 | up | hypothetical protein BBA_10254 |
| BBA_02614 | XM_008597711.1 | 4.784 | 2.03E-14 | up | hypothetical protein BBA_02614 |
| BBA_05679 | XM_008600776.1 | 4.785 | 3.71E-02 | up | HHE domain-containing protein |
| BBA_07530 | XM_008602627.1 | 4.790 | 3.71E-02 | up | trypsin-like protease |
| BBA_03993 | XM_008599090.1 | 4.795 | 3.71E-02 | up | HET domain protein |
| BBA_02552 | XM_008597649.1 | 4.799 | 3.71E-02 | up | serine/threonine protein kinase |
| BBA_09783 | XM_008604880.1 | 4.806 | 3.71E-02 | up | hypothetical protein BBA_09783 |
| BBA_07571 | XM_008602668.1 | 4.906 | 2.34E-02 | up | nuclear pore protein |
| BBA_09021 | XM_008604118.1 | 4.919 | 2.34E-02 | up | alcohol dehydrogenase, putative |
| BBA_09926 | XM_008605023.1 | 4.923 | 2.34E-02 | up | NF-X1 finger and helicase domain protein |
| BBA_02962 | XM_008598059.1 | 4.923 | 1.36E-13 | up | hypothetical protein BBA_02962 |
| MSTRG.12819 | MSTRG.12819.1 | 4.923 | 2.72E-25 | up | hypothetical protein BBA_09192 |
| BBA_04698 | XM_008599795.1 | 4.937 | 1.91E-21 | up | hypothetical protein BBA_04698 |
| BBA_09403 | XM_008604500.1 | 4.965 | 1.57E-06 | up | bZIP transcription factor |
| BBA_04498 | XM_008599595.1 | 5.031 | 1.49E-02 | up | putative sugar transporter |
| MSTRG.6885 | MSTRG.6885.1 | 5.031 | 1.49E-02 | up | Sec14 cytosolic factor |
| BBA_02344 | XM_008597441.1 | 5.033 | 1.49E-02 | up | succinate-semialdehyde dehydrogenase |
| BBA_02551 | XM_008597648.1 | 5.044 | 2.53E-26 | up | hypothetical protein BBA_02551 |
| BBA_07342 | XM_008602439.1 | 5.053 | 1.49E-02 | up | C6 transcription factor |
| BBA_09627 | XM_008604724.1 | 5.064 | 8.40E-10 | up | hypothetical protein BBA_09627 |
| BBA_09624 | XM_008604721.1 | 5.075 | 1.21E-14 | up | hypothetical protein BBA_09624 |
| MSTRG.13298 | MSTRG.13298.1 | 5.078 | 4.63E-07 | up | hypothetical protein BBA_07666 |
| BBA_10162 | XM_008605259.1 | 5.124 | 5.91E-16 | up | hypothetical protein BBA_10162 |
| MSTRG.10918 | MSTRG.10918.1 | 5.139 | 2.56E-07 | up | protease S8 tripeptidyl peptidase I (cln2) |
| BBA_04705 | XM_008599802.1 | 5.143 | 9.51E-03 | up | Cytochrome P450 CYP5293A1 |
| BBA_04967 | XM_008600064.1 | 5.145 | 9.51E-03 | up | crystal protein |
| BBA_06700 | XM_008601797.1 | 5.155 | 6.27E-14 | up | alkaline serine protease AorO |
| BBA_09586 | XM_008604683.1 | 5.165 | 2.70E-12 | up | LysM domain-containing protein |
| BBA_04777 | XM_008599874.1 | 5.169 | 9.51E-03 | up | short-chain dehydrogenase/reductase SDR |
| BBA_09401 | XM_008604498.1 | 5.172 | 9.51E-03 | up | subtilisin-like serine protease |
| MSTRG.84 | MSTRG.84.1 | 5.197 | 2.44E-17 | up | -- |
| BBA_06029 | XM_008601126.1 | 5.214 | 1.10E-07 | up | carboxymuconolactone decarboxylase |
| BBA_09822 | XM_008604919.1 | 5.235 | 9.88E-13 | up | Concanavalin A-like lectin/glucanase |
| BBA_01614 | XM_008596711.1 | 5.256 | 6.07E-03 | up | MFS multidrug resistance transporter |
| MSTRG.12184 | MSTRG.12184.1 | 5.258 | 7.80E-11 | up | -- |
| BBA_07421 | XM_008602518.1 | 5.261 | 6.07E-03 | up | Flavin-binding monooxygenase-like family protein |
| BBA_09597 | XM_008604694.1 | 5.265 | 6.07E-03 | up | oxidoreductase, short chain dehydrogenase/reductase |
| BBA_01712 | XM_008596809.1 | 5.270 | 6.07E-03 | up | hypothetical protein BBA_01712 |
| BBA_09935 | XM_008605032.1 | 5.277 | 6.07E-03 | up | hypothetical protein BBA_09935 |
| BBA_09625 | XM_008604722.1 | 5.323 | 3.55E-11 | up | dsp1-1-like protein |
| BBA_10075 | XM_008605172.1 | 5.484 | 7.62E-09 | up | hypothetical protein BBA_10075 |
| BBA_04647 | XM_008599744.1 | 5.505 | 1.45E-18 | up | H(+)-myo-inositol cotransporter |
| BBA_07775 | XM_008602872.1 | 5.567 | 3.56E-09 | up | hypothetical protein BBA_07775 |
| BBA_04722 | XM_008599819.1 | 5.604 | 2.21E-09 | up | hypothetical protein BBA_04722 |
| MSTRG.11463 | MSTRG.11463.1 | 5.608 | 1.06E-03 | up | laccase 2 |
| BBA_09937 | XM_008605034.1 | 5.617 | 1.06E-03 | up | hypothetical protein BBA_09937 |
| BBA_03712 | XM_008598809.1 | 5.617 | 1.06E-03 | up | hypothetical protein BBA_03712 |
| MSTRG.13398 | MSTRG.13398.2 | 5.631 | 1.06E-03 | up | hypothetical protein BBA_09620 |
| BBA_06442 | XM_008601539.1 | 5.645 | 7.04E-04 | up | hypothetical protein BBA_06442 |
| BBA_02613 | XM_008597710.1 | 5.664 | 1.37E-18 | up | multicopper oxidase |
| MSTRG.6644 | MSTRG.6644.2 | 5.701 | 6.77E-10 | up | hypothetical protein BBO_08998 |
| BBA_04707 | XM_008599804.1 | 5.746 | 4.71E-04 | up | hypothetical protein BBA_04707 |
| BBA_10253 | XM_008605350.1 | 5.775 | 1.09E-25 | up | sexual development transcription factor NsdD |
| BBA_08989 | XM_008604086.1 | 5.815 | 1.36E-16 | up | short-chain dehydrogenase |
| BBA_09585 | XM_008604682.1 | 5.845 | 3.11E-04 | up | symbiotic chitinase |
| BBA_05308 | XM_008600405.1 | 5.915 | 1.91E-21 | up | acyltransferase-like protein |
| MSTRG.12861 | MSTRG.12861.1 | 5.945 | 2.91E-27 | up | -- |
| BBA_09303 | XM_008604400.1 | 5.977 | 1.39E-04 | up | hypothetical protein BBA_09303 |
| BBA_08994 | XM_008604091.1 | 6.028 | 9.32E-05 | up | Antibiotic biosynthesis monooxygenase |
| BBA_07408 | XM_008602505.1 | 6.048 | 9.32E-05 | up | DJ-1/PfpI family protein |
| BBA_09192 | XM_008604289.1 | 6.136 | 1.86E-35 | up | hypothetical protein BBA_09192 |
| BBA_08993 | XM_008604090.1 | 6.244 | 1.93E-05 | up | conidial pigment biosynthesis scytalone dehydratase Arp1 |
| BBA_09619 | XM_008604716.1 | 6.294 | 1.34E-05 | up | hypothetical protein BBA_09619 |
| BBA_02766 | XM_008597863.1 | 6.417 | 4.33E-06 | up | BTB domain transcription factor |
| BBA_04723 | XM_008599820.1 | 6.425 | 4.33E-06 | up | hypothetical protein BBA_04723 |
| MSTRG.13668 | MSTRG.13668.1 | 6.432 | 4.33E-06 | up | -- |
| BBA_10255 | XM_008605352.1 | 6.434 | 3.73E-30 | up | Methyltransferase type 11 |
| BBA_07412 | XM_008602509.1 | 6.522 | 3.96E-25 | up | hypothetical protein BBA_07412 |
| BBA_04708 | XM_008599805.1 | 6.601 | 1.08E-06 | up | hypothetical protein BBA_04708 |
| BBA_00455 | XM_008595552.1 | 6.678 | 1.43E-15 | up | MYND domain protein, putative |
| BBA_07337 | XM_008602434.1 | 6.917 | 5.70E-08 | up | Alcohol dehydrogenase |
| BBA_03972 | XM_008599069.1 | 7.116 | 9.47E-09 | up | hypothetical protein BBA_03972 |
| MSTRG.1984 | MSTRG.1984.1 | 7.126 | 7.08E-09 | up | hypothetical protein BBA_01384 |
| MSTRG.13363 | MSTRG.13363.1 | 7.130 | 3.69E-26 | up | major facilitator superfamily transporter |
| BBA_06031 | XM_008601128.1 | 7.193 | 3.97E-09 | up | metallo-beta-lactamase superfamily protein |
| BBA_02137 | XM_008597234.1 | 7.206 | 4.23E-19 | up | putative peptidylarginine deiminase |
| MSTRG.12865 | MSTRG.12865.1 | 7.239 | 2.29E-09 | up | hypothetical protein BBA_09231 |
| BBA_09590 | XM_008604687.1 | 7.333 | 7.86E-10 | up | major facilitator superfamily transporter |
| BBA_04648 | XM_008599745.1 | 7.470 | 1.76E-10 | up | Cytochrome P450 CYP5280A1P |
| MSTRG.6646 | MSTRG.6646.2 | 7.644 | 2.62E-11 | up | -- |
| BBA_09814 | XM_008604911.1 | 7.690 | 1.29E-11 | up | hypothetical protein BBA_09814 |
| BBA_09589 | XM_008604686.1 | 7.970 | 3.70E-13 | up | autophagy-related protein 22 |
| BBA_00085 | XM_008595182.1 | 8.284 | 1.76E-45 | up | Orf2-like protein |
| BBA_04656 | XM_008599753.1 | 9.134 | 9.80E-21 | up | hypothetical protein BBA_04656 |
| BBA_09626 | XM_008604723.1 | 9.656 | 1.43E-24 | up | hypothetical protein BBA_09626 |
| BBA_09820 | XM_008604917.1 | 9.861 | 1.76E-45 | up | hypothetical protein BBA_09820 |
| MSTRG.13396 | MSTRG.13396.1 | 9.885 | 3.01E-26 | up | hypothetical protein BBAD15_g4434 |
| BBA_07413 | XM_008602510.1 | 10.324 | 1.03E-29 | up | short chain dehydrogenase |
| BBA_05311 | XM_008600408.1 | 10.613 | 6.13E-32 | up | UDP-N-acetylglucosamine acyltransferase |
| MSTRG.13990 | MSTRG.13990.1 | 11.377 | 2.68E-38 | up | hypothetical protein VFPPC_18339 |
| BBA_05312 | XM_008600409.1 | 11.692 | 1.89E-61 | up | pleiotropic regulatory protein DegT/DnrJ/EryC1/StrS |

**Table S5.** GO classification of differentially expressed genes in the transcriptome of Δ*dim5* versus WT.

| **GO ID** | **GO_term** | **GO_function** | **Counts of genes** | | | | ***p* value** | **Level** |
| --- | --- | --- | --- | --- | --- | --- | --- | --- |
|  |  |  | **Hit** | **up** | **down** | **background** |  |  |
| GO:0044710 | single-organism metabolic process | Biological Process | 194 | 109 | 85 | 1616 | 3.97E-05 | NA |
| GO:0055114 | oxidation-reduction process | Biological Process | 148 | 81 | 67 | 847 | 2.70E-15 | L02 |
| GO:0055085 | transmembrane transport | Biological Process | 101 | 51 | 50 | 696 | 2.67E-06 | L02 |
| GO:0006811 | ion transport | Biological Process | 35 | 13 | 22 | 266 | 2.32E-02 | L04 |
| GO:0044282 | small molecule catabolic process | Biological Process | 11 | 7 | 4 | 65 | 3.70E-02 | L03 |
| GO:0016054 | organic acid catabolic process | Biological Process | 10 | 6 | 4 | 54 | 2.61E-02 | L04 |
| GO:0046395 | carboxylic acid catabolic process | Biological Process | 10 | 6 | 4 | 54 | 2.61E-02 | L05 |
| GO:0009063 | cellular amino acid catabolic process | Biological Process | 9 | 5 | 4 | 41 | 1.18E-02 | L04 |
| GO:1901606 | alpha-amino acid catabolic process | Biological Process | 8 | 4 | 4 | 31 | 6.32E-03 | L05 |
| GO:0006026 | aminoglycan catabolic process | Biological Process | 7 | 5 | 2 | 17 | 5.09E-04 | L05 |
| GO:0072593 | reactive oxygen species metabolic process | Biological Process | 7 | 4 | 3 | 22 | 2.95E-03 | L03 |
| GO:1901136 | carbohydrate derivative catabolic process | Biological Process | 7 | 5 | 2 | 28 | 1.25E-02 | L04 |
| GO:0006022 | aminoglycan metabolic process | Biological Process | 7 | 5 | 2 | 30 | 1.82E-02 | L04 |
| GO:0006032 | chitin catabolic process | Biological Process | 6 | 5 | 1 | 15 | 1.56E-03 | L06 |
| GO:1901072 | glucosamine-containing compound catabolic process | Biological Process | 6 | 5 | 1 | 15 | 1.56E-03 | L06 |
| GO:0046348 | amino sugar catabolic process | Biological Process | 6 | 5 | 1 | 17 | 3.28E-03 | L05 |
| GO:0006030 | chitin metabolic process | Biological Process | 6 | 5 | 1 | 25 | 2.47E-02 | L05 |
| GO:1901071 | glucosamine-containing compound metabolic process | Biological Process | 6 | 5 | 1 | 28 | 4.13E-02 | L05 |
| GO:0019748 | secondary metabolic process | Biological Process | 5 | 5 | 0 | 12 | 3.19E-03 | L02 |
| GO:0097164 | ammonium ion metabolic process | Biological Process | 5 | 2 | 3 | 20 | 3.33E-02 | L03 |
| GO:0018958 | phenol-containing compound metabolic process | Biological Process | 4 | 3 | 1 | 8 | 3.90E-03 | L04 |
| GO:0044550 | secondary metabolite biosynthetic process | Biological Process | 4 | 4 | 0 | 8 | 3.90E-03 | L03 |
| GO:0042744 | hydrogen peroxide catabolic process | Biological Process | 4 | 3 | 1 | 9 | 6.50E-03 | L04 |
| GO:0042743 | hydrogen peroxide metabolic process | Biological Process | 4 | 3 | 1 | 10 | 1.00E-02 | L04 |
| GO:0009074 | aromatic amino acid family catabolic process | Biological Process | 4 | 3 | 1 | 15 | 4.48E-02 | L05 |
| GO:0006582 | melanin metabolic process | Biological Process | 3 | 3 | 0 | 3 | 8.13E-04 | L03 |
| GO:0042438 | melanin biosynthetic process | Biological Process | 3 | 3 | 0 | 3 | 8.13E-04 | L04 |
| GO:0046189 | phenol-containing compound biosynthetic process | Biological Process | 3 | 3 | 0 | 3 | 8.13E-04 | L05 |
| GO:0015846 | polyamine transport | Biological Process | 3 | 1 | 2 | 4 | 3.03E-03 | L05 |
| GO:0009065 | glutamine family amino acid catabolic process | Biological Process | 3 | 1 | 2 | 6 | 1.31E-02 | L06 |
| GO:0015848 | spermidine transport | Biological Process | 2 | 0 | 2 | 2 | 8.72E-03 | L06 |
| GO:0015695 | organic cation transport | Biological Process | 2 | 0 | 2 | 3 | 2.45E-02 | L05 |
| GO:0006422 | aspartyl-tRNA aminoacylation | Biological Process | 2 | 1 | 1 | 4 | 4.61E-02 | L07 |
| GO:0006768 | biotin metabolic process | Biological Process | 2 | 0 | 2 | 4 | 4.61E-02 | L04 |
| GO:0009102 | biotin biosynthetic process | Biological Process | 2 | 0 | 2 | 4 | 4.61E-02 | L05 |
| GO:0015696 | ammonium transport | Biological Process | 2 | 0 | 2 | 4 | 4.61E-02 | L05 |
| GO:0016020 | membrane | Cellular Component | 313 | 166 | 147 | 2742 | 4.01E-06 | L02 |
| GO:0044425 | membrane part | Cellular Component | 307 | 161 | 146 | 2620 | 3.99E-07 | NA |
| GO:0031224 | intrinsic component of membrane | Cellular Component | 304 | 158 | 146 | 2474 | 2.48E-09 | L02 |
| GO:0016021 | integral component of membrane | Cellular Component | 303 | 157 | 146 | 2467 | 2.85E-09 | L03 |
| GO:0005618 | cell wall | Cellular Component | 7 | 5 | 2 | 27 | 1.02E-02 | L03 |
| GO:0030312 | external encapsulating structure | Cellular Component | 7 | 5 | 2 | 27 | 1.02E-02 | L02 |
| GO:0003824 | catalytic activity | Molecular Function | 468 | 256 | 212 | 4248 | 3.78E-08 | L01 |
| GO:0016491 | oxidoreductase activity | Molecular Function | 165 | 93 | 72 | 933 | 1.21E-17 | L02 |
| GO:0043169 | cation binding | Molecular Function | 151 | 87 | 64 | 1288 | 1.09E-03 | L03 |
| GO:0046872 | metal ion binding | Molecular Function | 150 | 86 | 64 | 1283 | 1.27E-03 | L04 |
| GO:0046914 | transition metal ion binding | Molecular Function | 103 | 62 | 41 | 844 | 2.12E-03 | L05 |
| GO:0005215 | transporter activity | Molecular Function | 68 | 37 | 31 | 501 | 8.95E-04 | L01 |
| GO:0022857 | transmembrane transporter activity | Molecular Function | 59 | 31 | 28 | 405 | 3.28E-04 | L02 |
| GO:0004497 | monooxygenase activity | Molecular Function | 48 | 26 | 22 | 161 | 9.61E-14 | L03 |
| GO:0048037 | cofactor binding | Molecular Function | 46 | 24 | 22 | 354 | 1.28E-02 | NA |
| GO:0020037 | heme binding | Molecular Function | 42 | 22 | 20 | 122 | 1.40E-14 | L04 |
| GO:0046906 | tetrapyrrole binding | Molecular Function | 42 | 22 | 20 | 122 | 1.40E-14 | L03 |
| GO:0016705 | oxidoreductase activity, acting on paired donors, with incorporation or reduction of molecular oxygen | Molecular Function | 41 | 21 | 20 | 142 | 2.00E-11 | L03 |
| GO:0016788 | hydrolase activity, acting on ester bonds | Molecular Function | 40 | 16 | 24 | 313 | 2.48E-02 | L03 |
| GO:0022891 | substrate-specific transmembrane transporter activity | Molecular Function | 39 | 18 | 21 | 304 | 2.50E-02 | NA |
| GO:0005506 | iron ion binding | Molecular Function | 38 | 18 | 20 | 137 | 4.03E-10 | L06 |
| GO:0015075 | ion transmembrane transporter activity | Molecular Function | 30 | 11 | 19 | 230 | 3.74E-02 | L03 |
| GO:0004175 | endopeptidase activity | Molecular Function | 28 | 13 | 15 | 173 | 2.69E-03 | L04 |
| GO:0004553 | hydrolase activity, hydrolyzing O-glycosyl compounds | Molecular Function | 27 | 20 | 7 | 153 | 8.62E-04 | L04 |
| GO:0016798 | hydrolase activity, acting on glycosyl bonds | Molecular Function | 27 | 20 | 7 | 169 | 3.82E-03 | L03 |
| GO:0022804 | active transmembrane transporter activity | Molecular Function | 25 | 16 | 9 | 164 | 9.56E-03 | L03 |
| GO:0008236 | serine-type peptidase activity | Molecular Function | 23 | 9 | 14 | 122 | 8.05E-04 | L04 |
| GO:0017171 | serine hydrolase activity | Molecular Function | 23 | 9 | 14 | 122 | 8.05E-04 | L03 |
| GO:0050660 | flavin adenine dinucleotide binding | Molecular Function | 23 | 13 | 10 | 149 | 1.09E-02 | L04 |
| GO:0019842 | vitamin binding | Molecular Function | 21 | 9 | 12 | 123 | 4.65E-03 | L03 |
| GO:0042626 | ATPase activity, coupled to transmembrane movement of substances | Molecular Function | 20 | 14 | 6 | 120 | 7.44E-03 | L05 |
| GO:0016820 | hydrolase activity, acting on acid anhydrides, catalyzing transmembrane movement of substances | Molecular Function | 20 | 14 | 6 | 122 | 8.92E-03 | NA |
| GO:0015399 | primary active transmembrane transporter activity | Molecular Function | 20 | 14 | 6 | 124 | 1.06E-02 | L04 |
| GO:0015405 | P-P-bond-hydrolysis-driven transmembrane transporter activity | Molecular Function | 20 | 14 | 6 | 124 | 1.06E-02 | NA |
| GO:0043492 | ATPase activity, coupled to movement of substances | Molecular Function | 20 | 14 | 6 | 125 | 1.16E-02 | L08 |
| GO:0004252 | serine-type endopeptidase activity | Molecular Function | 19 | 9 | 10 | 88 | 3.97E-04 | L05 |
| GO:0051213 | dioxygenase activity | Molecular Function | 14 | 4 | 10 | 61 | 1.20E-03 | L03 |
| GO:0004519 | endonuclease activity | Molecular Function | 14 | 6 | 8 | 88 | 3.28E-02 | L05 |
| GO:0016209 | antioxidant activity | Molecular Function | 11 | 5 | 6 | 38 | 5.02E-04 | L01 |
| GO:0031177 | phosphopantetheine binding | Molecular Function | 10 | 5 | 5 | 40 | 3.03E-03 | L03 |
| GO:0072341 | modified amino acid binding | Molecular Function | 10 | 5 | 5 | 43 | 5.30E-03 | L02 |
| GO:0033218 | amide binding | Molecular Function | 10 | 5 | 5 | 56 | 3.28E-02 | L02 |
| GO:0071949 | FAD binding | Molecular Function | 9 | 6 | 3 | 45 | 2.14E-02 | L05 |
| GO:0004601 | peroxidase activity | Molecular Function | 8 | 4 | 4 | 25 | 1.43E-03 | L02 |
| GO:0016684 | oxidoreductase activity, acting on peroxide as acceptor | Molecular Function | 8 | 4 | 4 | 26 | 1.90E-03 | L03 |
| GO:0010181 | FMN binding | Molecular Function | 8 | 5 | 3 | 36 | 1.60E-02 | L04 |
| GO:0008061 | chitin binding | Molecular Function | 7 | 7 | 0 | 17 | 5.09E-04 | L03 |
| GO:0004523 | RNA-DNA hybrid ribonuclease activity | Molecular Function | 7 | 6 | 1 | 29 | 1.52E-02 | L06 |
| GO:0016709 | oxidoreductase activity, acting on paired donors, with incorporation or reduction of molecular oxygen, NAD(P)H as one donor, and incorporation of one atom of oxygen | Molecular Function | 7 | 4 | 3 | 34 | 3.49E-02 | L04 |
| GO:0004568 | chitinase activity | Molecular Function | 6 | 5 | 1 | 15 | 1.56E-03 | L05 |
| GO:0008484 | sulfuric ester hydrolase activity | Molecular Function | 5 | 2 | 3 | 9 | 6.44E-04 | L04 |
| GO:0004096 | catalase activity | Molecular Function | 4 | 3 | 1 | 8 | 3.90E-03 | L03 |
| GO:0003993 | acid phosphatase activity | Molecular Function | 4 | 0 | 4 | 14 | 3.54E-02 | L06 |
| GO:0030411 | scytalone dehydratase activity | Molecular Function | 3 | 3 | 0 | 3 | 8.13E-04 | L05 |
| GO:0015203 | polyamine transmembrane transporter activity | Molecular Function | 3 | 1 | 2 | 4 | 3.03E-03 | L03 |
| GO:0004563 | beta-N-acetylhexosaminidase activity | Molecular Function | 3 | 2 | 1 | 5 | 7.04E-03 | L06 |
| GO:0005199 | structural constituent of cell wall | Molecular Function | 3 | 2 | 1 | 5 | 7.04E-03 | L02 |
| GO:0015929 | hexosaminidase activity | Molecular Function | 3 | 2 | 1 | 6 | 1.31E-02 | L05 |
| GO:0004784 | superoxide dismutase activity | Molecular Function | 3 | 1 | 2 | 7 | 2.14E-02 | L02 |
| GO:0016721 | oxidoreductase activity, acting on superoxide radicals as acceptor | Molecular Function | 3 | 1 | 2 | 7 | 2.14E-02 | L03 |
| GO:0018580 | nitronate monooxygenase activity | Molecular Function | 3 | 2 | 1 | 9 | 4.45E-02 | L05 |
| GO:0015606 | spermidine transmembrane transporter activity | Molecular Function | 2 | 0 | 2 | 2 | 8.72E-03 | L04 |
| GO:0052856 | NADHX epimerase activity | Molecular Function | 2 | 2 | 0 | 2 | 8.72E-03 | L04 |
| GO:0005275 | amine transmembrane transporter activity | Molecular Function | 2 | 2 | 0 | 3 | 2.45E-02 | L03 |
| GO:0008889 | glycerophosphodiester phosphodiesterase activity | Molecular Function | 2 | 1 | 1 | 3 | 2.45E-02 | L06 |
| GO:0015101 | organic cation transmembrane transporter activity | Molecular Function | 2 | 0 | 2 | 3 | 2.45E-02 | L05 |
| GO:0003847 | 1-alkyl-2-acetylglycerophosphocholine esterase activity | Molecular Function | 2 | 2 | 0 | 4 | 4.61E-02 | L05 |
| GO:0004411 | homogentisate 1,2-dioxygenase activity | Molecular Function | 2 | 1 | 1 | 4 | 4.61E-02 | L05 |
| GO:0004657 | proline dehydrogenase activity | Molecular Function | 2 | 1 | 1 | 4 | 4.61E-02 | L04 |
| GO:0004815 | aspartate-tRNA ligase activity | Molecular Function | 2 | 1 | 1 | 4 | 4.61E-02 | L05 |
| GO:0008519 | ammonium transmembrane transporter activity | Molecular Function | 2 | 0 | 2 | 4 | 4.61E-02 | L06 |

**Table S6.** Enriched KEGG pathways of differentially expressed genes in the transcriptome of Δ*dim5* versus WT.

| **Pathway ID** | **KEGG pathway** | **Counts of genes** | | | | ***p* value** |
| --- | --- | --- | --- | --- | --- | --- |
|  |  | **Hit** | **up** | **down** | **background** |  |
| ko00380 | Tryptophan metabolism | 13 | 6 | 7 | 57 | 1.51E-05 |
| ko00564 | Glycerophospholipid metabolism | 11 | 2 | 9 | 58 | 3.98E-04 |
| ko00565 | Ether lipid metabolism | 6 | 1 | 5 | 22 | 1.22E-03 |
| ko00511 | Other glycan degradation | 5 | 4 | 1 | 14 | 8.20E-04 |
| ko00531 | Glycosaminoglycan degradation | 4 | 3 | 1 | 10 | 1.76E-03 |
| ko00780 | Biotin metabolism | 4 | 1 | 3 | 10 | 1.76E-03 |
| ko00604 | Glycosphingolipid biosynthesis - ganglio series | 3 | 2 | 1 | 6 | 3.39E-03 |
| ko01200 | Carbon metabolism | 12 | 8 | 4 | 120 | 4.40E-02 |
| ko00520 | Amino sugar and nucleotide sugar metabolism | 9 | 5 | 4 | 67 | 1.43E-02 |
| ko04146 | Peroxisome | 8 | 4 | 4 | 61 | 2.33E-02 |
| ko00350 | Tyrosine metabolism | 7 | 5 | 2 | 47 | 1.74E-02 |
| ko00071 | Fatty acid degradation | 5 | 2 | 3 | 32 | 3.52E-02 |
| ko00910 | Nitrogen metabolism | 4 | 1 | 3 | 16 | 1.16E-02 |
| ko04016 | MAPK signaling pathway - plant | 3 | 2 | 1 | 8 | 8.69E-03 |
| ko00603 | Glycosphingolipid biosynthesis - globo and isoglobo series | 3 | 2 | 1 | 10 | 1.71E-02 |

**Table S7**. Differentially expressed genes associated with phenotypic changes and genome stability of Δ*dim5* mutant

| **Gene_ID** | **Transcript_ID** | **log_2_ *R*** | **FDR** | **regulation** | **NR_Annotation** |
| --- | --- | --- | --- | --- | --- |
| **Involved in cuticle degradation and insect pathogenicity** | | | |  |  |
| BBA_00049 | XM_008595146.1 | -2.789 | 4.12E-10 | down | intracellular serine protease |
| BBA_00428 | XM_008595525.1 | -2.542 | 1.22E-07 | down | fatty acid hydroxylase superfamily protein |
| BBA_06599 | XM_008601696.1 | -2.462 | 2.79E-08 | down | adhesin protein Mad2 |
| MSTRG.10594 | MSTRG.10594.1 | -2.379 | 1.37E-07 | down | hemolysin-III family protein |
| MSTRG.8214 | MSTRG.8214.1 | -2.256 | 2.07E-05 | down | Chitinase II |
| BBA_02800 | XM_008597897.1 | -2.177 | 2.92E-06 | down | filamentous hemagglutinin / adhesin |
| BBA_09498 | XM_008604595.1 | -1.997 | 1.05E-05 | down | lipase/thioesterase family protein |
| BBA_07700 | XM_008602797.1 | -1.851 | 9.54E-05 | down | lipase class 2 |
| BBA_04181 | XM_008599278.1 | -1.680 | 3.21E-04 | down | fatty acid hydroxylase superfamily protein |
| BBA_10302 | XM_008605399.1 | -1.660 | 1.37E-03 | down | subtilase-like protein |
| BBA_00200 | XM_008595297.1 | -1.607 | 2.55E-03 | down | glycoside hydrolase family 55 |
| BBA_09825 | XM_008604922.1 | -1.604 | 6.70E-04 | down | cutinase-like protein |
| BBA_00196 | XM_008595293.1 | -1.467 | 7.91E-03 | down | fatty acid hydroxylase superfamily protein |
| BBA_07683 | XM_008602780.1 | -1.295 | 4.74E-02 | down | zeta toxin |
| BBA_08911 | XM_008604008.1 | -1.267 | 2.38E-02 | down | zeta toxin |
| BBA_04201 | XM_008599298.1 | -1.072 | 4.42E-02 | down | family S53 protease |
| BBA_03054 | XM_008598151.1 | -1.060 | 4.88E-02 | down | metallopeptidase family M24 |
| BBA_02720 | XM_008597817.1 | 1.064 | 4.90E-02 | up | glycoside hydrolase family 16 protein |
| MSTRG.3120 | MSTRG.3120.1 | 1.235 | 1.49E-02 | up | chitinase-like protein |
| BBA_04500 | XM_008599597.1 | 1.398 | 6.01E-03 | up | Glycoside hydrolase, family 31 |
| BBA_08505 | XM_008603602.1 | 1.408 | 1.12E-02 | up | subtilase-like protein |
| MSTRG.11685 | MSTRG.11685.1 | 1.616 | 6.70E-04 | up | Pectin lyase fold/virulence factor |
| BBA_02214 | XM_008597311.1 | 1.658 | 6.95E-04 | up | subtilase-like protein |
| MSTRG.11166 | MSTRG.11166.1 | 1.660 | 3.84E-03 | up | family S53 protease-like protein |
| BBA_02230 | XM_008597327.1 | 1.693 | 2.59E-04 | up | chitinase-like protein |
| BBA_02941 | XM_008598038.1 | 2.158 | 4.09E-06 | up | chitinase A1 |
| BBA_00297 | XM_008595394.1 | 2.183 | 1.25E-06 | up | class V chitinase, putative |
| BBA_08328 | XM_008603425.1 | 2.196 | 9.41E-07 | up | Pectin lyase fold/virulence factor |
| BBA_09500 | XM_008604597.1 | 2.227 | 1.62E-03 | up | cuticle-degrading serine protease |
| MSTRG.6258 | MSTRG.6258.1 | 2.322 | 8.31E-07 | up | metallopeptidase MepB |
| BBA_02233 | XM_008597330.1 | 2.392 | 3.17E-07 | up | Glycoside hydrolase, catalytic core |
| BBA_09289 | XM_008604386.1 | 2.656 | 2.04E-09 | up | Pectin lyase fold/virulence factor |
| BBA_05437 | XM_008600534.1 | 2.881 | 1.15E-10 | up | glycoside hydrolase family 2 |
| BBA_05184 | XM_008600281.1 | 3.099 | 2.07E-08 | up | Trypsin-related protease |
| BBA_09293 | XM_008604390.1 | 3.275 | 1.30E-10 | up | subtilase-like protein |
| BBA_09812 | XM_008604909.1 | 3.567 | 1.36E-02 | up | subtilase-like protein |
| BBA_07530 | XM_008602627.1 | 4.790 | 3.71E-02 | up | trypsin-like protease |
| BBA_06700 | XM_008601797.1 | 5.155 | 6.27E-14 | up | alkaline serine protease AorO |
| BBA_09401 | XM_008604498.1 | 5.172 | 9.51E-03 | up | subtilisin-like serine protease |
|  |  |  |  |  |  |
| **Involved in asexual development** | |  |  |  |  |
| BBA_04401 | XM_008599498.1 | -1.826 | 8.50E-05 | down | sporulation-specific protein |
| BBA_02876 | XM_008597973.1 | -1.392 | 6.76E-03 | down | vivid PAS protein VVD |
| BBA_01528 | XM_008596625.1 | -1.082 | 4.74E-02 | down | frequency clock protein |
|  |  |  |  |  |  |
| **Involved in antioxidant activity** | |  |  |  |  |
| MSTRG.7321 | MSTRG.7321.1 | -1.694 | 1.91E-02 | down | thiol-specific monooxygenase |
| BBA_04084 | XM_008599181.1 | -1.419 | 3.40E-03 | down | cytosol Mn-superoxide dismutase |
| BBA_02077 | XM_008597174.1 | -1.315 | 8.12E-03 | down | Catalase-like domain, heme-dependent |
| BBA_07807 | XM_008602904.1 | -1.179 | 2.15E-02 | down | cytosol Mn-superoxide dismutase |
| BBA_03594 | XM_008598691.1 | 1.296 | 1.98E-02 | up | Thioredoxin-like protein |
| BBA_02311 | XM_008597408.1 | 1.547 | 1.08E-03 | up | Cu/Zn superoxide dismutase |
| BBA_06186 | XM_008601283.1 | 2.236 | 4.39E-06 | up | Catalase-like domain, heme-dependent |
| MSTRG.13028 | MSTRG.13028.1 | 2.326 | 2.14E-06 | up | catalase/peroxidase HPI |
| BBA_09109 | XM_008604206.1 | 4.177 | 3.78E-14 | up | Catalase-like domain, heme-dependent |
|  |  |  |  |  |  |
| **Involved in cell wall composition and response to cell wall perturbation** | | | | |  |
| BBA_07286 | XM_008602383.1 | -2.926 | 4.18E-11 | down | hydrophobin-like protein |
| MSTRG.5285 | MSTRG.5285.1 | -1.058 | 4.88E-02 | down | GPI anchored protein, putative |
| BBA_08214 | XM_008603311.1 | 1.426 | 6.37E-03 | up | cell wall glucanosyltransferase Mwg1 |
| BBA_01780 | XM_008596877.1 | 1.633 | 1.14E-03 | up | putative xylanase 1 |
| BBA_05929 | XM_008601026.1 | 1.659 | 3.59E-04 | up | WSC domain-containing protein |
| BBA_00525 | XM_008595622.1 | 2.132 | 2.34E-06 | up | GPI anchored cell wall protein |
| BBA_02602 | XM_008597699.1 | 2.173 | 1.19E-06 | up | cell wall protein |
| BBA_03412 | XM_008598509.1 | 2.502 | 1.61E-08 | up | putative cell wall glycoprotein |
| BBA_02996 | XM_008598093.1 | 3.246 | 2.11E-10 | up | antigenic cell wall galactomannoprotein, putative |
| BBA_09822 | XM_008604919.1 | 5.235 | 9.88E-13 | up | Concanavalin A-like lectin/glucanase |
|  |  |  |  |  |  |
| **Involved in cell tolerance to heat and UV irradiation** | | | |  |  |
| MSTRG.5855 | MSTRG.5855.1 | -1.873 | 1.15E-03 | down | UV-endonuclease UvdE |
| BBA_10341 | XM_008605438.1 | -1.330 | 8.40E-03 | down | DnaJ domain-containing protein |
| BBA_03638 | XM_008598735.1 | -1.269 | 1.35E-02 | down | DNA photolyase |
| BBA_06849 | XM_008601946.1 | 1.133 | 3.30E-02 | up | thermotolerance protein |
| BBA_08688 | XM_008603785.1 | 1.680 | 5.34E-04 | up | heat shock protein 30 |
| BBA_07886 | XM_008602983.1 | 1.818 | 1.02E-04 | up | hsp20-like protein |
| MSTRG.8596 | MSTRG.8596.1 | 2.832 | 2.62E-03 | up | Chaperone protein DnaJ |
|  |  |  |  |  |  |
| **Involved in transmembrane transport, cellular homeostasis and drug resistance** | | | | | |
| BBA_10187 | XM_008605284.1 | -3.939 | 9.42E-11 | down | urea active transporte |
| BBA_07917 | XM_008603014.1 | -3.836 | 8.30E-10 | down | Major facilitator superfamily, general substrate transporter |
| BBA_03202 | XM_008598299.1 | -3.808 | 2.42E-14 | down | transporter-like protein |
| BBA_10301 | XM_008605398.1 | -2.751 | 1.64E-08 | down | major facilitator superfamily transporter |
| BBA_00968 | XM_008596065.1 | -2.445 | 5.74E-08 | down | phosphate transporter |
| BBA_00037 | XM_008595134.1 | -2.346 | 2.71E-05 | down | sodium/hydrogen exchanger family protein |
| BBA_07652 | XM_008602749.1 | -2.224 | 2.26E-05 | down | OPT oligopeptide transporter |
| BBA_06475 | XM_008601572.1 | -2.032 | 1.77E-05 | down | C4-dicarboxylate transporter/malic acid transporter |
| BBA_08789 | XM_008603886.1 | -2.017 | 5.29E-04 | down | choline transport protein |
| BBA_09459 | XM_008604556.1 | -1.990 | 1.44E-02 | down | major facilitator superfamily transporter |
| BBA_05741 | XM_008600838.1 | -1.916 | 1.18E-04 | down | vacuolar iron transporter Ccc1 |
| BBA_07285 | XM_008602382.1 | -1.915 | 3.73E-05 | down | canalicular multispecific organic anion transporter 1 |
| MSTRG.3760 | MSTRG.3760.1 | -1.913 | 8.73E-04 | down | OPT oligopeptide transporter |
| BBA_07854 | XM_008602951.1 | -1.897 | 6.38E-04 | down | sodium symporter family protein |
| BBA_02152 | XM_008597249.1 | -1.887 | 4.27E-05 | down | metabolite transport protein GIT1 |
| MSTRG.12616 | MSTRG.12616.1 | -1.839 | 6.76E-05 | down | phosphate:H+ symporter |
| BBA_08916 | XM_008604013.1 | -1.837 | 7.15E-04 | down | major facilitator superfamily transporter |
| BBA_06751 | XM_008601848.1 | -1.833 | 8.72E-04 | down | ABC transporter with duplicated ATPase domains |
| BBA_09418 | XM_008604515.1 | -1.684 | 4.88E-02 | down | MFS quinate transporter QutD |
| BBA_05155 | XM_008600252.1 | -1.645 | 1.01E-02 | down | allantoate permease |
| BBA_00212 | XM_008595309.1 | -1.608 | 1.48E-02 | down | major facilitator superfamily transporter |
| BBA_03110 | XM_008598207.1 | -1.573 | 9.02E-04 | down | multidrug resistance protein 1 |
| BBA_05874 | XM_008600971.1 | -1.548 | 4.87E-03 | down | MFS drug efflux transporter, putative |
| BBA_07525 | XM_008602622.1 | -1.541 | 3.39E-02 | down | calcium-transporting ATPase |
| BBA_08764 | XM_008603861.1 | -1.512 | 1.57E-03 | down | MFS transporter |
| BBA_09447 | XM_008604544.1 | -1.475 | 5.18E-03 | down | ABC transporter |
| BBA_04936 | XM_008600033.1 | -1.459 | 9.82E-03 | down | allantoate permease |
| MSTRG.4787 | MSTRG.4787.1 | -1.420 | 3.89E-03 | down | choline transporter |
| BBA_09470 | XM_008604567.1 | -1.420 | 1.98E-02 | down | MFS multidrug transporter, putative |
| BBA_10010 | XM_008605107.1 | -1.391 | 4.54E-03 | down | MFS multidrug transporter |
| BBA_03179 | XM_008598276.1 | -1.381 | 1.15E-02 | down | major facilitator superfamily transporter |
| BBA_04743 | XM_008599840.1 | -1.361 | 3.49E-02 | down | nitrite transporter |
| BBA_00666 | XM_008595763.1 | -1.358 | 6.81E-03 | down | Bicarbonate transporter |
| BBA_09774 | XM_008604871.1 | -1.353 | 3.47E-02 | down | glucose/galactose transporter |
| BBA_07970 | XM_008603067.1 | -1.350 | 9.23E-03 | down | MFS transporter |
| BBA_07120 | XM_008602217.1 | -1.340 | 8.18E-03 | down | methionine transporter, putative |
| BBA_09281 | XM_008604378.1 | -1.336 | 6.63E-03 | down | OPT peptide transporter Mtd1 |
| MSTRG.12133 | MSTRG.12133.1 | -1.333 | 1.58E-02 | down | hexose transporter |
| BBA_09449 | XM_008604546.1 | -1.321 | 4.35E-02 | down | MFS transporter |
| BBA_08947 | XM_008604044.1 | -1.289 | 3.30E-02 | down | potassium/sodium efflux P-type ATPase |
| MSTRG.4104 | MSTRG.4104.1 | -1.286 | 4.47E-02 | down | pantothenate transporter |
| MSTRG.8451 | MSTRG.8451.1 | -1.285 | 2.09E-02 | down | potassium/sodium efflux P-type ATPase |
| MSTRG.14397 | MSTRG.14397.1 | -1.252 | 1.26E-02 | down | ABC transporter |
| BBA_08447 | XM_008603544.1 | -1.250 | 1.51E-02 | down | Major Facilitator Superfamily protein |
| MSTRG.10291 | MSTRG.10291.1 | -1.225 | 1.97E-02 | down | vacuolar calcium ion transporter |
| BBA_09280 | XM_008604377.1 | -1.221 | 1.59E-02 | down | Ni^2+^-Co^2+^ transporter transition metal uptake transporter |
| BBA_02876 | XM_008597973.1 | -1.163 | 2.75E-02 | down | polyol transporter 5 |
| MSTRG.897 | MSTRG.897.1 | -1.153 | 3.76E-02 | down | MFS allantoate transporter, putative |
| BBA_05337 | XM_008600434.1 | -1.087 | 4.04E-02 | down | heavy-metal-associated domain-containing protein |
| BBA_09471 | XM_008604568.1 | -1.072 | 4.74E-02 | down | magnesium and cobalt transporter CorA |
| BBA_02053 | XM_008597150.1 | 1.079 | 4.99E-02 | up | cation efflux family protein |
| BBA_03446 | XM_008598543.1 | 1.121 | 4.39E-02 | up | MFS transporter |
| BBA_01825 | XM_008596922.1 | 1.135 | 3.72E-02 | up | siderochrome-iron transporter MirB |
| BBA_02808 | XM_008597905.1 | 1.197 | 2.68E-02 | up | ABC1 family protein |
| BBA_07748 | XM_008602845.1 | 1.230 | 1.78E-02 | up | ABC transporter |
| BBA_06070 | XM_008601167.1 | 1.248 | 3.36E-02 | up | ABC multidrug transporter |
| BBA_00207 | XM_008595304.1 | 1.269 | 1.21E-02 | up | cation diffusion facilitator family transporter |
| BBA_03670 | XM_008598767.1 | 1.327 | 7.00E-03 | up | MFS transporter, putative |
| BBA_06336 | XM_008601433.1 | 1.358 | 5.72E-03 | up | hexose transporter-like protein |
| BBA_09356 | XM_008604453.1 | 1.368 | 9.51E-03 | up | Major Facilitator Superfamily protein |
| BBA_05051 | XM_008600148.1 | 1.392 | 6.04E-03 | up | MIP family channel protein |
| BBA_05833 | XM_008600930.1 | 1.437 | 4.45E-03 | up | major facilitator superfamily transporter |
| BBA_04973 | XM_008600070.1 | 1.470 | 2.15E-03 | up | MFS transporter |
| BBA_08180 | XM_008603277.1 | 1.480 | 1.91E-03 | up | MFS multidrug resistance transporter, putative |
| BBA_01781 | XM_008596878.1 | 1.507 | 1.50E-03 | up | ABC-2 type transporter |
| BBA_07141 | XM_008602238.1 | 1.522 | 2.87E-03 | up | MFS transporter, putative |
| BBA_01236 | XM_008596333.1 | 1.552 | 1.22E-03 | up | major facilitator superfamily transporter |
| BBA_03815 | XM_008598912.1 | 1.557 | 4.89E-02 | up | formate/nitrate family transporter |
| BBA_09347 | XM_008604444.1 | 1.579 | 1.36E-02 | up | MFS transporter, putative |
| BBA_06806 | XM_008601903.1 | 1.631 | 1.44E-03 | up | multidrug resistance protein MDR, putative |
| BBA_00423 | XM_008595520.1 | 1.641 | 2.24E-03 | up | multidrug and toxin extrusion protein |
| BBA_06867 | XM_008601964.1 | 1.643 | 8.11E-04 | up | major facilitator superfamily transporter |
| BBA_02864 | XM_008597961.1 | 1.702 | 5.89E-04 | up | heavy metal translocating P-type ATPase |
| BBA_09123 | XM_008604220.1 | 1.800 | 2.01E-04 | up | multidrug resistance protein MDR, putative |
| BBA_09719 | XM_008604816.1 | 1.800 | 1.55E-04 | up | MFS toxin efflux pump (AflT) |
| BBA_06206 | XM_008601303.1 | 1.834 | 2.09E-02 | up | vacuolar protein sorting-associated protein 62 |
| BBA_08473 | XM_008603570.1 | 1.909 | 1.66E-04 | up | siderophore iron transporter mirA |
| BBA_09079 | XM_008604176.1 | 1.910 | 6.41E-05 | up | major facilitator superfamily protein |
| BBA_00896 | XM_008595993.1 | 1.954 | 1.78E-05 | up | major facilitator superfamily transporter |
| BBA_05015 | XM_008600112.1 | 1.977 | 1.50E-05 | up | multidrug resistance protein CDR1 |
| BBA_09592 | XM_008604689.1 | 2.055 | 1.55E-05 | up | major facilitator superfamily transporter |
| MSTRG.12 | MSTRG.12.1 | 2.298 | 2.59E-07 | up | ABC transporter transmembrane region |
| BBA_04690 | XM_008599787.1 | 2.301 | 2.39E-07 | up | Na,H/K antiporter P-type ATPase |
| BBA_05616 | XM_008600713.1 | 2.343 | 5.47E-07 | up | major facilitator superfamily transporter |
| BBA_02215 | XM_008597312.1 | 2.351 | 2.02E-07 | up | monocarboxylate transporter |
| BBA_00005 | XM_008595102.1 | 2.413 | 5.27E-08 | up | ABC transporter transmembrane region [ |
| BBA_06212 | XM_008601309.1 | 2.464 | 3.34E-04 | up | MFS monocarboxylate transporter |
| BBA_10298 | XM_008605395.1 | 2.682 | 4.16E-05 | up | ABC transporter, transmembrane domain, type 1 |
| BBA_03662 | XM_008598759.1 | 2.939 | 1.79E-07 | up | multidrug resistance protein MDR, putative |
| BBA_04706 | XM_008599803.1 | 2.977 | 1.73E-08 | up | MFS multidrug resistance transporter |
| BBA_07777 | XM_008602874.1 | 3.026 | 4.38E-10 | up | ABC metal ion transporter |
| MSTRG.4801 | MSTRG.4801.1 | 3.131 | 5.50E-09 | up | putative ABC transporter |
| BBA_02932 | XM_008598029.1 | 3.296 | 1.29E-04 | up | General substrate transporter |
| BBA_03053 | XM_008598150.1 | 3.383 | 1.44E-05 | up | abc transporter |
| BBA_06804 | XM_008601901.1 | 4.305 | 2.30E-04 | up | MFS transporter, putative |
| BBA_04498 | XM_008599595.1 | 5.031 | 1.49E-02 | up | putative sugar transporter |
| BBA_01614 | XM_008596711.1 | 5.256 | 6.07E-03 | up | MFS multidrug resistance transporter |
| BBA_04647 | XM_008599744.1 | 5.505 | 1.45E-18 | up | H(+)-myo-inositol cotransporter |
| MSTRG.13363 | MSTRG.13363.1 | 7.130 | 3.69E-26 | up | major facilitator superfamily transporter |
| BBA_09590 | XM_008604687.1 | 7.333 | 7.86E-10 | up | major facilitator superfamily transporter |
|  |  |  |  |  |  |
| **Involved in transcriptional regulation** | | |  |  |  |
| MSTRG.13959 | MSTRG.13959.1 | -2.854 | 6.47E-05 | down | C6 transcription factor |
| BBA_02379 | XM_008597476.1 | -2.605 | 1.13E-05 | down | C6 finger domain protein, putative |
| BBA_10040 | XM_008605137.1 | -2.545 | 8.62E-08 | down | helix-loop-helix DNA-binding domain-containing protein |
| MSTRG.8081 | MSTRG.8081.1 | -1.954 | 7.47E-03 | down | transcriptional regulator, putative |
| MSTRG.12594 | MSTRG.12594.1 | -1.827 | 1.20E-04 | down | C6 finger domain protein |
| BBA_03909 | XM_008599006.1 | -1.773 | 1.24E-04 | down | trascription factor |
| BBA_06319 | XM_008601416.1 | -1.700 | 1.07E-02 | down | Pre-mRNA-splicing factor srp2 |
| BBA_01665 | XM_008596762.1 | -1.695 | 3.24E-03 | down | C6 finger domain protein |
| BBA_07771 | XM_008602868.1 | -1.667 | 4.13E-04 | down | zinc finger protein |
| BBA_04826 | XM_008599923.1 | -1.408 | 4.62E-03 | down | C2H2 type zinc finger domain-containing protein |
| MSTRG.9885 | MSTRG.9885.1 | -1.383 | 1.57E-02 | down | reverse transcriptase, RNaseH |
| BBA_04065 | XM_008599162.1 | -1.337 | 3.39E-02 | down | C6 transcription factor |
| MSTRG.10689 | MSTRG.10689.1 | -1.268 | 4.49E-02 | down | C6 zinc finger domain-containing protein |
| BBA_04044 | XM_008599141.1 | -1.199 | 2.18E-02 | down | transcriptional regulatory protein pro-1 |
| MSTRG.12261 | MSTRG.12261.1 | -1.178 | 2.95E-02 | down | C6 zinc finger domain protein |
| BBA_04958 | XM_008600055.1 | 1.068 | 4.74E-02 | up | RadR putative transcriptional regulator |
| BBA_09697 | XM_008604794.1 | 1.142 | 4.00E-02 | up | C2H2 transcription factor |
| BBA_03901 | XM_008598998.1 | 1.149 | 3.88E-02 | up | zinc finger protein 1 |
| BBA_09551 | XM_008604648.1 | 1.173 | 2.53E-02 | up | C6 transcription factor, putative |
| BBA_02404 | XM_008597501.1 | 1.235 | 2.48E-02 | up | Putative Zn(II)2Cys6 transcription factor |
| BBA_09641 | XM_008604738.1 | 1.262 | 1.31E-02 | up | Fungal Zn binuclear cluster domain containing protein |
| BBA_09617 | XM_008604714.1 | 1.270 | 3.58E-02 | up | NFX1-type zinc finger-containing protein 1 |
| BBA_07054 | XM_008602151.1 | 1.297 | 2.22E-02 | up | C6 zinc finger domain-containing protein |
| BBA_00071 | XM_008595168.1 | 1.315 | 1.76E-02 | up | nitrate assimilation regulatory protein nirA |
| BBA_06121 | XM_008601218.1 | 1.338 | 8.58E-03 | up | C2H2 finger domain-containing protein |
| BBA_08458 | XM_008603555.1 | 1.402 | 5.72E-03 | up | two component transcriptional regulator, LuxR family |
| BBA_05123 | XM_008600220.1 | 1.461 | 4.40E-03 | up | Putative Zn(II)2Cys6 transcription factor |
| BBA_07667 | XM_008602764.1 | 1.473 | 1.79E-02 | up | reverse transcriptase |
| BBA_05939 | XM_008601036.1 | 1.502 | 2.57E-02 | up | fungal specific transcription factor |
| BBA_04778 | XM_008599875.1 | 1.566 | 2.31E-03 | up | transcriptional repressor TUP1 |
| BBA_09298 | XM_008604395.1 | 1.595 | 1.51E-03 | up | Zn(II)2Cys6 transcription factor |
| BBA_04702 | XM_008599799.1 | 1.666 | 7.52E-04 | up | C6 transcription factor |
| BBA_04295 | XM_008599392.1 | 1.680 | 4.03E-03 | up | C2 domain-containing protein |
| BBA_09314 | XM_008604411.1 | 1.723 | 2.83E-02 | up | Putative Zn(II)2Cys6 transcription factor |
| BBA_01117 | XM_008596214.1 | 1.789 | 6.25E-04 | up | C6 zinc finger domain-containing protein |
| BBA_09821 | XM_008604918.1 | 1.814 | 1.21E-03 | up | translation factor (SUA5) |
| MSTRG.4577 | MSTRG.4577.1 | 2.081 | 1.04E-02 | up | transcription-coupled repair protein CSB/RAD26 |
| BBA_04646 | XM_008599743.1 | 2.339 | 6.44E-07 | up | C6 zinc finger domain protein |
| BBA_09924 | XM_008605021.1 | 2.722 | 1.65E-09 | up | zinc finger protein |
| BBA_08967 | XM_008604064.1 | 2.768 | 1.50E-02 | up | fungal zinc cluster transcription factor |
| BBA_05678 | XM_008600775.1 | 2.953 | 2.80E-05 | up | nitrogen assimilation transcription factor nirA |
| BBA_10233 | XM_008605330.1 | 3.294 | 6.08E-10 | up | sexual development transcription factor NsdD |
| BBA_09423 | XM_008604520.1 | 3.324 | 7.56E-05 | up | translation factor (SUA5) |
| BBA_07418 | XM_008602515.1 | 3.398 | 4.77E-11 | up | fungal specific transcription factor |
| BBA_09628 | XM_008604725.1 | 4.722 | 4.15E-08 | up | C6 zinc finger domain-containing protein |
| BBA_09926 | XM_008605023.1 | 4.923 | 2.34E-02 | up | NF-X1 finger and helicase domain protein |
| BBA_09403 | XM_008604500.1 | 4.965 | 1.57E-06 | up | bZIP transcription factor |
| BBA_07342 | XM_008602439.1 | 5.053 | 1.49E-02 | up | C6 transcription factor |
| BBA_10253 | XM_008605350.1 | 5.775 | 1.09E-25 | up | sexual development transcription factor NsdD |
| BBA_02766 | XM_008597863.1 | 6.417 | 4.33E-06 | up | BTB domain transcription factor |
|  |  |  |  |  |  |
| **Involved in posttranslation modifications** | | |  |  |  |
| BBA_08040 | XM_008603137.1 | -4.794 | 3.71E-02 | down | HemK family methyltransferase |
| BBA_04607 | XM_008599704.1 | -4.593 | 3.07E-19 | down | Acyl-CoA N-acyltransferase |
| BBA_00688 | XM_008595785.1 | -2.992 | 4.36E-03 | down | UbiA prenyltransferase |
| BBA_08073 | XM_008603170.1 | -2.495 | 2.54E-06 | down | phosphotransferase enzyme family protein |
| BBA_02776 | XM_008597873.1 | -2.424 | 4.77E-04 | down | serine/threonine protein kinase |
| BBA_01015 | XM_008596112.1 | -2.305 | 4.10E-07 | down | methyltransferase-like protein |
| BBA_05439 | XM_008600536.1 | -1.995 | 1.09E-05 | down | phosphorylcholine phosphatase |
| BBA_07463 | XM_008602560.1 | -1.778 | 1.57E-02 | down | putative SAM-dependent methyltransferase |
| BBA_05559 | XM_008600656.1 | -1.769 | 6.70E-04 | down | Acyl-CoA N-acyltransferase |
| BBA_05790 | XM_008600887.1 | -1.693 | 4.60E-04 | down | sterigmatocystin 8-O-methyltransferase precursor, putative |
| BBA_06473 | XM_008601570.1 | -1.656 | 1.56E-03 | down | Acyl-CoA N-acyltransferase |
| BBA_04798 | XM_008599895.1 | -1.595 | 7.05E-04 | down | methyltransferase domain-containing protein |
| BBA_01701 | XM_008596798.1 | -1.426 | 3.98E-03 | down | acetyltransferase, GNAT family protein |
| MSTRG.972 | MSTRG.972.1 | -1.315 | 1.47E-02 | down | serine/threonine kinase |
| BBA_05282 | XM_008600379.1 | -1.283 | 1.75E-02 | down | Histone H5 |
| BBA_05603 | XM_008600700.1 | -1.254 | 3.96E-02 | down | isoprenylcysteine carboxyl methyltransferase |
| BBA_04899 | XM_008599996.1 | -1.188 | 2.42E-02 | down | SH3 domain-containing protein |
| BBA_04152 | XM_008599249.1 | -1.156 | 2.57E-02 | down | phospholipid methyltransferase |
| MSTRG.5332 | MSTRG.5332.1 | 1.181 | 2.16E-02 | up | O-methyltransferase family protein |
| BBA_07721 | XM_008602818.1 | 1.201 | 2.09E-02 | up | phosphotransferase enzyme family protein |
| BBA_00764 | XM_008595861.1 | 1.205 | 3.04E-02 | up | ubiquitin-conjugating enzyme E2 |
| BBA_02913 | XM_008598010.1 | 1.240 | 4.17E-02 | up | methyltransferase domain-containing protein |
| BBA_06971 | XM_008602068.1 | 1.432 | 3.34E-03 | up | methyltransferase-like protein |
| BBA_09913 | XM_008605010.1 | 1.589 | 7.91E-03 | up | SET domain-containing protein 5 |
| BBA_04585 | XM_008599682.1 | 1.730 | 2.17E-02 | up | elongation factor-2 kinase EFK-1B isoform |
| BBA_07774 | XM_008602871.1 | 1.856 | 4.67E-04 | up | serine/threonine protein kinase |
| BBA_10351 | XM_008605448.1 | 1.878 | 4.02E-03 | up | serine/threonine protein kinase Japonica Group |
| BBA_05117 | XM_008600214.1 | 2.071 | 1.73E-02 | up | SET domain-containing protein |
| BBA_05309 | XM_008600406.1 | 2.085 | 1.34E-04 | up | phosphotransferase enzyme family protein |
| BBA_07844 | XM_008602941.1 | 2.255 | 2.47E-06 | up | O-methyltransferase, family 3 |
| BBA_08686 | XM_008603783.1 | 2.812 | 2.17E-10 | up | UDP-glucosyltransferase |
| BBA_08685 | XM_008603782.1 | 2.872 | 9.26E-11 | up | FkbM family methyltransferase |
| BBA_08218 | XM_008603315.1 | 2.908 | 4.29E-08 | up | Acyl-CoA N-acyltransferase |
| BBA_01386 | XM_008596483.1 | 3.103 | 1.07E-02 | up | histone H2A, putative |
| MSTRG.11519 | MSTRG.11519.1 | 3.546 | 9.20E-05 | up | Acyl-CoA N-acyltransferase |
| BBA_03667 | XM_008598764.1 | 3.737 | 4.18E-16 | up | methyltransferase domain-containing protein |
| MSTRG.13424 | MSTRG.13424.1 | 3.876 | 1.07E-04 | up | phosphotransferase family protein |
| BBA_04783 | XM_008599880.1 | 4.385 | 2.41E-10 | up | UDP-N-acetylmuramate--L-alanine ligase |
| BBA_02552 | XM_008597649.1 | 4.799 | 3.71E-02 | up | serine/threonine protein kinase |
| BBA_05308 | XM_008600405.1 | 5.915 | 1.91E-21 | up | acyltransferase-like protein |
| BBA_10255 | XM_008605352.1 | 6.434 | 3.73E-30 | up | Methyltransferase type 11 |
| BBA_05311 | XM_008600408.1 | 10.613 | 6.13E-32 | up | UDP-N-acetylglucosamine acyltransferase |

**Table S8.** Paired primers used for qPCR analysis of 14 DEGs to validate transcriptomic data.

| Tag locus* | Annotation | Sequences (5′−3′) of paired primers |
| --- | --- | --- |
| BBA_00049 | Intracellular serine protease | AGAGGACGATAAGCCCCATT / CTTGCTTCCCTTTTCTGTCG |
| BBA_00428 | Fatty acid hydroxylase superfamily protein | ACTTGCTGCTCCGAGACATT / CGTGTACGAGCCGTTACAAA |
| BBA_06599 | Adhesin protein Mad2 | TGTCAAGACTGGCGACATTT / ATTGGGACAAGCTGGTTGAG |
| BBA_09825 | Cutinase-like protein | AGCTCGATGCTCTTCATGGT / ACCTCCGTAAACTGGCAATG |
| BBA_04401 | Sporulation-specific protein | TGGACAGTTCGTTCTTGCAC / GGTTTTCAAGGCCAGGTACA |
| BBA_02876 | Vivid PAS domain protein VVD | TAGATTGTTCTGCGGCACTG / ATGTGTTGGTCGAGGAGAGG |
| BBA_01528 | Frequency clock protein Frq1 | TCTTTGTTCGGCAGGACTCT / GCCAGACAAATCAGTGCAGA |
| BBA_07286 | Hydrophobin-like protein | TCATTCCTCCTGTTGCTCCT / GGCCTCGATGAACATGAGAT |
| BBA_02996 | Antigenic cell wall galactomannoprotein | CCAAAGGAGCTGGCTGATGT / AAGAAAACGCCTTGATGCCC |
| BBA_09822 | Concanavalin A-like lectin/glucanase | GACCTACGATGCCTCCAACT / TTGGATTGGACGCGTAACGA |
| BBA_03202 | Transporter-like protein | ATCCTGACCACTCCATCTCG / TTTGGAGCACCTTTTCATCC |
| BBA_09423 | Translation factor (SUA5) | TACTCGGGCTTTGACGGAAG / TGTTGGAGCGTGGTCTTGTC |
| BBA_07418 | Fungal specific transcription factor | ACCGCTTCATTGTCCAATTC / GCTCCTTGGCAAATGGTAAA |

* Gene accession codes of *B. bassiana* genome under the NCBI accession NL_ADAH00000000.
